# Hydroxy Groups Enhance [2]Rotaxane Anion Binding Selectivity

**DOI:** 10.1002/chem.202200389

**Published:** 2022-04-05

**Authors:** Rosemary J. Goodwin, Andrew Docker, Hugo I. MacDermott‐Opeskin, Heather M. Aitken, Megan L. O'Mara, Paul D. Beer, Nicholas G. White

**Affiliations:** ^1^ Research School of Chemistry Australian National University Canberra ACT Australia; ^2^ Department of Chemistry University of Oxford Chemistry Research Laboratory Mansfield Road Oxford OX1 3TA UK

**Keywords:** anions, hydroxy groups, molecular dynamics, preorganisation, rotaxanes

## Abstract

We report the synthesis of two [2]rotaxanes containing an interlocked three dimensional binding cavity formed from a pyridinium bis(amide) axle component containing two phenol donors, and an isophthalamide based macrocycle. In the competitive solvent mixture 1 : 1 CDCl_3_ : CD_3_OD, one of the receptors exhibits a much higher selectivity preference for chloride than an analogous rotaxane without the hydroxy groups. X‐ray crystal structures reveal the chloride anion guest encapsulated within the interlocked binding cavity, though not all of the hydrogen bond donors are utilised. Computational semi‐empirical simulations indicate that secondary intermolecular interactions occur between the axle hydroxy hydrogen bond donors and the [2]rotaxane macrocycle components, contributing to a more preorganised binding pocket, which may be responsible for the observed enhanced selectivity.

## Graphical abstract

**Two new rotaxanes** were synthesized containing phenolic O‐H groups on the axle component. These O‐H groups hydrogen bond to the macrocycle component, rigidifying the binding pocket and resulting in significantly enhanced selectivity for chloride anions over more basic oxoanions.

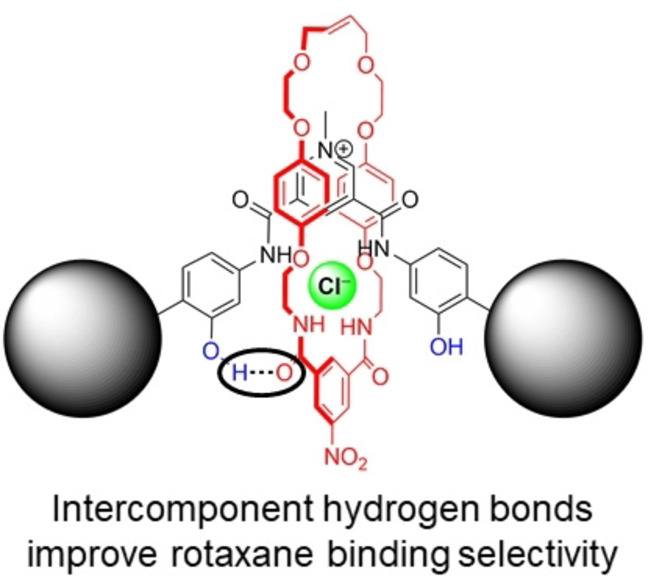

## Introduction

Anions are involved in nearly all aspects of life, with many enzyme substrates and cofactors being negatively charged.^[1]^ As well as anionic substrates, small inorganic anions play an extensive role throughout the body, with diseases such as cystic fibrosis being linked to the mis‐regulation of chloride in extracellular fluid^[2]^ and bicarbonate being essential to regulate pH levels in the body.^[3]^ Issues also arise from the extensive use of nitrate and phosphate based fertilizers resulting in the eutrophication of waterways.^[4]^ The presence of anions in both environmental pollution and essential bodily functions highlights the need to investigate the binding and sensing of these species. However, there are various characteristics of anions that make them more difficult to bind than cations, as they have a decreased charge to radius ratio,^[5]^ a variety of geometries,^[6]^ higher solvation energies^[7]^ and increased pH sensitivity.^[8]^

**Figure 1:**
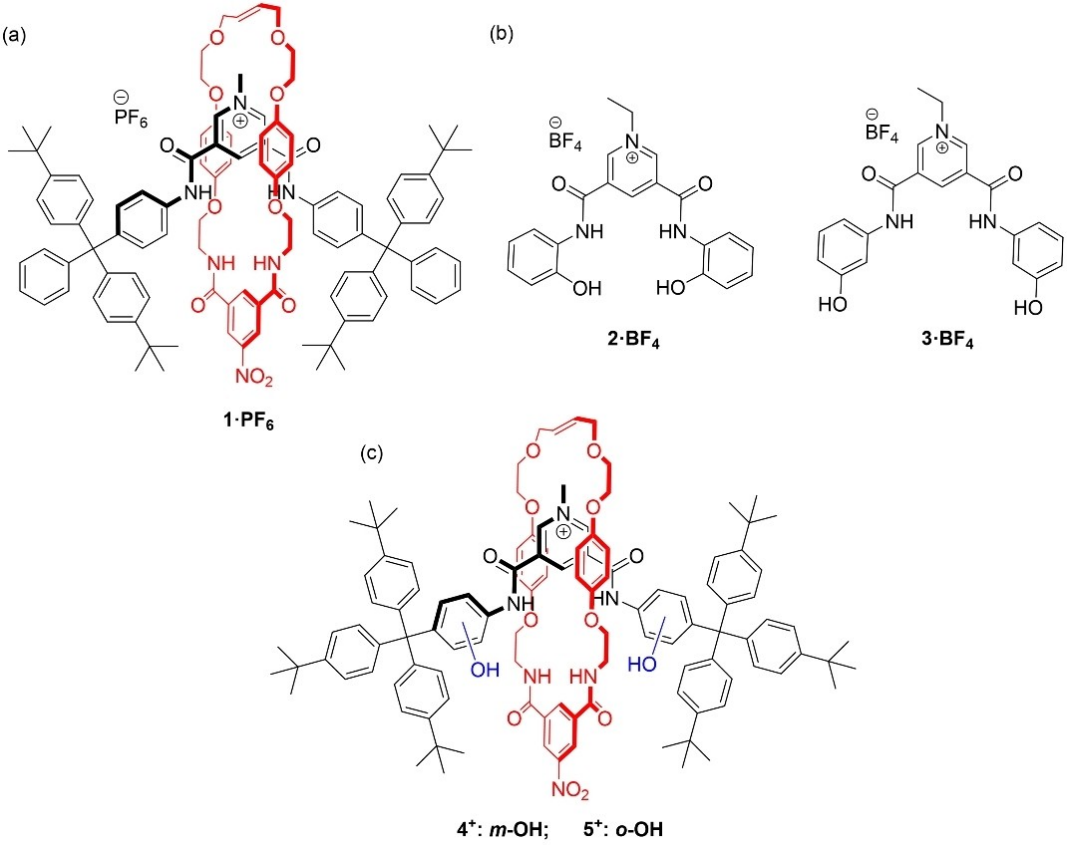
Structures of a) previously reported rotaxane **1 ⋅ PF_6_**, b) hydroxy containing receptors **2 ⋅ BF_4_** and **3 ⋅ BF_4_** and c) new rotaxanes reported in this work.

By virtue of their inherent three‐dimensional cavities, interlocked molecules have been shown to be potent and selective hosts for anion guest species in competitive protic solvent media.[^9^, ^10^, ^11^, ^12^] This is highlighted by Beer and co‐workers’ [2]rotaxane **1 ⋅ PF_6_** (Figure 1a) prepared via chloride anion templation,^[13]^ which binds chloride strongly in CDCl_3_ : CD_3_OD, and shows selectivity for this anion over more basic oxoanions. This selectivity arises due to the interlocked rotaxane's complementary sized cavity containing orthogonally arranged, convergent amide hydrogen bond donors. Notably, the acyclic non‐interlocked axle component shows the reverse oxoanion selectivity preference.^[13]^ Subsequent work has demonstrated that a variety of other anion recognition motifs including C−H hydrogen bond donors,[^14^, ^15^] halogen bond donors[^16^, ^17^, ^18^] and chalcogen bond donors^[19]^ can be incorporated into interlocked architectures, and result in strong and selective anion binding.

The use of O−H hydrogen bond donors for anion recognition has lagged behind that of N−H and even C−H donors,[^20^, ^21^] which is perhaps surprising given their facile synthesis and potent hydrogen bond donor characteristics (e.g. p*K*_a_s are typically approximately 10 for aromatic^[22]^ and 15 for aliphatic^[23]^ O−H groups, respectively). The relatively few O−H based receptor systems reported to date have demonstrated effective anion binding,[^24^, ^25^] anion transport[^26^, ^27^] and anion‐templated self‐assembly.^[28[NW1] ]^ These qualities indicate that an interlocked host cavity containing O−H hydrogen bond donors could be a potent anion recognition site.

We recently studied the simple cationic pyridinium receptors **2 ⋅ BF_4_** and **3 ⋅ BF_4_**, which contain phenolic O−H groups (Figure 1b).^[20]^ Both acyclic hosts bound a variety of anions in aqueous acetonitrile (90 : 10 CD_3_CN : D_2_O), with a preference being shown for sulfate. We also observed that **3^+^** displayed significantly stronger anion binding than an analogous pyridinium receptor without hydroxy groups. Interestingly, molecular dynamics simulations suggested that **3 ⋅ BF_4_** interacted with hydrated anions, with the receptor's O−H groups hydrogen bonding to water molecules, which then hydrogen bonded to the anion.

These promising results suggested that O−H hydrogen bond donor functionalised [2]rotaxanes would prove to be efficacious anion hosts in competitive protic solvent media, while the unusual binding of the hydrated anions may also be observed. This would be highly interesting as binding hydrated anions underpins the function of most membrane transporter proteins.^[29]^ Herein, we report the synthesis of bis‐phenolic O−H containing axle component [2]rotaxanes **4^+^** and **5^+^** (Figure 1c), one of which exhibits enhanced chloride anion binding strength and selectivity in comparison to an analogous rotaxane without the O−H groups.

## Results and Discussion

### Synthesis

**Scheme 1:**
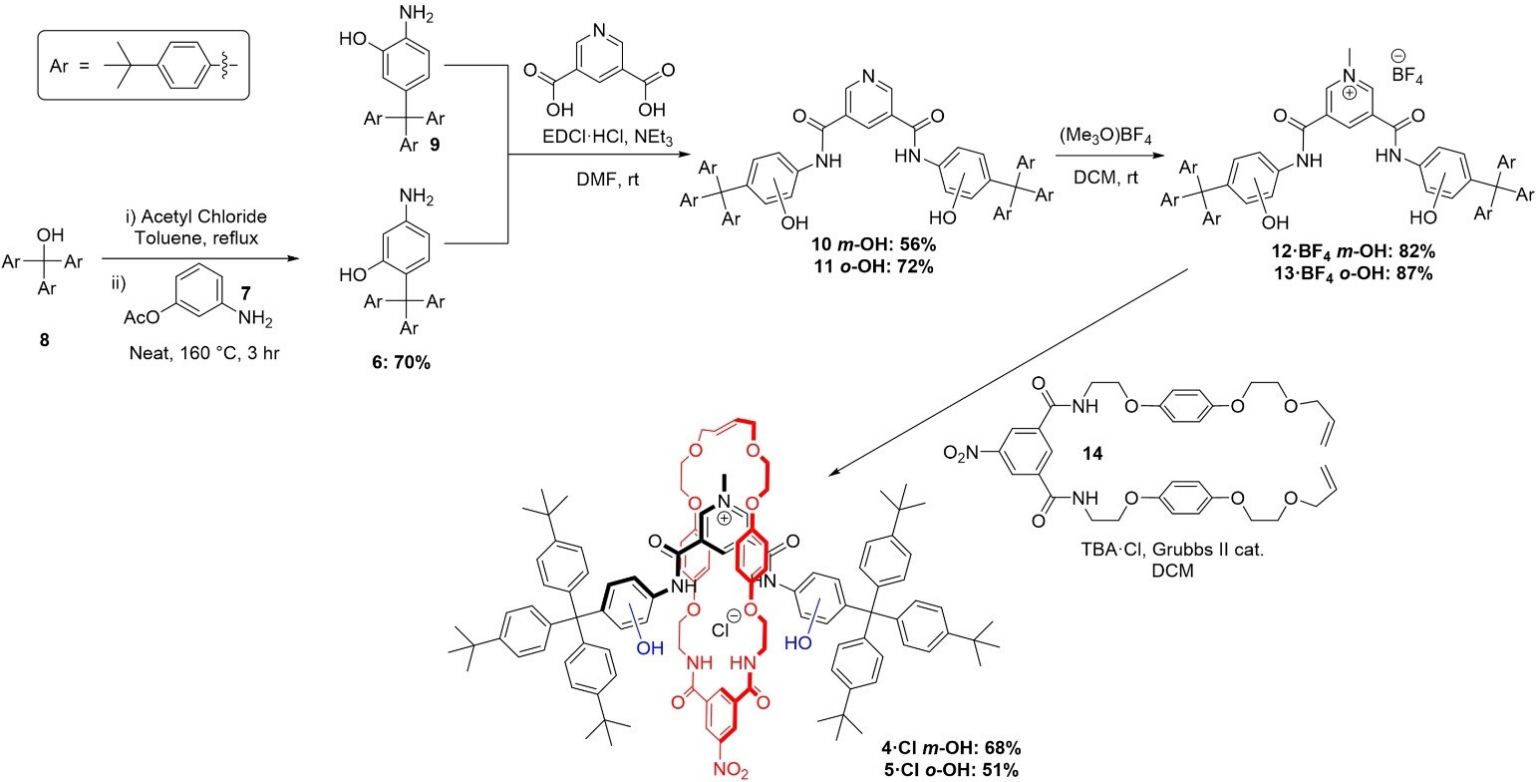
Synthesis of [2]rotaxanes **4 ⋅ Cl** and **5 ⋅ Cl**.^[33]^

In order to prepare the target rotaxanes **4^+^** and **5^+^**, it was necessary to synthesise appropriate “stopper” components containing amine and hydroxy substituents (Scheme 1). Initially acid catalysed electrophilic aromatic substitution of 3‐aminophenol and tris(*p‐t‐*butylphenyl)methanol (**8**) was investigated in an attempt to form *meta‐*aminophenol stopper **6**. However due to difficulties achieving reproducible reaction outcomes, alternative synthetic methods were explored (see Supporting Information for more information on difficulties encountered). To favour substitution *para*‐ to the amine group, the hydroxy group was acetyl protected to give compound **7**. This was then heated with tris(*p‐t‐*butylphenyl)methyl chloride, which was itself prepared from alcohol **8** and used immediately.^[30]^ Acetyl deprotection of the stopper occurred in situ, giving compound **6** in a yield of 70 %. The *ortho*‐aminophenol stopper **9** was synthesized from **8** as reported by MacLachlan and co‐workers.^[31]^

The *meta*‐phenol stopper **6** and *ortho*‐phenol stopper **9** were then coupled with 3,5‐pyridinedicarboxylic acid to form the neutral axle components **10** and **11** in 56 and 72 % yields, respectively. Methylation of the central pyridine group using trimethyloxonium tetrafluoroborate afforded the cationic axles **12 ⋅ BF_4_** and **13 ⋅ BF_4_** in high yields and without significant competing *O*‐methylation. The desired [2]rotaxanes **4 ⋅ Cl** and **5 ⋅ Cl** were then prepared by chloride anion‐templated Grubbs’ catalysed RCM clipping reactions of the bis‐vinyl functionalized macrocycle precursor **14** around the charged axles **12 ⋅ BF_4_** or **13 ⋅ BF_4_**_._ Using an excess (5 equiv.) of **14** provided the [2]rotaxanes in good yields (68 and 51 % for **4 ⋅ Cl** and **5 ⋅ Cl**, respectively).[^32^, ^33^] The rotaxanes were purified by preparative thin layer chromatography and characterized by ^1^H and ^13^C NMR spectroscopy, high resolution ESI mass spectrometry and X‐ray crystallography (see later).

In order to conduct anion binding studies, the chloride salts were exchanged for non‐coordinating anions to give **4 ⋅ PF_6_** and **5 ⋅ PF_6_** in quantitative yields. Complete anion exchange was confirmed using quantitative ^19^F NMR spectroscopy against a trifluoroethanol standard (see Supporting Information for details).

### Anion recognition studies

**Figure 2:**
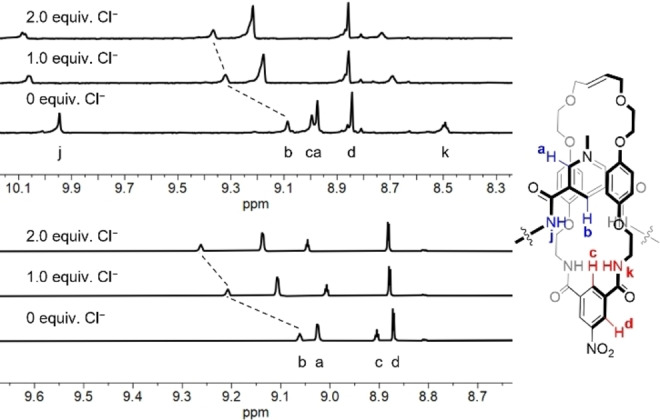
Truncated ^1^H NMR spectra of **4 ⋅ PF_6_** (top) and **5 ⋅ PF_6_** (bottom) upon addition of TBA⋅Cl (2.0 mM of rotaxane in 1 : 1 CDCl_3_ : CD_3_OD, 600 MHz, 298 K).

**Figure 3:**
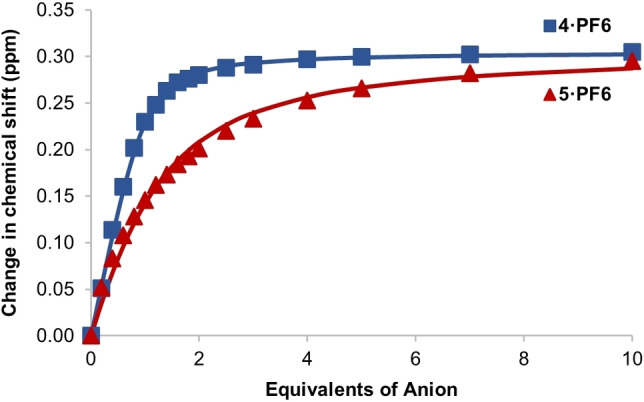
Movement of interior pyridinium C−H proton resonance of **4 ⋅ PF_6_** and **5 ⋅ PF_6_** upon addition of TBA ⋅ Cl in 1 : 1 CDCl_3_ : CD_3_OD. Points represent observed data, lines represent 1 : 1 binding isotherms fitted using *Bindfit*.^[34]^

The anion binding capabilities of the [2]rotaxanes were studied using ^1^H NMR titration experiments. Anions as their tetrabutylammonium (TBA) salts were added to solutions of **4 ⋅ PF_6_** and **5 ⋅ PF_6_** in 1 : 1 CDCl_3_ : CD_3_OD. This resulted in significant shifts of the axle components’ pyridinium C−H proton resonances and macrocycle components’ nitroisophthalamide C−H proton resonances (the O−H signals for both rotaxanes were not visible due to H/D exchange, while the broad N−H signals were initially visible but disappeared over the course of the titrations). Figures 2 and 3 show the effect of addition of chloride anion to **4 ⋅ PF_6_** and **5 ⋅ PF_6_** in 1 : 1 CDCl_3_ : CD_3_OD while spectra for the addition of all anions to both rotaxanes are provided in the Supporting Information.

*Bindfit*[^34^, ^35^] analysis of the interior pyridinium C−H proton titration data using either 1 : 1 or 2 : 1 host:guest binding models determined quantitative association constant values shown in Table 1 (all binding isotherms, weblinks to binding data and fits are provided in the Supporting Information).

**Table 1:** Association constants (M^‐1^) for addition of anions^[a]^ to receptors **4 ⋅ PF_6_**, **5 ⋅ PF_6_** and **1 ⋅ PF_6_** in 1 : 1 CDCl_3_ : CD_3_OD.

**Anion**	**4 ⋅ PF_6_**	**5 ⋅ PF_6_**	**1 ⋅ PF_6_** ^13, [b]^
Cl^−^	6.37(8)×10^3^	7.71(5)×10^2^	4.50×10^3^
I^−^	1.12(6)×10^3^	1.57(4)×10^2^	not measured
OAc^−^	4.00(8)×10^2^	K_11_=2.10(5)×10^2^	7.25×10^2^
		K_12_=1.75(6)×10^2^	
H_2_PO_4_^−^	1.22(8) ×10^2^	K_11_=1.81(11)×10^3^	1.50×10^3^
		K_12_=3.54(11)×10^2^	

[a] Anions added as TBA salts, binding constants determined using *Bindfit*,^[34]^ the asymptotic error^[35]^ is provided at the 95 % confidence interval in parentheses; [b] estimated errors less than 10 %.^[13]^

As shown in Table 1, *meta‐*substituted **4 ⋅ PF_6_** binds chloride with considerable affinity, notably surpassing that of iodide and basic acetate and dihydrogen phosphate. The *ortho*‐substituted analogue **5 ⋅ PF_6_**, exhibits a different anion selectivity profile to **4 ⋅ PF_6_**, and shows little selectivity and a considerably diminished *K*_a_(Cl^−^). Interestingly, unlike its *meta*‐substituted analogue, **5 ⋅ PF_6_** exhibits a 2 : 1 host:guest binding stoichiometry for AcO^−^ and H_2_PO_4_^−^, as determined by analysis of isotherm fit quality. This is presumably attributable to the steric inaccessibility of the binding cavity for these larger anion guest species. Based on the upfield chemical shift perturbations of the internal pyridinium C−H proton, this may indicate these binding events occur outside of the binding pocket to the [2]rotaxane‘s exterior.

We suggest that the affinity of **4 ⋅ PF_6_** and **5 ⋅ PF_6_** for chloride is due to the geometry and size of the anion, as was observed with **1 ⋅ PF_6_**, which does not contain any O‐H groups.^[13]^ However the contrast in binding strength between **5 ⋅ PF_6_** and **1 ⋅ PF_6_** implies that there may be competing processes that hinder the hydroxy‐containing [2]rotaxane from recognizing chloride despite it containing an increased number of hydrogen bond donors compared to **1 ⋅ PF_6_**. Contrary to this, **4 ⋅ PF_6_** is a more effective chloride selective receptor than both **1 ⋅ PF_6_** and **5 ⋅ PF_6_**, importantly suggesting that incorporation of *meta‐*hydroxy substituents assists in both binding affinity and selectivity.

**Figure 4:**
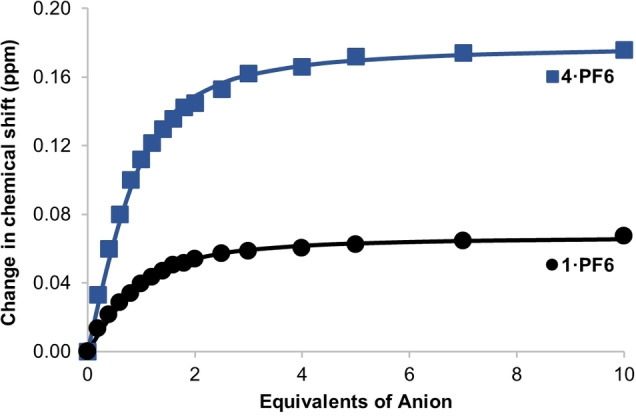
Movement of interior pyridinium C−H proton resonance of **4 ⋅ PF_6_** and **1 ⋅ PF_6_** upon addition of TBA ⋅ Cl in 2 : 49 : 49 D_2_O : CDCl_3_ : CD_3_OD. Points represent observed data, lines represent 1 : 1 binding isotherms fitted using *Bindfit*.^[34]^

Due to the strong and selective chloride anion binding behaviour exhibited by **4^+^** in a competitive organic solvent mixture, further anion recognition experiments were conducted with **4 ⋅ PF_6_** and **1 ⋅ PF_6_** in 2 : 49 : 49 D_2_O : CDCl_3_ : CD_3_OD (Figure 4). Unfortunately, due to solubility issues the water content could not be increased beyond 2 %. Addition of chloride to **4 ⋅ PF_6_** and **1 ⋅ PF_6_** resulted in downfield shifts of the interior pyridinium C−H proton resonances, and the data were fitted to a 1 : 1 binding isotherm using the program *Bindfit*.[^34^, ^35^] Both of the [2]rotaxanes bound chloride with a moderate affinity, with **4 ⋅ PF_6_** binding slightly more strongly than **1 ⋅ PF_6_** [*K*_a_=2.01(5)×10^3^ M^−1^ and 1.86(8)×10^3^ M^−1^ for **4 ⋅ PF_6_** and **1 ⋅ PF_6_** respectively].

### X‐ray crystallography

**Figure 5:**
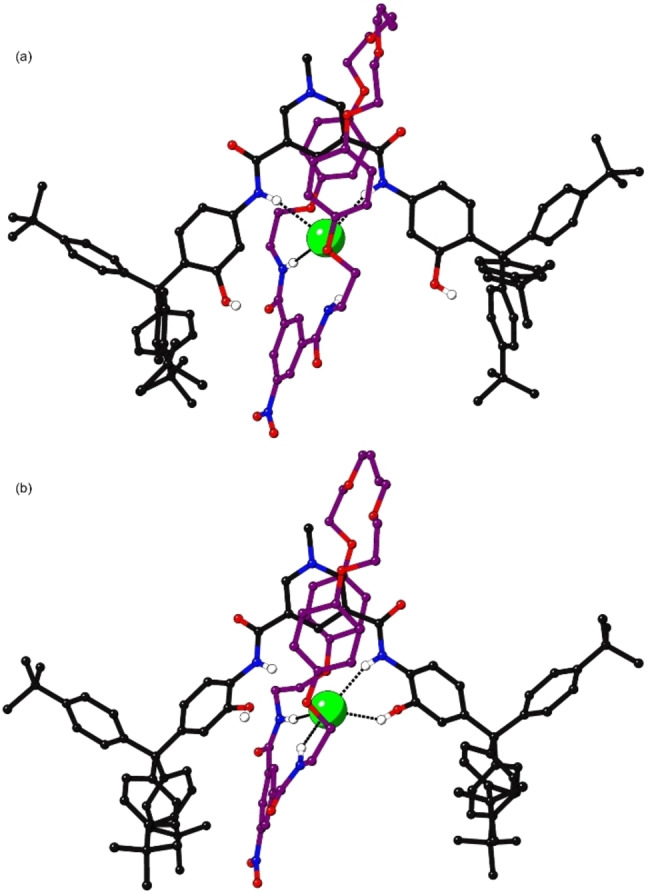
X‐ray crystal structure of a) **4 ⋅ Cl** and b) **5 ⋅ Cl**. Intermolecular hydrogen bonds are shown as dashed lines. Most hydrogen atoms and solvent molecules are omitted for clarity.

Single crystals of **4 ⋅ Cl** and **5 ⋅ Cl** were obtained by vapour diffusion of diethyl ether into a 1 : 1 CH_3_OH : CH_2_Cl_2_ solution of the [2]rotaxanes; subsequent analysis of the data generated using synchrotron X‐ray crystallography determined the structures shown in Figure 5. In both structures the chloride anion is bound within the 3D cavity of the interlocked host. Rotaxane **4 ⋅ Cl** shows only N−H⋅⋅⋅anion (2.48‐3.00 Å) and C−H⋅⋅⋅anion (2.81–2.82 Å) hydrogen bonding. In the solid state, there appears to be no O−H⋅⋅⋅anion hydrogen bonding, with these groups instead interacting with surrounding solvent. Rotaxane **5 ⋅ Cl** shows only three N−H⋅⋅⋅anion hydrogen bonds (2.44–2.50 Å) with one O−H⋅⋅⋅anion hydrogen bond also present (2.39 Å). The asymmetric bonding is due to one side of the axle twisting outwards, allowing the hydroxy group to hydrogen bond with the surrounding solvent.

### Semiempirical quantum mechanical simulations

To further investigate the influence of the presence and position of the hydroxy groups on rotaxane anion binding strength, computational semi‐empirical simulations of **1^+^**, **4^+^** and **5^+^** with the anions PF_6_^−^, Cl^−^ and OAc^−^ were undertaken using the GFN2‐xTB method implemented in xTB 6.4.0.^[36]^ This approach bridges a computational gap between empirical molecular dynamics simulations and ab initio quantum mechanical methods more suited to small system sizes. As mixed solvent systems are not handled by the GFN2‐xTB implicit solvent model, we used methanol as the solvent to best represent the competitive chloroform/methanol mixture used for anion sampling binding studies. While we believe this represents a reasonable approximation, it likely overestimates the competitiveness of the solvent, i.e. may reduce the favourability of binding relative to the solution experiments conducted in 1 : 1 CDCl_3_ : CD_3_OD (detailed analysis is provided in the Experimental Section and Supporting Information).

During the course of the simulations, it was found that all of the [2]rotaxane systems were highly dynamic, with various hydrogen bonding arrangements possible between the [2]rotaxane and the anion and between the components of the rotaxane (see Supporting Information for further information). When **5^+^** was studied, the dominant conformations involved significant hydrogen bonding between the rotaxane host and anion guest species, with the Cl^−^ or OAc^−^ anions typically interacting outside the rotaxane's cavity. This is consistent with the relatively weaker solution anion binding displayed by rotaxane **5^+^**.

**Figure 6:**
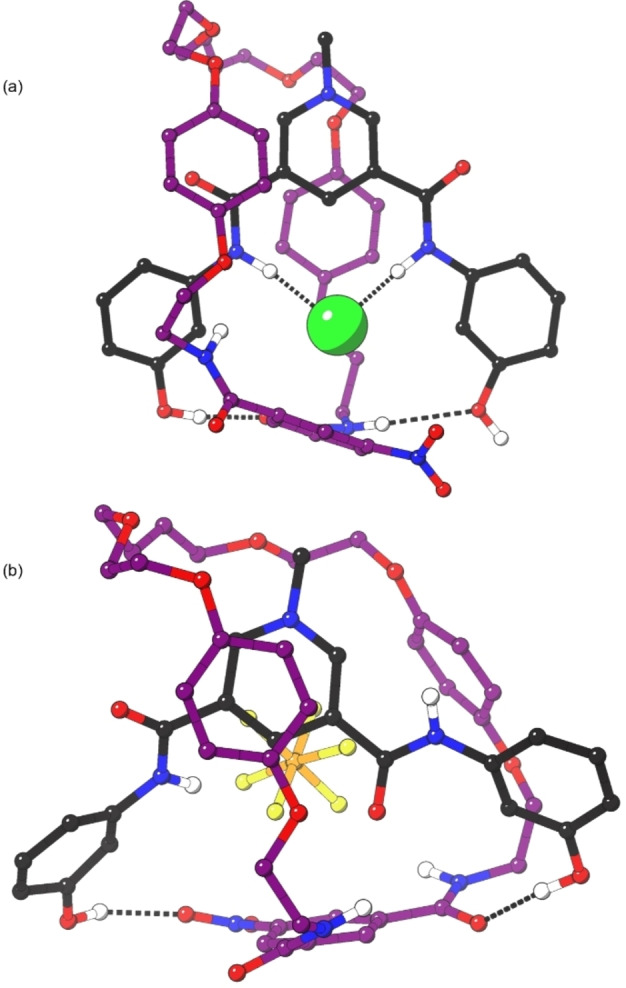
Representative structures from semiempirical simulations; (a) **4 ⋅ Cl** with anion bound within cavity; (b) **4 ⋅ PF_6_**; dotted black lines indicate hydrogen bond interactions (the sum of the van der Wall radii). Stoppers and most hydrogen atoms omitted for clarity.

When **1^+^** and **4^+^** were studied, the guest anion was located within the respective interlocked binding cavity far more commonly (57 % and 48 % of the simulation time respectively). Notably however, even though both rotaxanes bind chloride strongly, the simulations suggest that it is relatively unusual for all four amide donors to be hydrogen bonding to the anion, with this interaction occurring less than 7 % and 1 %, respectively, of the simulation time. Instead, there are often one or two amide donors hydrogen bonding to another component of the rotaxane, generally either the hydroxy groups or carbonyl oxygens. Perhaps surprisingly, the hydroxy donors present in the axle component of **4^+^** appear to rarely participate in endogenous anion binding. Instead, they commonly hydrogen bond to the carbonyl oxygen atoms of the macrocycle (Figure 6a) and this arrangement preorganises the two components of the rotaxane for anion binding. This additional inter‐component preorganisation may explain the increased chloride binding strength observed for **4^+^** compared with **1^+^**, which does not contain these hydroxy groups. It is noteworthy that a similar co‐conformation is often observed in the absence of a chloride anion, i.e. **4 ⋅ PF_6_** is relatively more preorganised for subsequent guest binding due to these organising interactions (Figure 6b).

**Figure 7:**
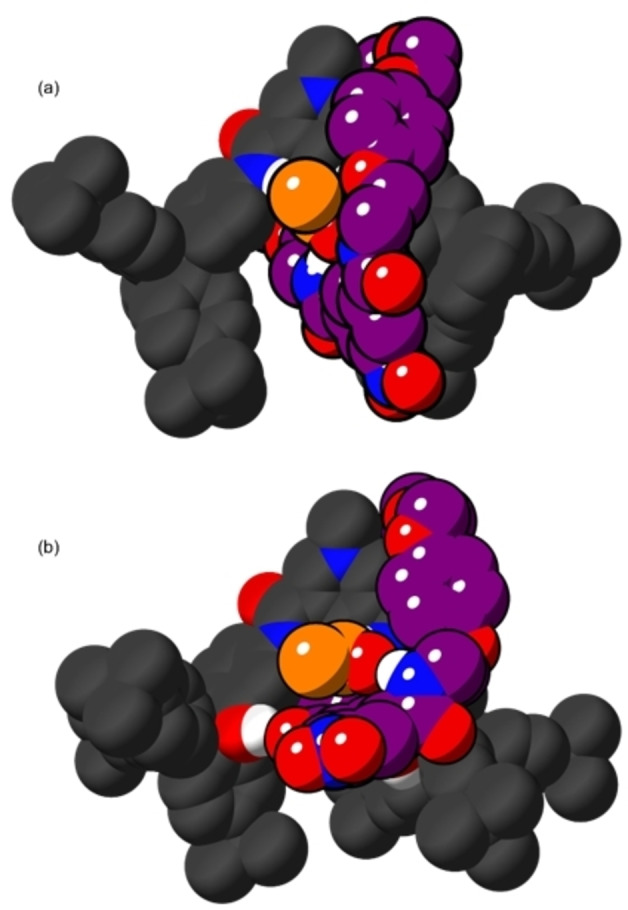
Representative structures from semiempirical simulations; (a) **1 ⋅ OAc**; (b) **4 ⋅ OAc**; most hydrogen atoms omitted for clarity, acetate anion carbon shown in orange.

When the acetate salts of the **1^+^** and **4^+^** were simulated, all four amide groups of **1^+^** hydrogen bonded to this anion slightly more frequently than in the simulations of **1 ⋅ Cl** (20 % vs. 7 % of the simulation time), consistent with this anion's high basicity, while this occurred less frequently with **4^+^** (6 % for **4 ⋅ OAc**, 1 % for **4 ⋅ Cl**). It is noteworthy that the oxoanion does not fit in the respective interlocked cavity and instead “perches” outside (Figure 7), as has previously been observed in molecular dynamics simulations of oxoanions binding to rotaxanes similar to **1^+^**.^[37]^ Again, both hydroxy groups of **4^+^** do not concomitantly participate in significant hydrogen bonding to the anion: with either a single hydroxy group binding to the anion outside of the binding pocket to the [2]rotaxane‘s exterior (41 % of the simulation time), or the hydroxy groups engaging in inter‐component interactions with the macrocycle carbonyl oxygens or the amide groups. In both cases, this appears to rigidify the binding pocket. We hypothesise that these inter‐component interactions act to hinder encapsulation of acetate and may explain the enhanced Cl^−^ vs. OAc^−^ selectivity observed for **4^+^**.

## Conclusion

In this work we demonstrate that the incorporation of additional phenolic hydrogen bond donors into an interlocked rotaxane host can significantly enhance anion binding selectivity. In particular, rotaxane **4 ⋅ PF_6_** containing a *meta*‐substituted bis‐phenolic O‐H axle component displays a much higher selectivity preference for chloride than an analogous rotaxane without the hydroxy groups. Computational studies indicated that this selectivity enhancement was due to the axle's hydroxy hydrogen bond donors pre‐organising the rotaxane's macrocycle, increasing the favourability of interactions between the host and the chloride anion. These studies also emphasised the dynamic and flexible nature of guest binding. This work clearly demonstrates the importance and influence of inter‐component preorganisation on guest binding affinity.^[38]^ We suggest that the strategic incorporation of groups designed to preorganise the components of interlocked hosts may lead to even stronger and more selective binding in similar systems.

## Experimental Section

**General remarks**: 3‐Aminophenyl acetate **7**,^[39]^ tris(*p*‐*tert*‐butylphenyl)methanol **8**,^[30]^ amino phenol stopper **9**,^[31]^ macrocycle precursor **14** ^[13]^ and [2]rotaxane **1 ⋅ PF_6_** ^[13]^ were prepared as previously described. Other compounds were bought from commercial suppliers and used as received. Details of instrumentation and characterization, as well as of X‐ray crystallography and semi‐empirical simulations are provided in the Supporting Information.

Deposition Number(s) 2150001 (for **4 ⋅ Cl**), 2150002 (for **15**), 2150003 (for **5 ⋅ Cl**) contain(s) the supplementary crystallographic data for this paper. These data are provided free of charge by the joint Cambridge Crystallographic Data Centre and Fachinformationszentrum Karlsruhe Access Structures service.

***Meta‐*** **hydroxy stopper 6**: Tris(*p*‐*tert*‐butylphenyl)methanol (0.32 g, 0.75 mmol, 1.0 mol eqiv.) was dissolved in toluene (0.75 mL). Acetyl chloride (0.23 mL, 2.6 mmol, 3.5 mol equiv.) was added dropwise and the solution subsequently heated to reflux for 30 minutes. After this time, the reaction was allowed to cool and hexane (10 mL) was added and the solution cooled in the freezer for 30 minutes resulting in the precipitation of a white solid. This was isolated by filtration and the resulting solid washed with cold hexane (5 mL) to afford white tris(4‐*tert*‐butylphenyl)methyl chloride. Once dried the tris(4‐*tert*‐butylphenyl)methyl chloride and *m*‐aminophenyl acetate **7** (0.218 g, 1.13 mmol, 1.5 mol equiv.) were added to a Schlenk tube and the solids were mixed thoroughly. The Schlenk tube was then submerged in an oil bath and heated to 160 °C for 3 h. After the 3 h the reaction mixture was cooled and washed with MeOH (20 mL). The resulting white solid was filtered and washed further with MeOH and dried *in vacuo*. Yield: 0.29 g, 70 %. **R**_***f***_: 0.62 (dichloromethane); ^**1**^ **H NMR (400 MHz, DMSO‐*d***_**6**_ **)** δ 8.45 (br. s, 1H), 7.21 (d, *J*=8.6 Hz, 6H), 7.01 (d, *J*=8.6 Hz, 6H), 6.57 (d, *J*=8.3 Hz, 1H), 5.96 ‐ 5.87 (m, 2H), 4.83 (br. s, 2H), 1.25 (s, 27H). ^**13**^ **C{^1^H} NMR (101 MHz, DMSO‐*d***_***6***_ **)**: δ 168.1, 155.5, 147.1, 143.0, 138.9, 130.1, 128.3, 123.7, 108.8, 107.4, 61.1, 34.0, 31.3; **HRMS (ESI^+^)**: 520.3579, calculated for [C_37_H_45_NO ⋅ H]^+^: 520.3579 Da.

***Meta‐*** **hydroxy axle 10**: Pyridine‐3,5‐dicarboxylic acid (0.030 g, 0.15 mmol, 0.50 mol equiv), EDC ⋅ HCl (0.070 g, 0.34 mmol, 1.1 mol equiv.) and stopper **6** (0.16 g, 0.31 mmol, 1.0 mol equiv.) were dissolved in DMF (5 mL). Triethylamine (0.060 g, 0.34 mmol, 1.1 mol equiv.) was added gradually and the resulting burgundy solution was stirred for 4 days under nitrogen. A saturated solution of NH_4_Cl_(aq)_ (10 mL) was then added to the reaction mixture, stirred briefly and then allowed to stand for 1 h. The resulting precipitate was filtered, washed with water (10 mL) and dried *in vacuo*. The solid was recrystallised from boiling diethyl ether (20 mL) and the resulting pale‐yellow powder was isolated by filtration, washed with cold diethyl ether (2×10 mL), and dried *in vacuo* (Yield: 0.10 g, 56 %). ^**1**^ **H NMR (400 MHz, DMSO‐*d***_**6**_ **)** δ 10.49 (s, 2H), 9.20 (s, 4H), 8.70 (s, 1H), 7.30 (s, 2H), 7.26 (d, *J*=8.4 Hz, 12H), 7.21 (d, *J*=8.4 Hz, 2H), 7.05 (d, *J*=8.4 Hz, 12H), 6.96 (d, *J*=8.4 Hz, 2H), 1.26 (s, 54H). ^**13**^ **C{^1^H} NMR (151 MHz, DMSO‐*d***_***6***_ **)**: δ 147.1, 142.9, 130.1, 123.7, 34.0, 31.2 (due to the limited solubility of this compound, we were unable to obtain satisfactory ^13^C data for this compound, and no further peaks could be detected). **HRMS (ESI^+^)**: 1192.6907, calculated for [C_81_H_91_N_3_O_4_ ⋅ Na]^+^: 1192.6907 Da.

***Ortho‐*** **hydroxy axle 11**: Pyridine‐3,5‐dicarboxylic acid (0.13 g, 0.79 mmol, 0.50 mol equiv.), EDC ⋅ HCl (0.33 g, 1.7 mmol, 1.1 mol equiv.) and stopper **9** (0.82 g, 1.6 mmol, 1.0 mol equiv.) were dissolved in DMF (5 mL). Triethylamine (0.18 g, 1.7 mmol, 1.1 mol equiv.) was added gradually and the resulting burgundy solution was stirred for 4 days under nitrogen. A saturated solution of NH_4_Cl_(aq)_ (10 mL) was then added to the reaction mixture, stirred briefly and then allowed to stand for 1 hr. The resulting precipitate was filtered, washed with water and dried. The solid was recrystallised from boiling diethyl ether (20 mL) and the resulting pale‐pink powder was isolated by filtration, washed with cold diethyl ether (2×10 mL), and dried *in vacuo*. Yield: 0.66 g, 72 %. ^**1**^ **H NMR (400 MHz, DMSO‐*d***_***6***_ **)**: δ 9.86 (br. s, 2H), 9.60 (br. s, 2H), 9.20 (br. s, 2H), 8.71 (br. s, 1H), 7.52 (d, *J*=8.3 Hz, 2H), 7.33 (d, *J*=8.4 Hz, 12H), 7.13 (d, *J*=8.4 Hz, 12H), 6.82 (s, 2H), 6.63 (d, *J*=8.3 Hz, 2H), 1.27 (s, 54H); ^**13**^ **C{^1^H} NMR (151 MHz, DMSO‐*d***_***6***_ **)**: δ 153.6, 147.8, 147.7, 144.1, 143.7, 130.0, 124.4, 122.8, 121.2, 118.3, 63.1, 34.1, 31.1 (due to the limited solubility of this compound, we were unable to obtain satisfactory ^13^C data for this compound, and no further peaks could be detected); **HRMS (ESI^+^)**: 1170.7087, calculated for [C_81_H_91_N_3_O_4_ ⋅ H]^+^: 1170.7082 Da.

***Meta‐*** **hydroxy axle 12 ⋅ BF_4_**: Neutral axle component **10** (0.042 g, 0.037 mmol, 1.0 mol equiv.) was dissolved in a solution of Me_3_OBF_4_ (0.008 g, 0.06 mmol, 1.5 mol equiv.) in dichloromethane (0.5 mL). This was stirred at room temperature under nitrogen for 24 h. Methanol (1.0 mL) was then added and the solution was concentrated under vacuum. The compound was dissolved in dichloromethane (5 mL) and washed with water (5 mL). The organic phase was dried over MgSO_4_ and concentrated under vacuum. The crude compound was purified by recrystallization in boiling diethyl ether (10 mL) and the resulting yellow powder was isolated by filtration, washed with cold diethyl ether (2×5 mL), and dried *in vacuo*. Yield: 0.038 g, 82 %. ^**1**^ **H NMR (400 MHz, CDCl_3_)** δ 10.84 (br. s, 2H), 10.30 (br. s, 1H), 8.86 (br. s, 2H), 7.30 (s, 2H), 7.25 (d, *J*=8.3 Hz, 2H), 7.19 (d, *J*=7.4 Hz, 12H), 7.05 (d, *J*=7.4 Hz, 12H), 6.92 (d, *J*=8.6 Hz, 2H), 3.80 (br. s, 3H), 1.21 (s, 54H). ^**13**^ **C{^1^H} NMR (151 MHz, CDCl_3_)**: δ 158.7, 154.9, 149.4, 148.0, 143.3, 141.4, 130.7, 124.9, 123.9, 112.6, 109.8, 106.3, 61.4, 55.5, 34.5, 31.5 (due to the limited solubility of this compound, we were unable to obtain satisfactory ^13^C data for this compound, and no further peaks could be detected); **HRMS (ESI^+^)**: 1184.7238, calculated for [C_82_H_94_N_3_O_4_]^+^: 1184.7238 Da.

***Ortho‐*** **hydroxy axle 13 ⋅ BF_4_**: Neutral axle component **12** (0.72 g, 0.62 mmol, 1.0 mol equiv.) was dissolved in a solution of Me_3_OBF_4_ (0.14 g, 0.93 mmol, 1.5 mol equiv.) in dichloromethane (1.0 mL). This was stirred at room temperature under nitrogen for 24 h. Methanol (10 mL) was then added and the solution was concentrated under vacuum. The compound was diluted with dichloromethane (10 mL) and washed with water (10 mL). The organic phases were then extracted, dried over MgSO_4_ and concentrated under vacuum. The crude compound was purified by recrystallization from boiling diethyl ether (10 mL) and the resulting yellow powder was isolated by filtration, washed with cold diethyl ether (2×5 mL), and dried *in vacuo*. Yield: 0.69 g, 87 %. ^**1**^ **H NMR (400 MHz, DMSO‐*d***_***6***_ **)**: δ 10.28 (br. s, 2H), 9.85 (br. s, 2H), 9.56 (s, 2H), 9.39 (s, 1H), 7.59 (d, *J*=8.5 Hz, 2H), 7.33 (d, *J*=8.4 Hz, 12H), 7.12 (d, *J*=8.4 Hz, 12H), 6.85 (s, 2H), 6.65 (d, *J*=8.4 Hz, 2H), 4.46 (s, 3H), 1.27 (s, 54H) ppm; ^**13**^ **C{^1^H} NMR (151 MHz, DMSO‐*d***_***6***_ **)**: 160.2, 148.8, 147.9, 147.3, 145.5, 143.7, 142.0, 133.3, 130.0, 124.5, 123.5, 122.3, 121.3, 118.2, 63.1, 48.5, 34.1, 31.2.; **HRMS (ESI^+^)**: 1184.7235, calculated for [C_82_H_94_N_3_O_4_]^+^: 1184.7238 Da.

***Meta‐*** **hydroxy rotaxane 4 ⋅ Cl**:^[33]^ Axle component **12 ⋅ BF_4_** (0.034 g, 0.027 mmol, 1.0 mol equiv.), TBA ⋅ Cl (0.01 g, 0.04 mmol, 1.5 mol equiv.) and bis‐vinyl macrocycle precursor **14** (0.077 g, 0.12 mmol, 4.4 mol equiv.) were dissolved in dichloromethane (10 mL). The solution was stirred for 30 minutes and then Grubb's II catalyst (0.016 g, 20 % w/w) was added and the solution stirred for 48 h under nitrogen. The solution was then concentrated under vacuum and purified by preparative TLC (ethyl acetate). Yield: 0.034 g, 68 %. ^**1**^ **H NMR (400 MHz, CDCl_3_)**: δ 10.19 (br. s, 2H), 9.78 (br. s, 1H), 9.29 (br. s, 1H), 9.13 (br. s, 2H), 8.93 (s, 2H), 8.68 (s, 2H), 7.63–7.57 (m, 2H), 7.41–7.37 (m, 2H), 7.28 (d, *J*=8.4 Hz, 12H), 7.01 (d, *J*=8.4 Hz, 12H), 6.91 (d, *J*=8.8 Hz, 2H), 6.47 (d, *J*=8.8 Hz, 4H), 6.20 (d, *J*=8.8 Hz, 4H), 6.11 (s, 2H), 4.74 (s, 2H), 4.41 (s, 3H), 4.13 (d, *J*=8.2 Hz, 8H), 3.82 ‐ 3.71 (m, 12H), 1.31 (s, 54H); ^**13**^ **C{^1^H} NMR (101 MHz, CDCl_3_)**: δ 164.7, 158.1, 155.2, 153.4, 152.1, 149.7, 145.6, 140.9, 137.5, 135.5, 134.0, 131.5, 131.3, 130.6, 126.5, 124.9, 115.0, 114.7, 113.1, 110.9, 71.2, 69.6, 68.3, 66.0, 61.3, 49.2 41.0, 34.5, 31.5; **HRMS (ESI^+^)**: 1805.9563, calculated for [C_114_H_129_N_6_O_14_]^+^: 1805.9564 Da.

***Meta‐*** **hydroxy rotaxane 4 ⋅ PF_6_**:^[33]^ **4 ⋅ Cl** (0.024 g, 0.013 mmol, 1.0 mol equiv.) was dissolved in dichloromethane (2 mL) and vigorously stirred with sat. NH_4_PF_6(aq)_ (5 mL) for 2 h. The mixture was extracted with dichloromethane (5 mL) the combined organic phase washed with sat. NH_4_PF_6(aq)_ (2×5 mL). It was then dried over MgSO_4_ and concentrated under vacuum to afford **4 ⋅ PF_6_** as yellow solid in a quantitative yield (0.025 g, 100 %). ^**1**^ **H NMR (400 MHz, CDCl_3_)**: δ 10.17 (br. s, 2H), 9.58 (br. s, 1H), 9.24 (br. s, 1H), 9.14 (br. s, 2H), 8.90 (s, 2H), 8.64 (br. s, 2H), 7.48 (s, 2H), 7.27 (d, *J*=8.6 Hz, 12H), 7.20 (d, *J*=8.8 Hz, 2H), 7.05 (d, *J*=8.6 Hz, 12H), 6.86–6.84(m, 2H), 6.50 (d, *J*=8.5 Hz, 4H), 6.30 (d, *J*=8.3 Hz, 4H), 6.08 (s, 2H), 4.40 (s, 3H), 4.11–4.07 (m, 4H), 3.98 ‐ 3.97 (m, 4H), 3.79–3.76 (m, 8H), 3.66–3.64 (m, 4H), 1.30 (s, 54H); ^**31**^ **P{^1^H} NMR (162 MHz, CDCl_3_)**: δ −144.2 (sep, *J*=711 Hz) ppm; ^**19**^ **F{^1^H} NMR (377 MHz, CDCl_3_)**: δ −72.2 (d, *J*=711 Hz) ppm; **HRMS (ESI^+^)**: 1805.9566, calculated for [C_114_H_129_N_6_O_14_]^+^: 1805.9564 Da.

***Ortho‐*** **hydroxy rotaxane 5 ⋅ Cl**:^[33]^ Axle component **13 ⋅ BF_4_** (0.030 g, 0.021 mmol, 1.0 mol equiv.), TBA ⋅ Cl (0.01 g, 0.04 mmol, 1.5 mol equiv.) and bis‐vinyl macrocycle precursor **14** (0.081 g, 0.13 mmol, 6.0 mol equiv.) were dissolved in dichloromethane (10 mL). This was stirred for 30 minutes and then Grubb's II catalyst (0.016 g, 20 % w/w) was added and the solution stirred for 48 h under nitrogen. The solution was then concentrated under vacuum and purified by preparative TLC (4 : 1 ethyl acetate:petroleum spirits 60–80 °C). Yield: 0.023 g, 51 %. ^**1**^ **H NMR (400 MHz, CDCl_3_)**: δ 10.41 (br. s, 2H), 9.85 (br. s, 1H), 9.21 (br. s, 2H), 9.11 (br. s, 1H), 8.94 (br. s, 2H), 8.42 (s, 2H), 7.95 (s, 2H), 7.51 (d, *J*=8.0 Hz, 2H), 7.23 (d, *J*=8.3 Hz, 12H), 7.02 (d, *J*=8.0 Hz, 12H), 6.82 (s, 2H), 6.69 (d, *J*=7.9 Hz, 2H), 6.52 (d, *J*=8.6 Hz, 4H), 6.21 (d, *J*=8.6 Hz, 4H), 6.04 (s, 2H), 4.46 (s, 3H), 4.10 (d, *J*=22.4 Hz, 8H), 3.78 (d, *J*=22.5 Hz, 12H), 1.31 (s, 54H); ^**13**^ **C{^1^H} NMR (101 MHz, CDCl_3_)**: δ 173.0, 164.7, 159.1, 152.2, 149.0, 148.8, 148.7, 148.5, 143.5, 130.7, 130.5, 130.2, 126.7, 124.8, 124.4, 122.1, 115.3, 114.9, 71.1, 69.6, 68.3, 66.3, 63.6, 49.2, 41.2, 34.6, 31.5 (due to the limited solubility of this compound, we were unable to obtain satisfactory ^13^C data for this compound, and no further peaks could be detected); **HRMS (ESI^+^)**: 1805.9556, calculated for [C_114_H_129_N_6_O_14_ ⋅ H]^+^: 1805.9564 Da.

***Ortho‐*** **hydroxy rotaxane 5 ⋅ PF_6_**:^[33]^ **5 ⋅ Cl** (0.020 g, 0.0080 mmol, 1.0 mol equiv.) was dissolved in dichloromethane (2 mL) and vigorously stirred with sat. NH_4_PF_6(aq)_ (5 mL) for 2 h. The mixture was extracted with dichloromethane (5 mL) and the combined organic phase washed with sat. NH_4_PF_6(aq)_ (2×5 mL). It was then dried over MgSO_4_ and concentrated under vacuum to afford **5 ⋅ PF_6_** as yellow solid in a quantitative yield (0.016 g, 100 %). ^**1**^ **H NMR (400 MHz, CDCl_3_)**: δ 10.36 (br. s, 2H), 9.68 (br. s, 1H), 9.17 (br. s, 2H), 9.07 (br. s, 1H), 8.95 (s, 2H), 8.37 (br. s, 2H), 7.41 (s, 2H), 7.23 (d, *J*=8.6 Hz, 12H), 7.08–7.06 (m, 2H), 7.04 (d, *J*=8.6 Hz, 12H), 6.85–6.82 (m, 2H), 6.53 (d, *J*=8.8 Hz, 4H), 6.27 (d, *J*=8.8 Hz, 4H), 5.98 (s, 2H), 4.40 (br. s, 3H), 4.02 (br. s, 8H), 3.75–3.72 (m, 12H), 1.30 (s, 54H); ^**31**^ **P{^1^H} NMR (162 MHz, CDCl_3_)**: δ −144.2 (sep, *J*=711 Hz) ppm; ^**19**^ **F{^1^H} NMR (377 MHz, CDCl_3_)**: δ −72.2 (d, *J*=711 Hz) ppm; **HRMS (ESI^+^)**: 1805.9564, calculated for [C_114_H_129_N_6_O_14_]^+^: 1805.9564 Da.

## Conflict of interest

The authors declare no conflict of interest.

1

## Supporting information

As a service to our authors and readers, this journal provides supporting information supplied by the authors. Such materials are peer reviewed and may be re‐organized for online delivery, but are not copy‐edited or typeset. Technical support issues arising from supporting information (other than missing files) should be addressed to the authors.

Supporting Information

## Data Availability

The data that support the findings of this study are available in the supplementary material of this article.
