# Supplementary material for: Hydroxy Groups Enhance [2]Rotaxane Anion Binding Selectivity
Source: Chemistry. 2022 Apr 5;28(28):e202200389. doi: 10.1002/chem.202200389 (PMC9321576; doi:10.1002/chem.202200389)
Supplement: Supplementary file 1 — Supporting Information [file CHEM-28-0-s001.pdf]

Chemistry–A European Journal

Supporting Information

Hydroxy Groups Enhance [2]Rotaxane Anion Binding Selectivity

Rosemary J. Goodwin, Andrew Docker, Hugo I. MacDermott-Opeskin, Heather M. Aitken, Megan L. O’Mara, Paul D. Beer, and Nicholas G. White*

Synthesis and characterization **2**
General remarks 2
Characterization data 3
Difficulties encountered in the synthesis of **15** 15

Quantitative anion binding studies **16**
General protocol 16
Anion binding data, and NMR spectra and isotherms 17

Single crystal X-ray diffraction data **32**
Data collection 32
Data refinement 32
Table of crystallographic data 36

Computational simulations **37**
General information 37
CREST and simulation data analysis 38
Starting geometries 46
Time trace graphs 51

References **84**

Synthesis and characterization

General remarks

3-Aminophenyl acetate **7**,[1] tris(*p*-*tert*-butylphenyl)methanol **8**,[2] amino phenol stopper **9**,[3] macrocycle precursor **14**[4] and [2]rotaxane **1·PF$_6$**[4] were prepared as previously described. Other compounds were bought from commercial suppliers and used as received. NMR spectra were collected on Bruker Avance 400 or Bruker Avance 600 spectrometers and are referenced to the residual solvent signal.[5] Electrospray ionisation mass spectrometry data were acquired on a Micromass Waters ZMD spectrometer.

Quantitative ^{19}F{^{1}H} NMR was used to determine complete conversion of **4·Cl** and **5·Cl** to **4·PF$_6$** and **5·PF$_6$**, respectively.[6,7] The experiments were conducted at 298 K, with a 20 second delay time to ensure complete relaxation of the fluorine nuclei. Solutions of **4·Cl** and **5·Cl** were prepared (2.0 mmol L^{-1} of rotaxane and 1.0 mmol L^{-1} of 2,2,2-trifluoroethanol), with a peak integration ratio of 2:0.5 indicating complete conversion.

Characterization data

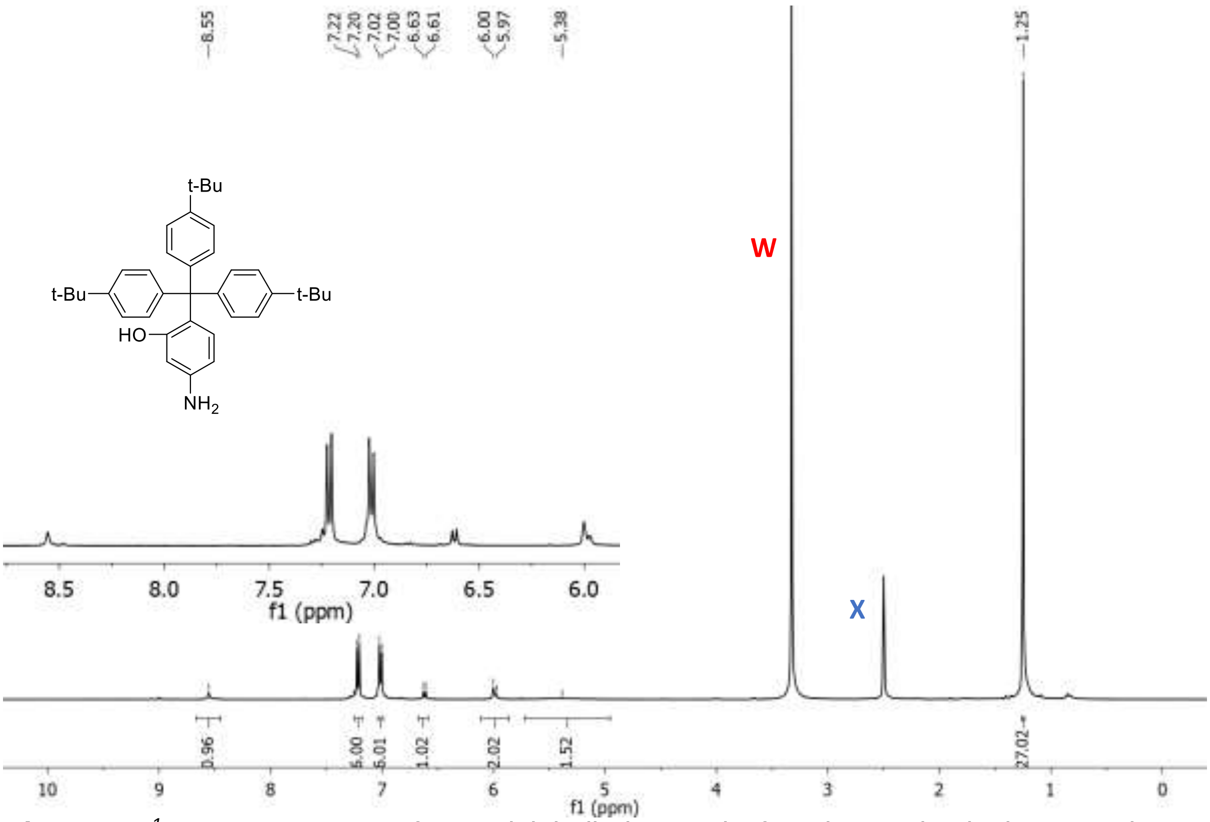

Figure S1. *^{1}H NMR spectrum of **6**; peak labelled **X** results from incompletely deuterated NMR solvent, residual water peak marked by **W**, (400 MHz, DMSO-d_6).*

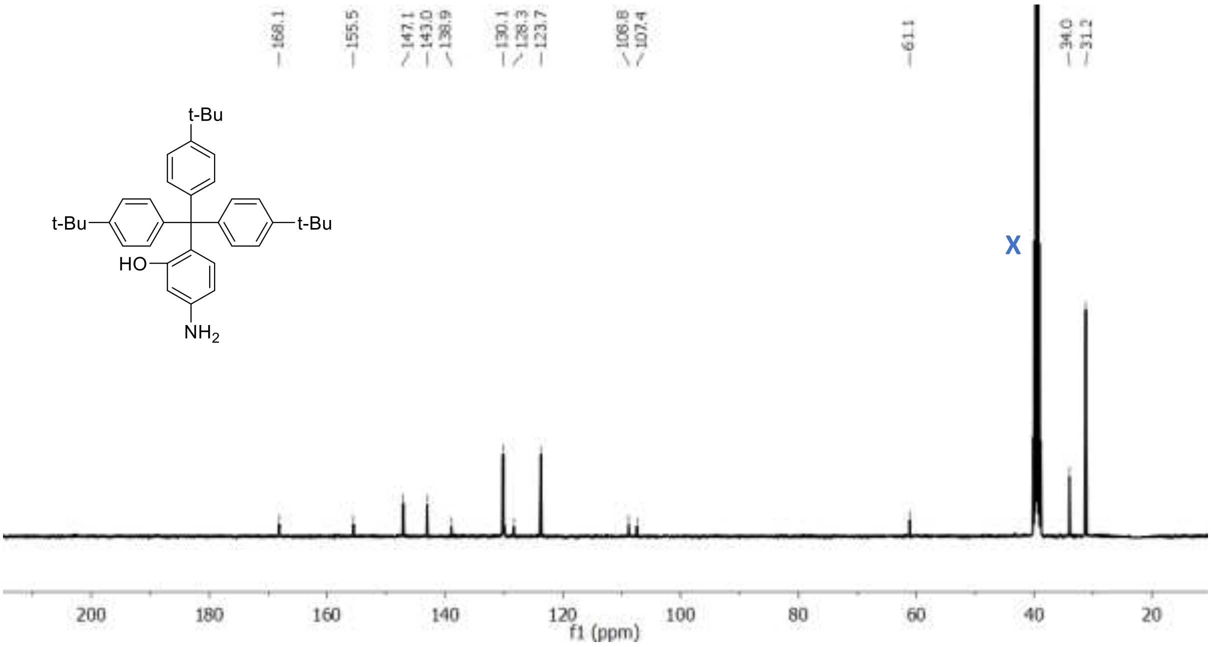

Figure S2. *^{13}C{^{1}H} NMR spectrum of **6**; peak labelled **X** results from incompletely deuterated NMR solvent (101 MHz, DMSO-d_6).*

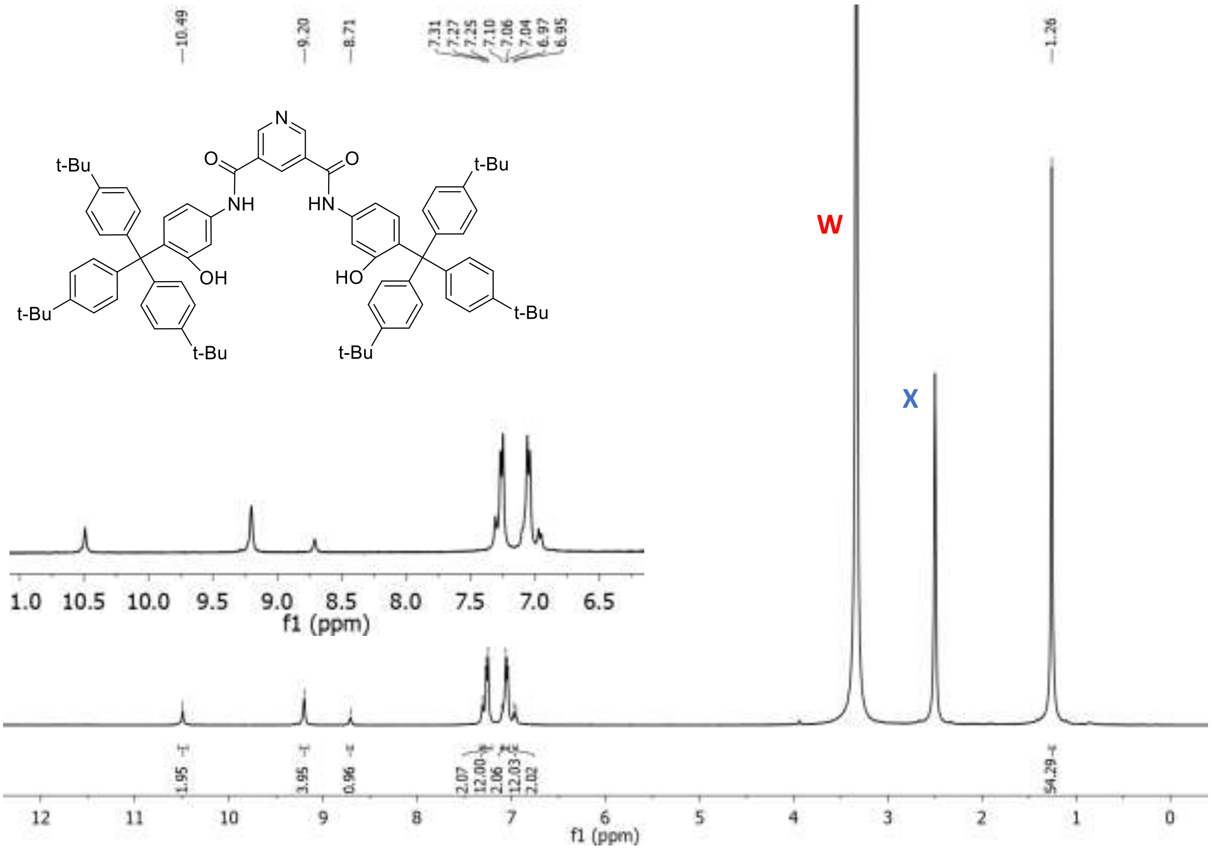

Figure S3. *^{1}H NMR spectrum of **10**; peak labelled **X** results from incompletely deuterated NMR solvent, residual water peak marked by **W**, (400 MHz, DMSO-d_6).*

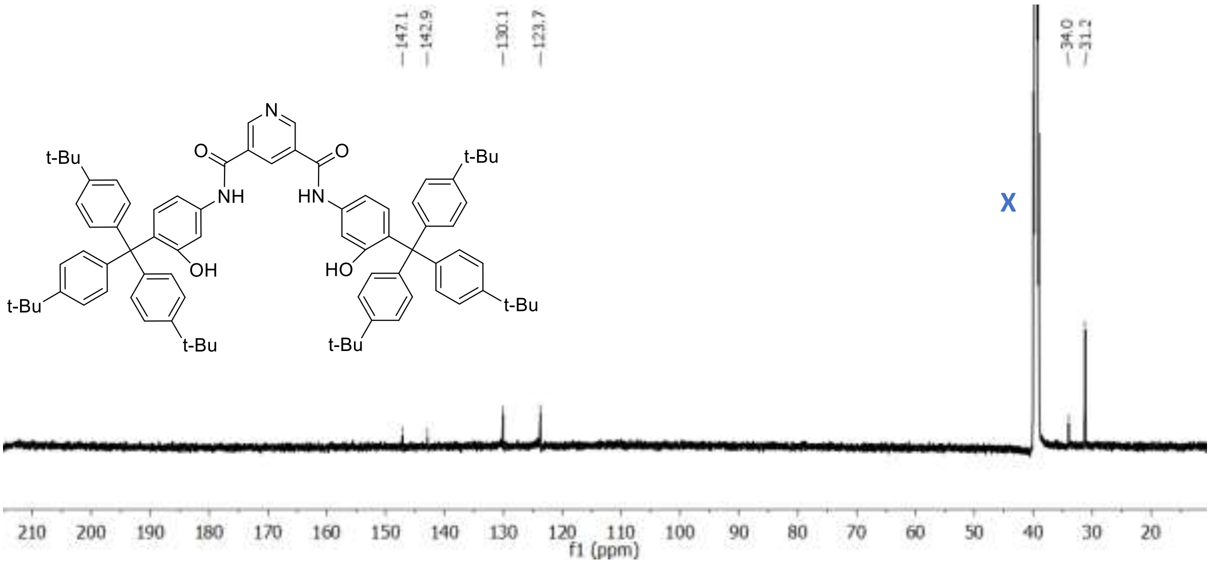

Figure S4. *^{13}C{^{1}H} NMR spectrum of **10**; peak labelled **X** results from incompletely deuterated NMR solvent (151 MHz, DMSO-d_6). Due to the limited solubility of this compound, we were unable to obtain satisfactory ^{13}C data for this compound, and no further peaks could be detected.*

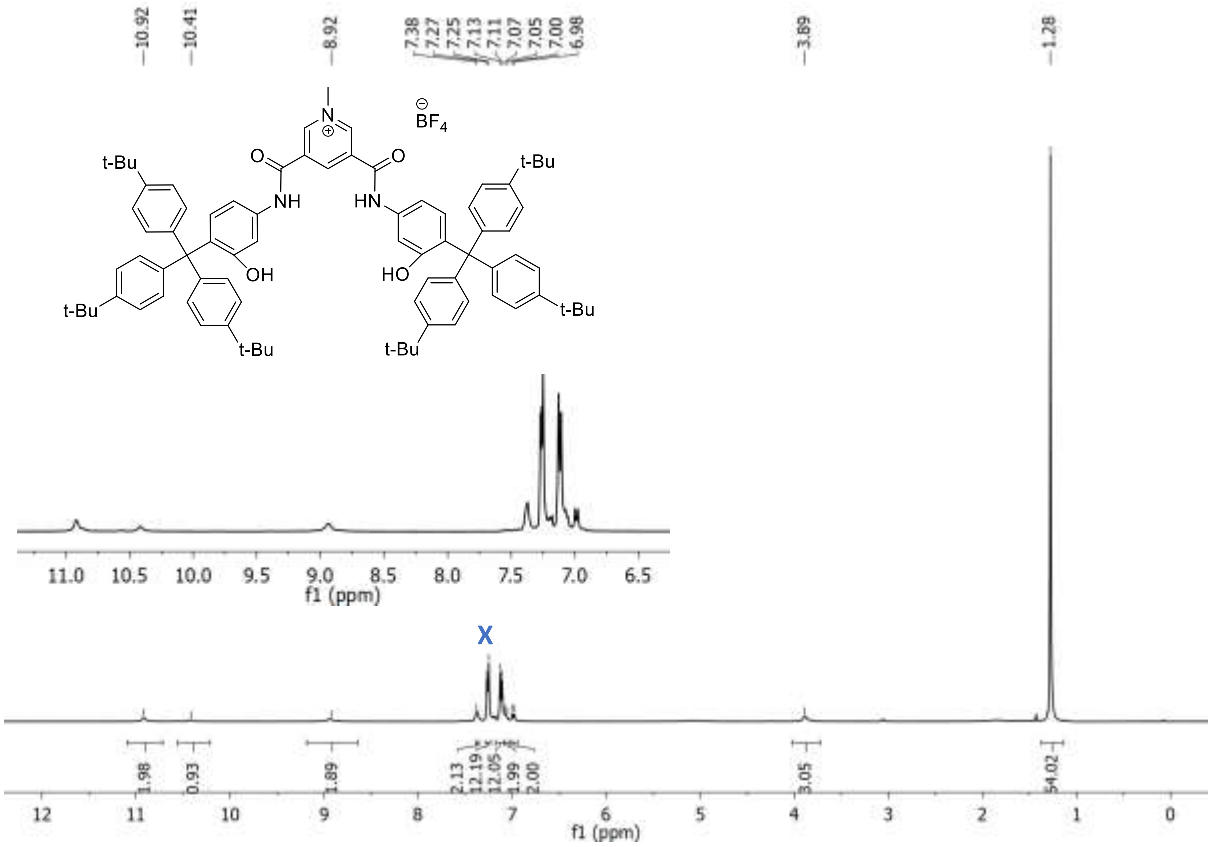

Figure S5. *^{1}H NMR spectrum of* ***12·BF₄****; peak labelled* **X** *results from incompletely deuterated NMR solvent (400 MHz, $CDCl_3$).*

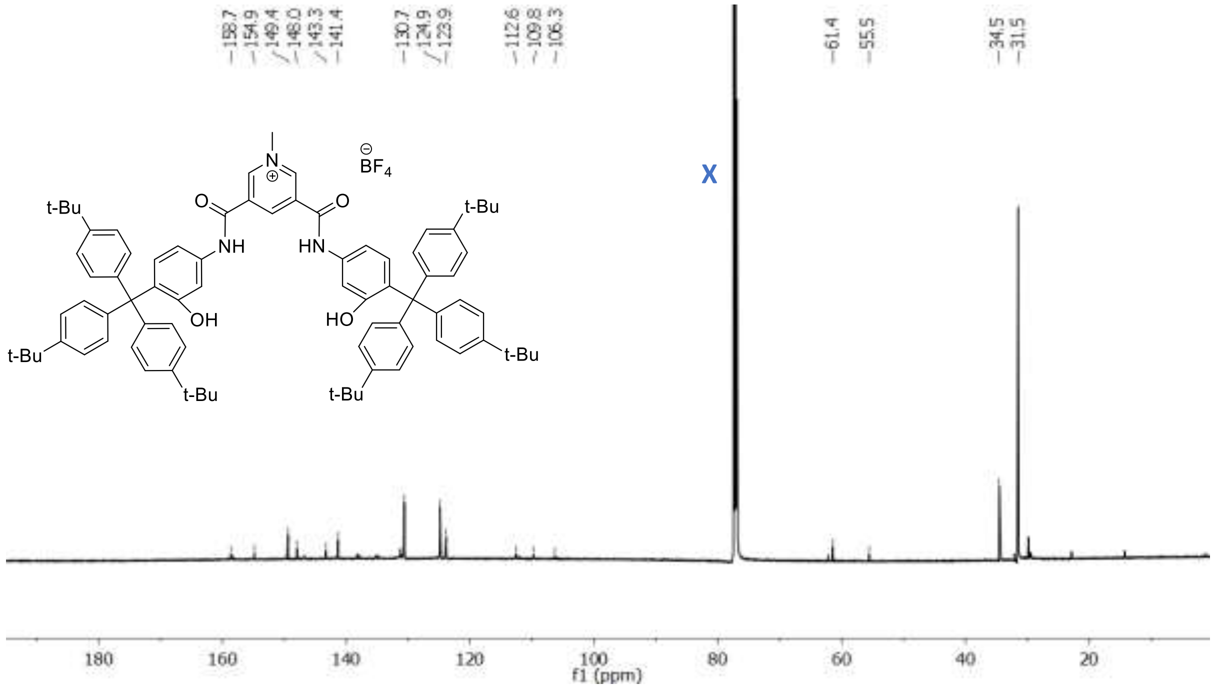

Figure S6. *^{13}C{^{1}H} NMR spectrum of* ***12·BF₄****; peak labelled* **X** *results from incompletely deuterated NMR solvent (101 MHz, $CDCl_3$). Due to the limited solubility of this compound, we were unable to obtain satisfactory ^{13}C data for this compound, and no further peaks could be detected.*

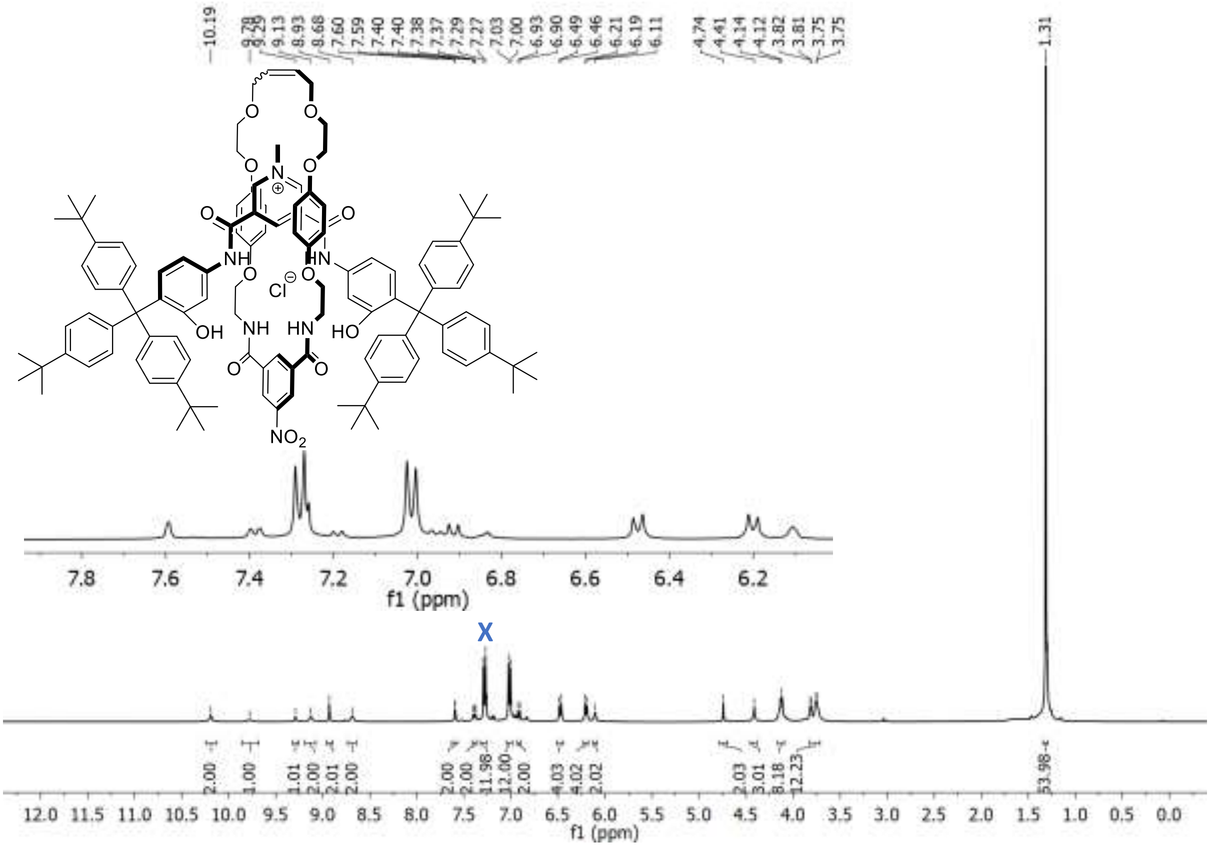

Figure S7. *^{1}H NMR spectrum of **4·Cl**; peak labelled **X** results from incompletely deuterated NMR solvent (400 MHz, $CDCl_3$).*

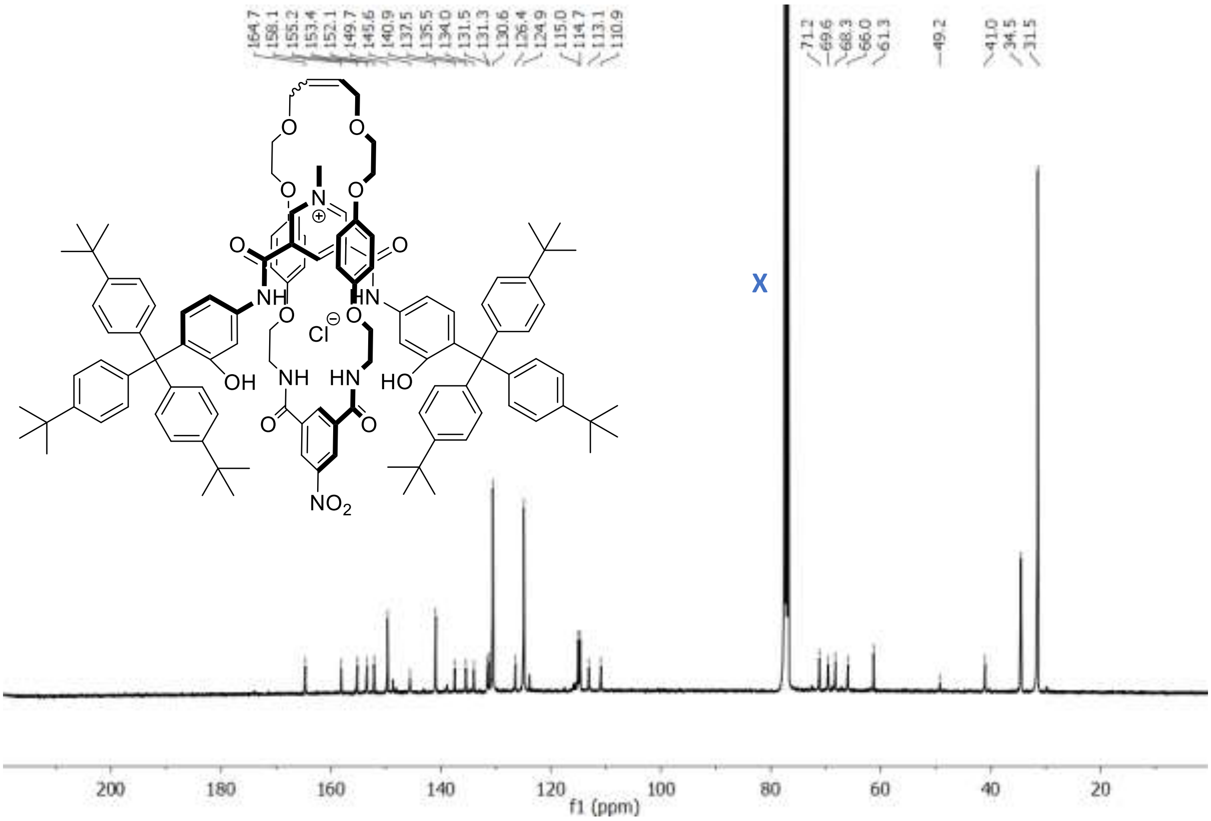

Figure S8. *^{13}C{^{1}H} NMR spectrum of **4·Cl**; peak labelled **X** results from incompletely deuterated NMR solvent (101 MHz, $CDCl_3$).*

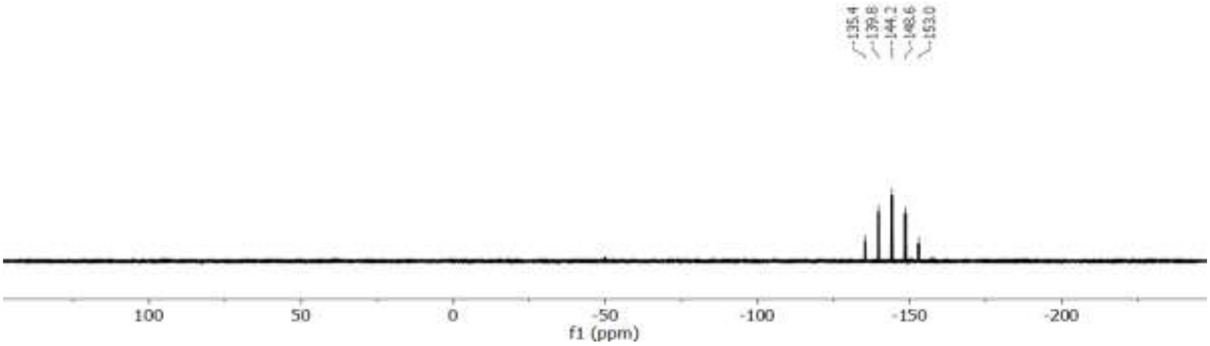

Figure S9. *$^{31}P\{^{1}H\}$ NMR spectrum of **4·PF$_6$** (162 MHz, $CDCl_3$).*

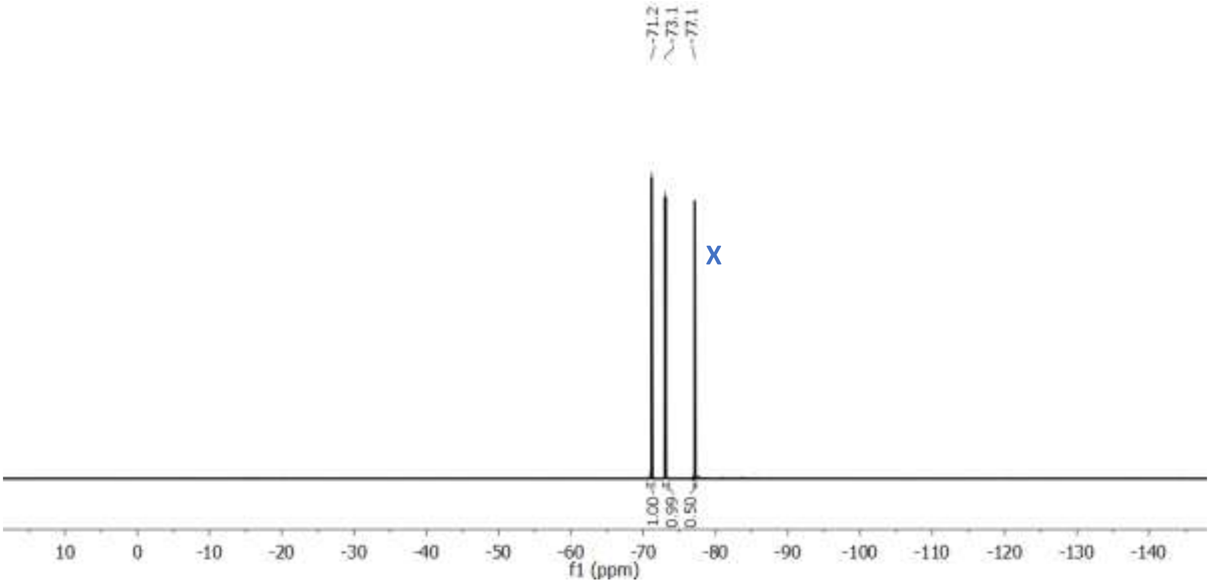

Figure S10. *Quantitative $^{19}F\{^{1}H\}$ NMR spectrum of **4·PF$_6$**; peak labelled **X** results from 2,2,2-trifluoroethanol standard (377 MHz, $CDCl_3$).*

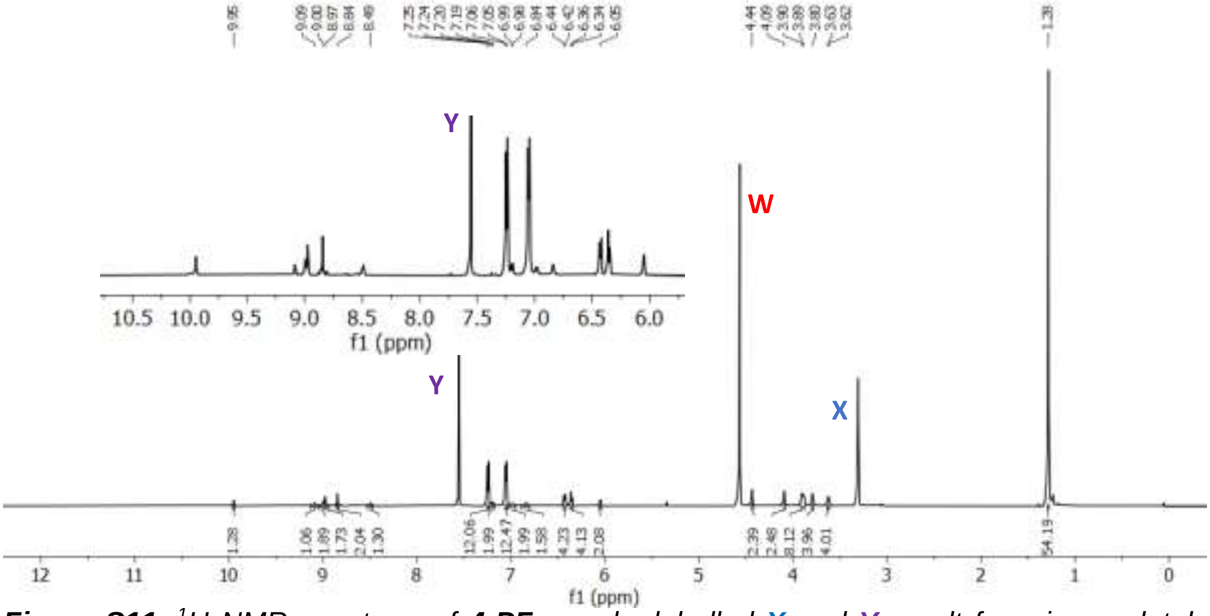

Figure S11. *^{1}H NMR spectrum of **4·PF$_6$**; peaks labelled **X** and **Y** result from incompletely deuterated NMR solvents MeOD and $CDCl_3$ respectively, residual water peak marked by **W**, (600 MHz, 1:1 MeOD:$CDCl_3$).*

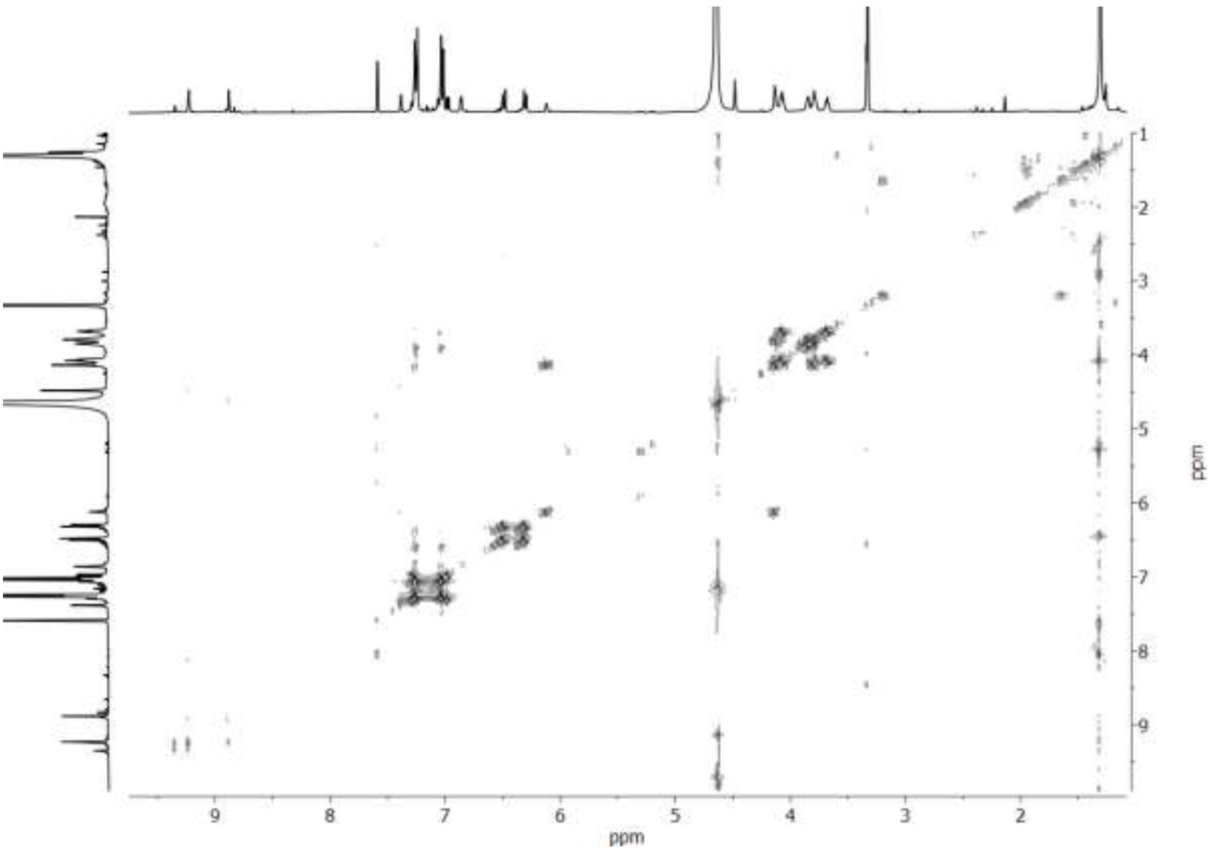

Figure S12. *COSY NMR spectrum of **4·PF$_6$** (400 MHz, 1:1 MeOD:CDCl$_3$).*

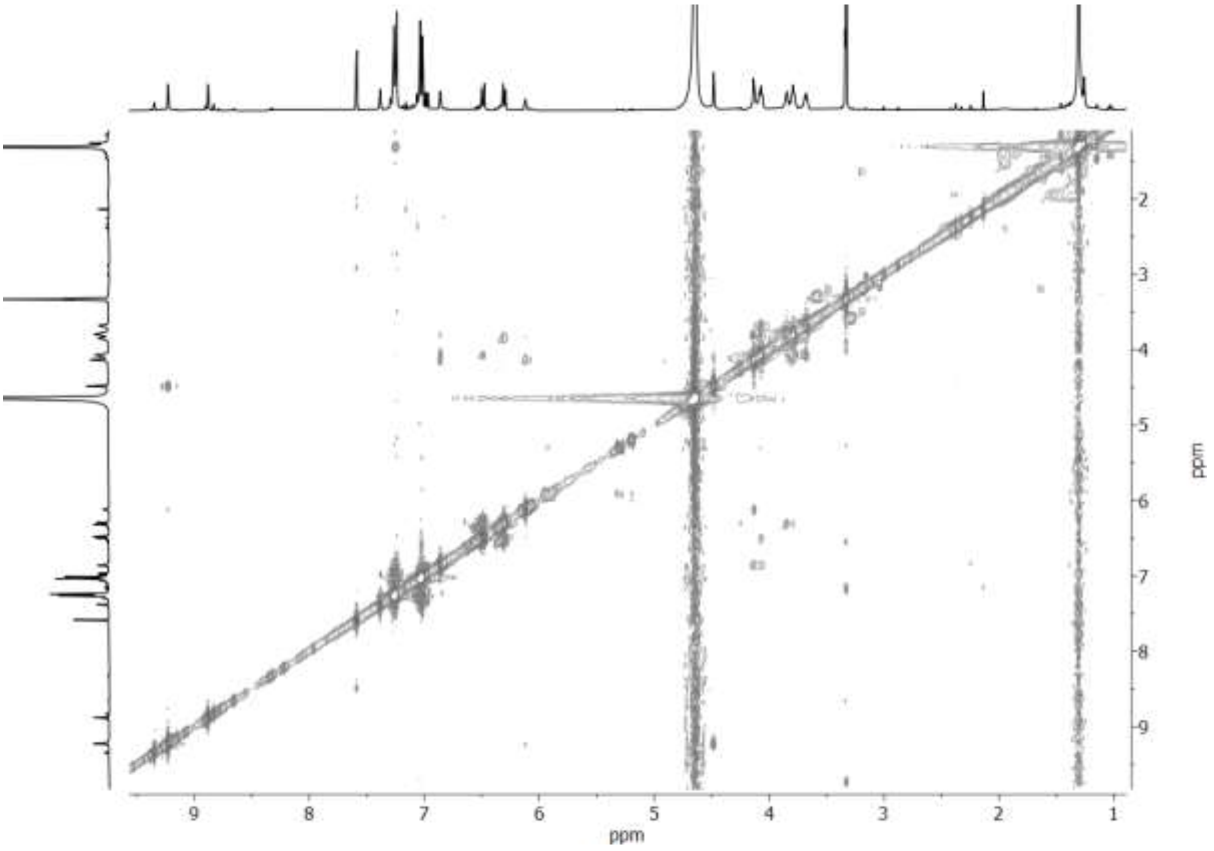

Figure S13. *NOESY NMR spectrum of **4·PF$_6$** (400 MHz, 1:1 MeOD:CDCl$_3$).*

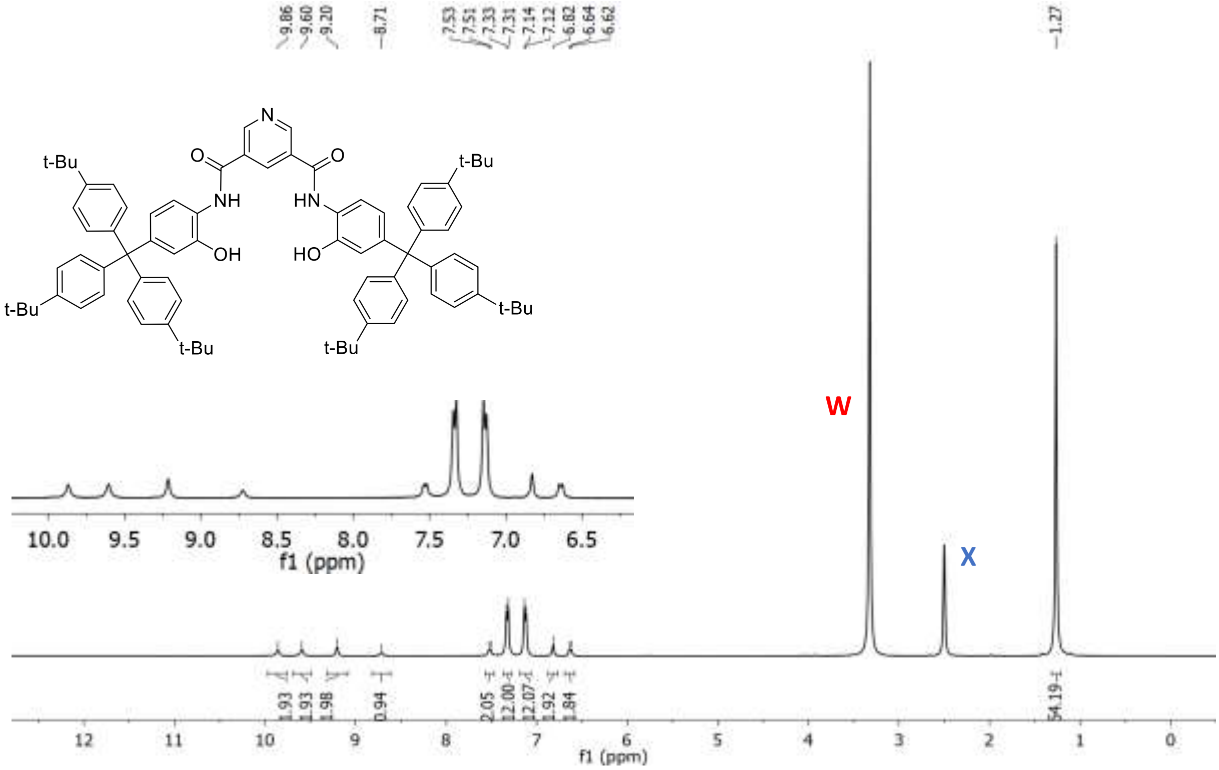

Figure S14. *^{1}H NMR spectrum of **11**; peak labelled X results from incompletely deuterated NMR solvent, residual water peak marked by W, (400 MHz, DMSO-d_6).*

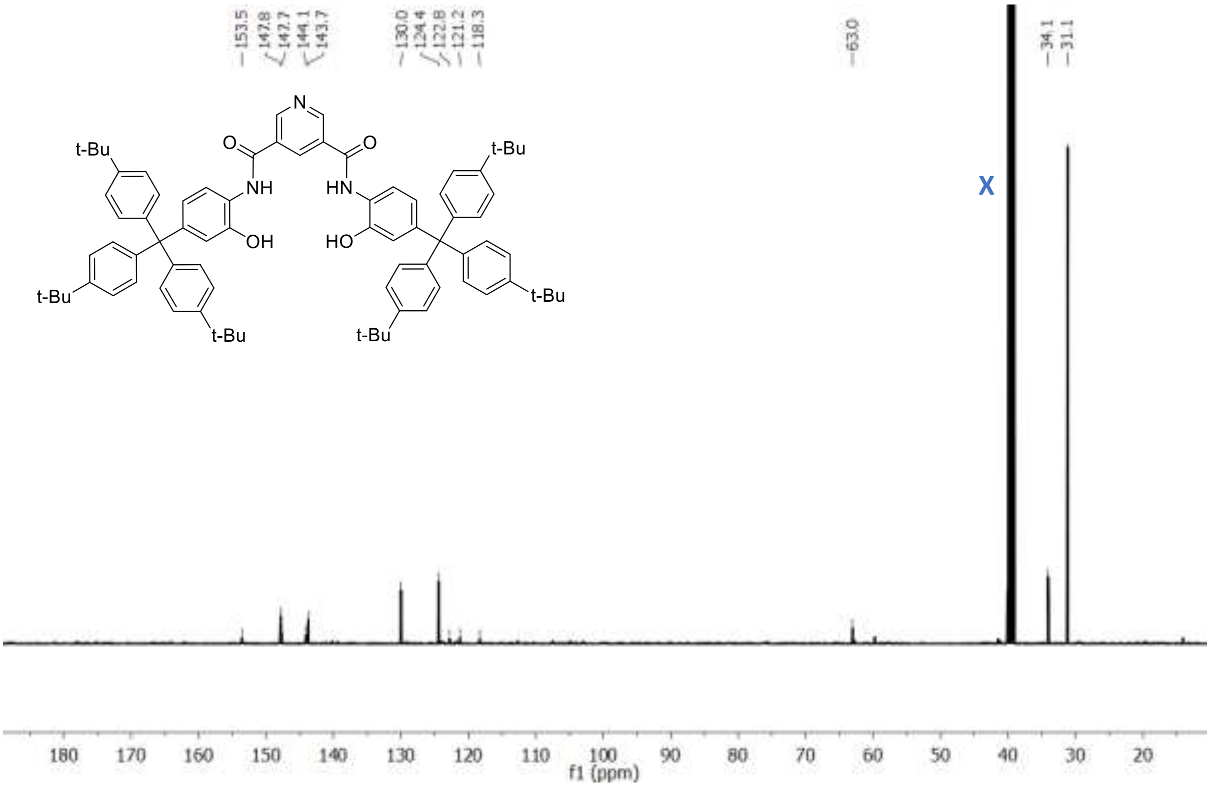

Figure S15. *^{13}C{^{1}H} NMR spectrum of **11**; peak labelled X results from incompletely deuterated NMR solvent (151 MHz, DMSO-d_6). Due to the limited solubility of this compound, we were unable to obtain satisfactory ^{13}C data for this compound, and no further peaks could be detected.*

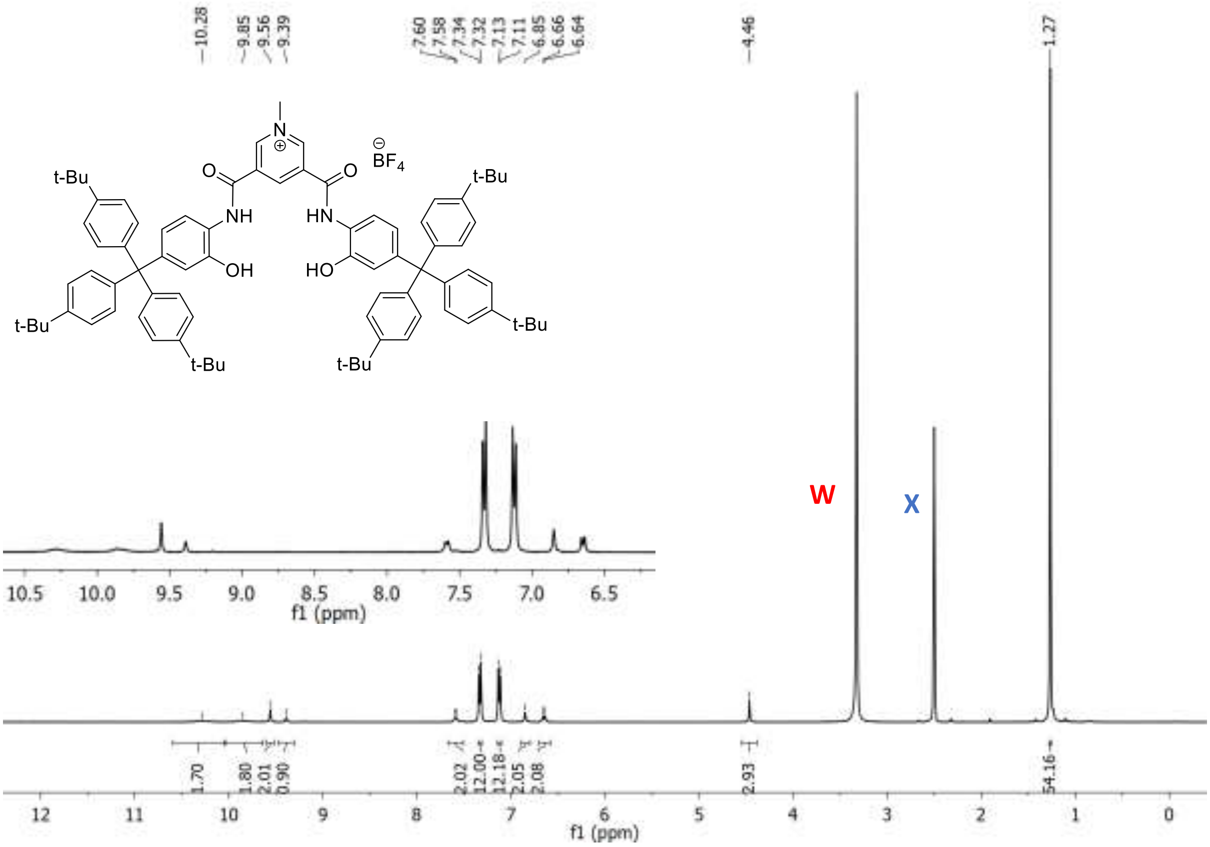

Figure S16. ^{1}*H NMR spectrum of* ***13·BF***$_{4}$*; peak labelled* ***X*** *results from incompletely deuterated NMR solvent, residual water peak marked by* ***W****, (400 MHz, DMSO-d*$_{6}$*).*

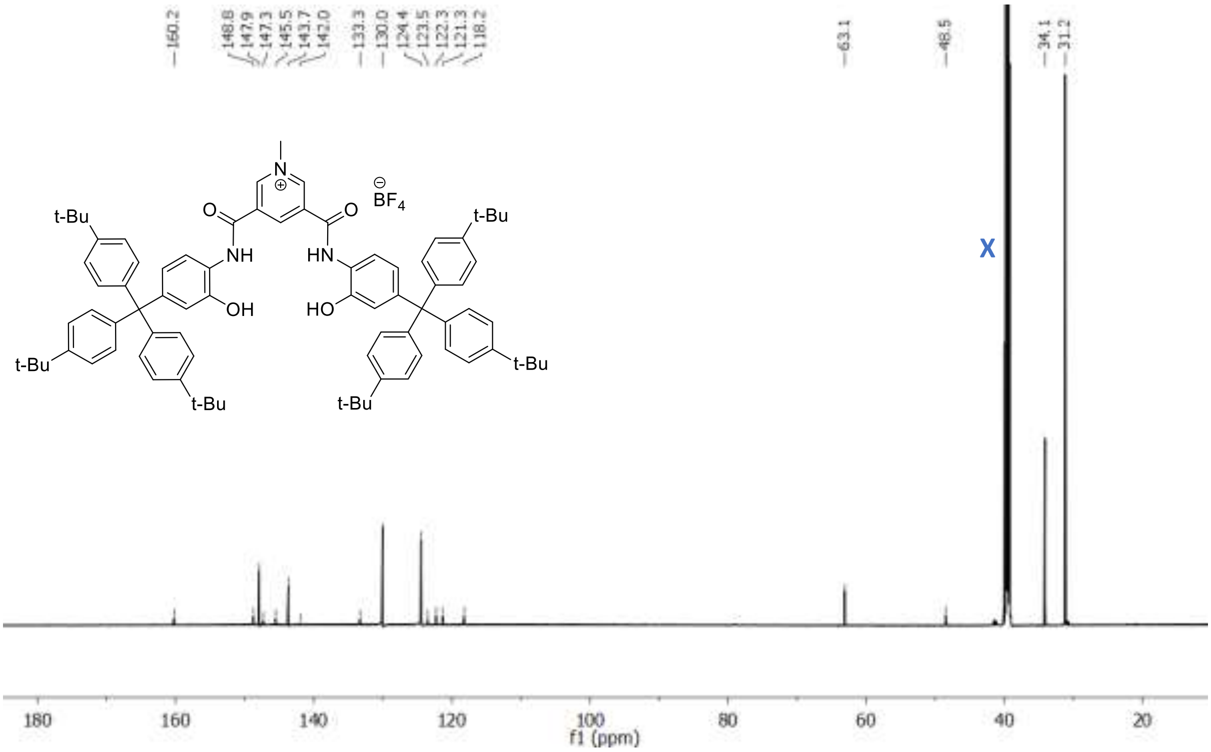

Figure S17. ^{13}*C{*^{1}*H} NMR spectrum of* ***13·BF***$_{4}$*; peak labelled* ***X*** *results from incompletely deuterated NMR solvent (151 MHz, DMSO-d*$_{6}$*).*

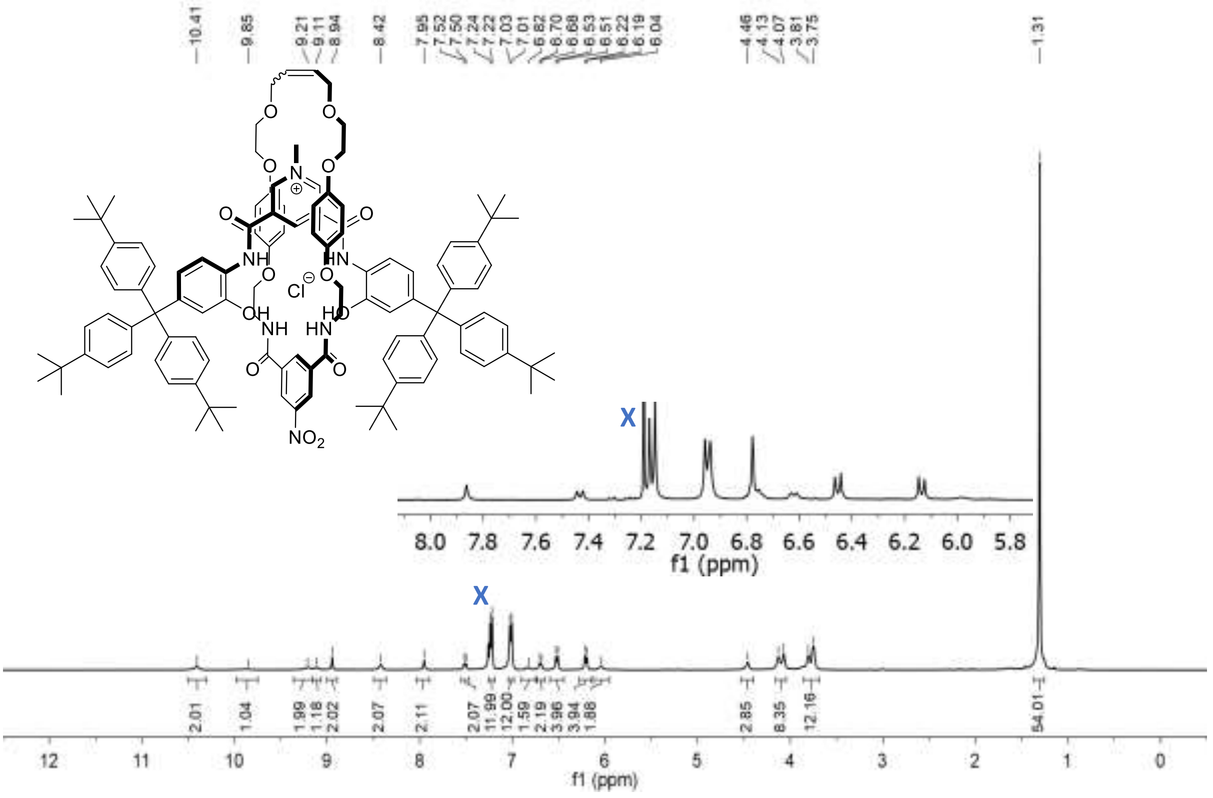

Figure S18. *^{1}H NMR spectrum of **5·Cl**; peak labelled **X** results from incompletely deuterated NMR solvent (400 MHz, $CDCl_3$).*

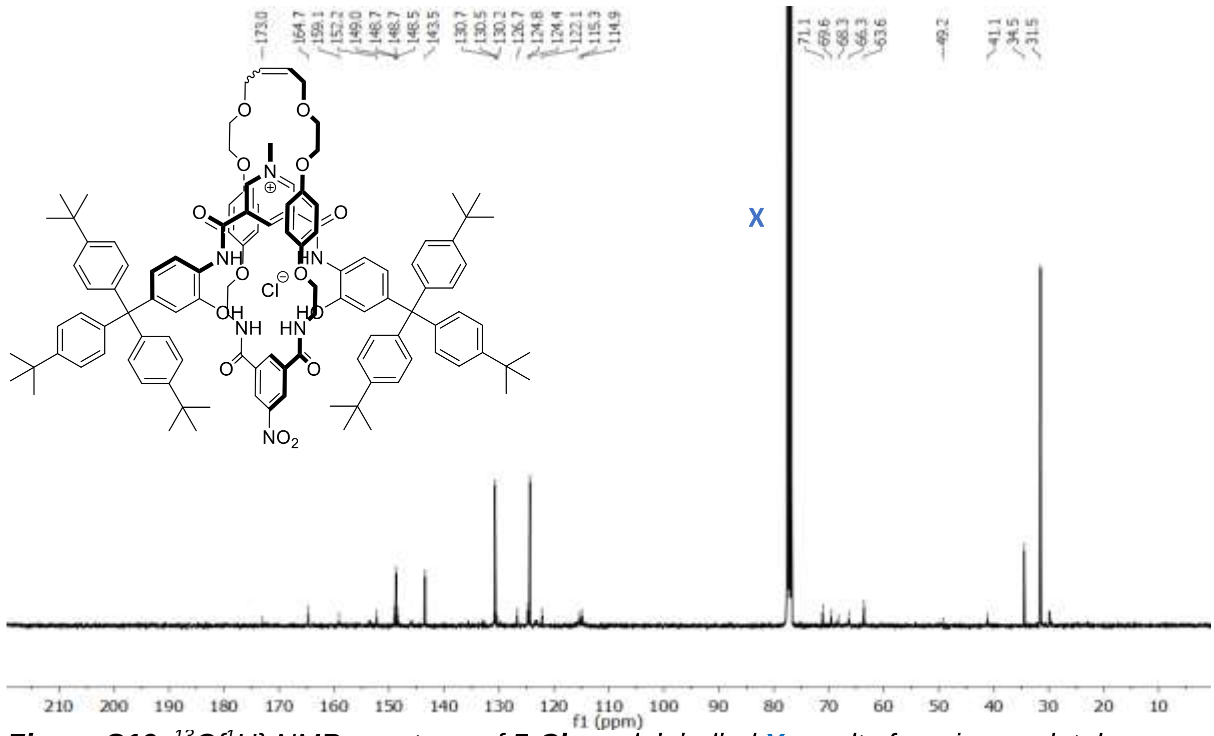

Figure S19. *^{13}C{^{1}H} NMR spectrum of **5·Cl**; peak labelled **X** results from incompletely deuterated NMR solvent (101 MHz, $CDCl_3$). Due to the limited solubility of this compound, we were unable to obtain satisfactory ^{13}C data for this compound, and no further peaks could be detected.*

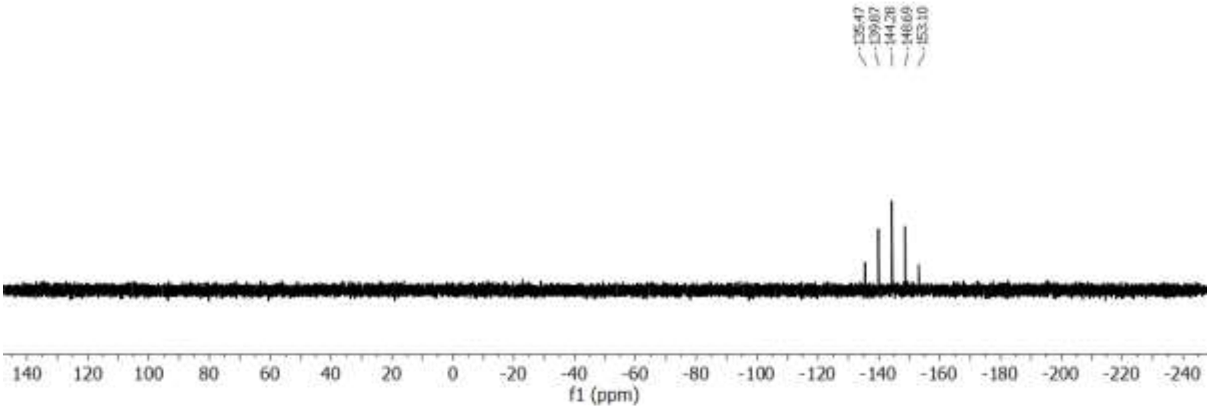

Figure S20. *$^{31}P\{^{1}H\}$ NMR spectrum of **5·PF$_6$** (162 MHz, $CDCl_3$)*

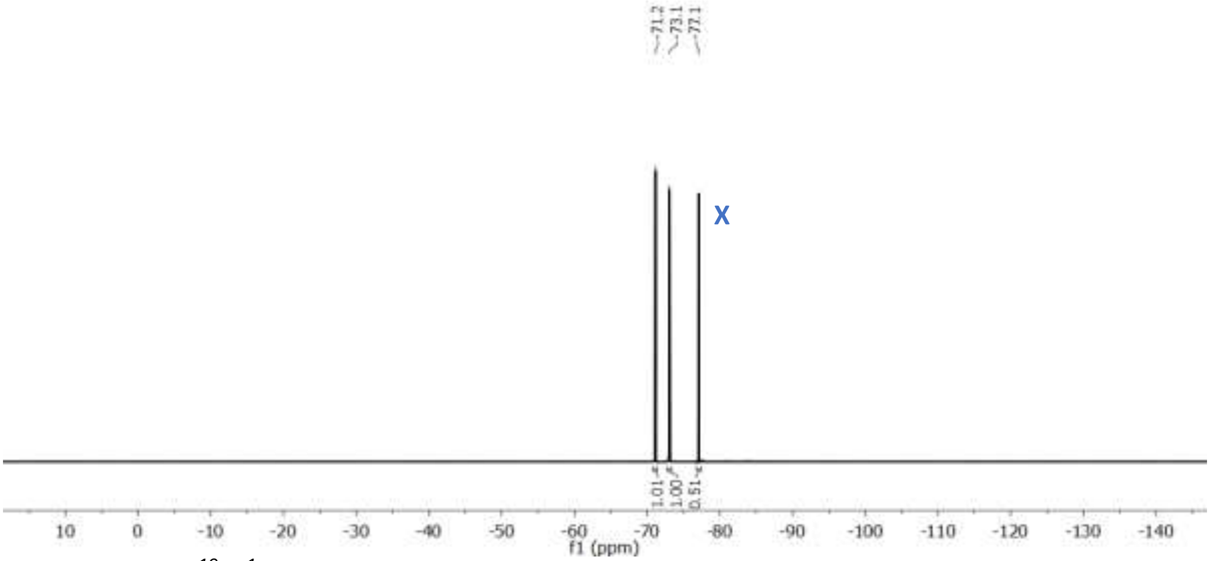

Figure S21. *$^{19}F\{^{1}H\}$ NMR spectrum of **5·PF$_6$**; peak labelled **X** results from 2,2,2-trifluoroethanol standard (377 MHz, $CDCl_3$).*

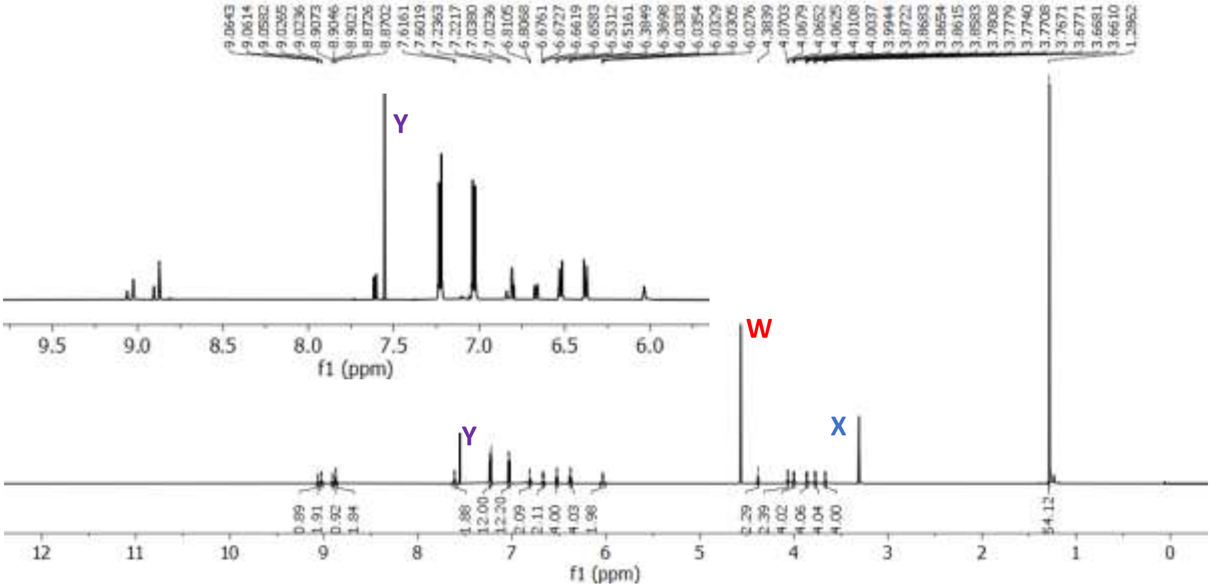

Figure S22. *^{1}H NMR spectrum of **5·PF$_6$**; peaks labelled **X** and **Y** result from incompletely deuterated NMR solvents MeOD and $CDCl_3$ respectively, residual water peak marked by **W**, (600 MHz, 1:1 MeOD:$CDCl_3$).*

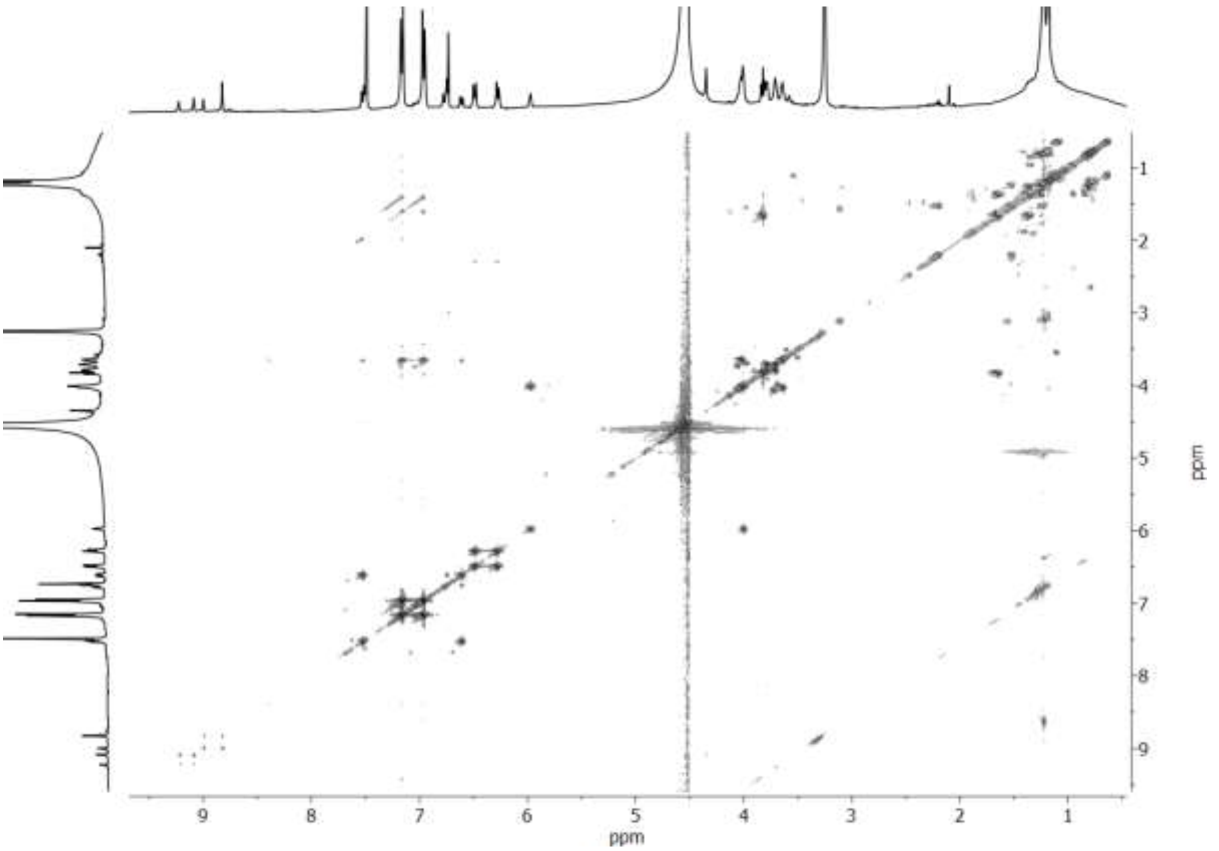

Figure S23. *COSY NMR spectrum of **5·PF₆** (600 MHz, 1:1 MeOD:CDCl₃).*

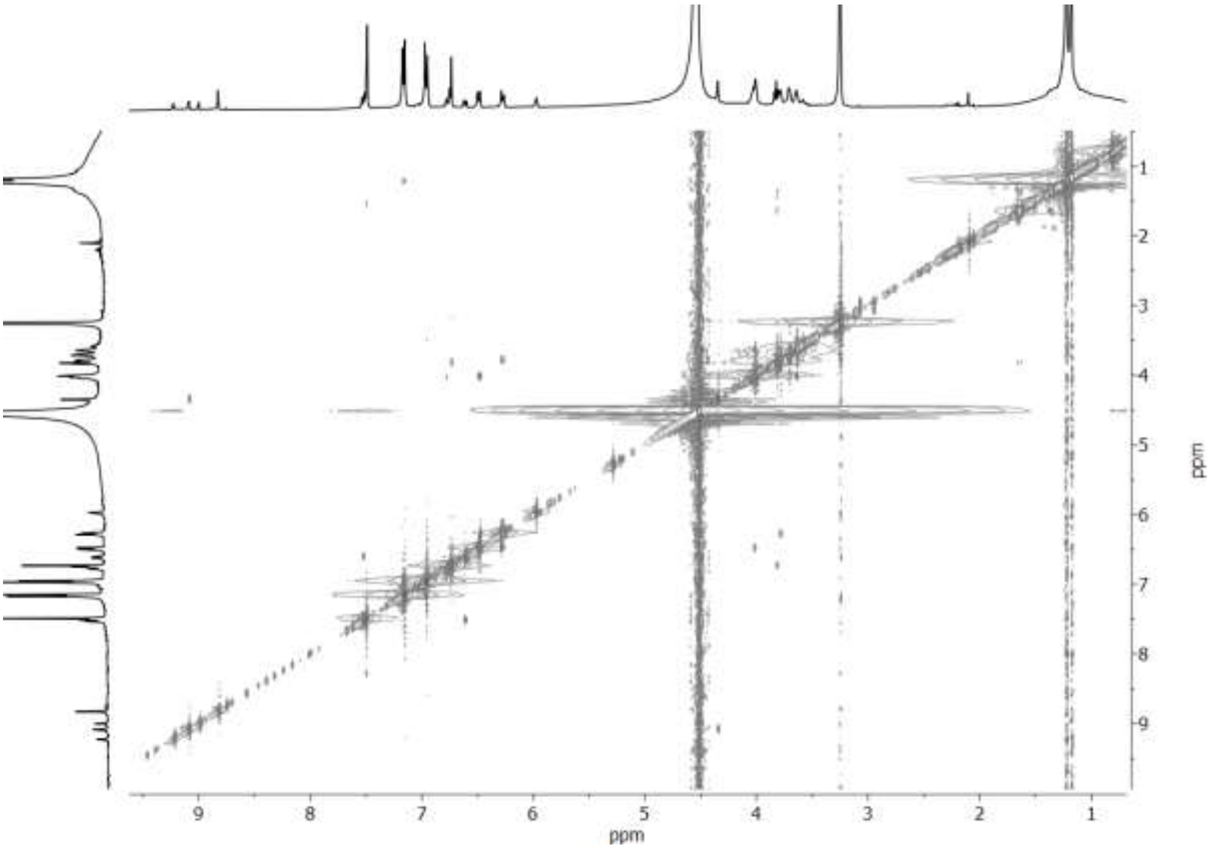

Figure S24. *NOESY NMR spectrum of **5·PF₆** (600 MHz, 1:1 MeOD:CDCl₃)*

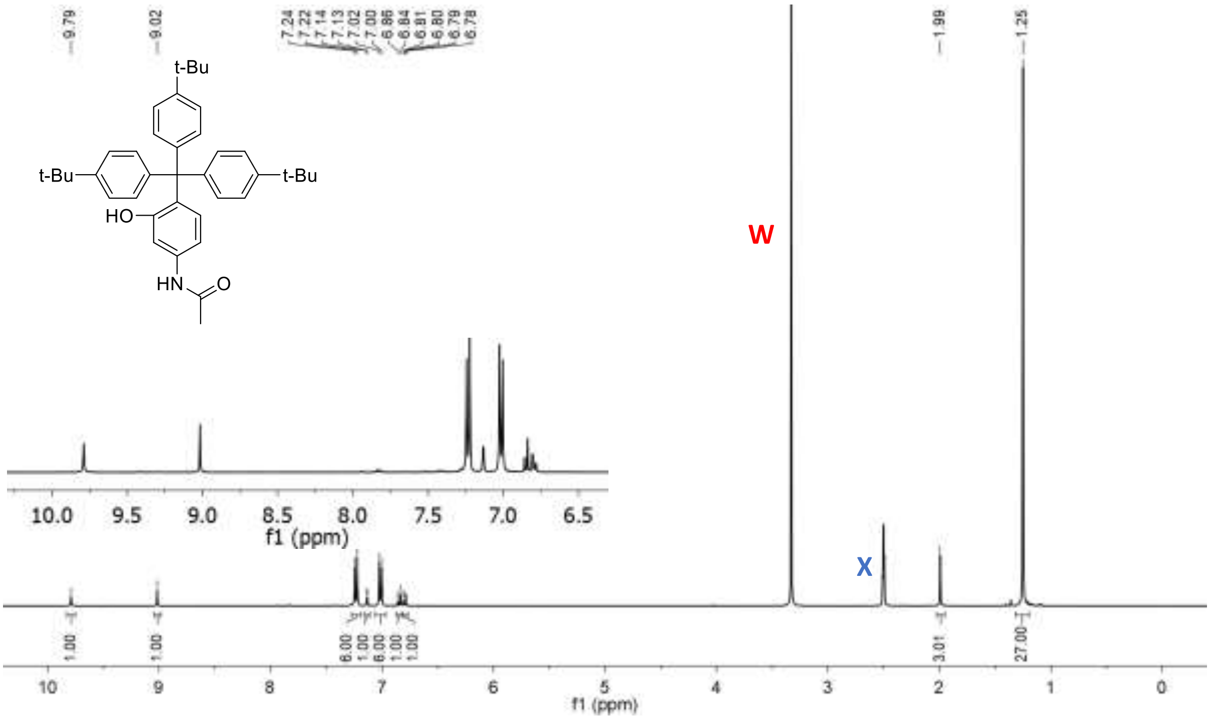

Figure S25. *^{1}H NMR spectrum of **15**; peak labelled **X** results from incompletely deuterated NMR solvent, residual water peak marked by **W**, (400 MHz, DMSO-d$_6$).*

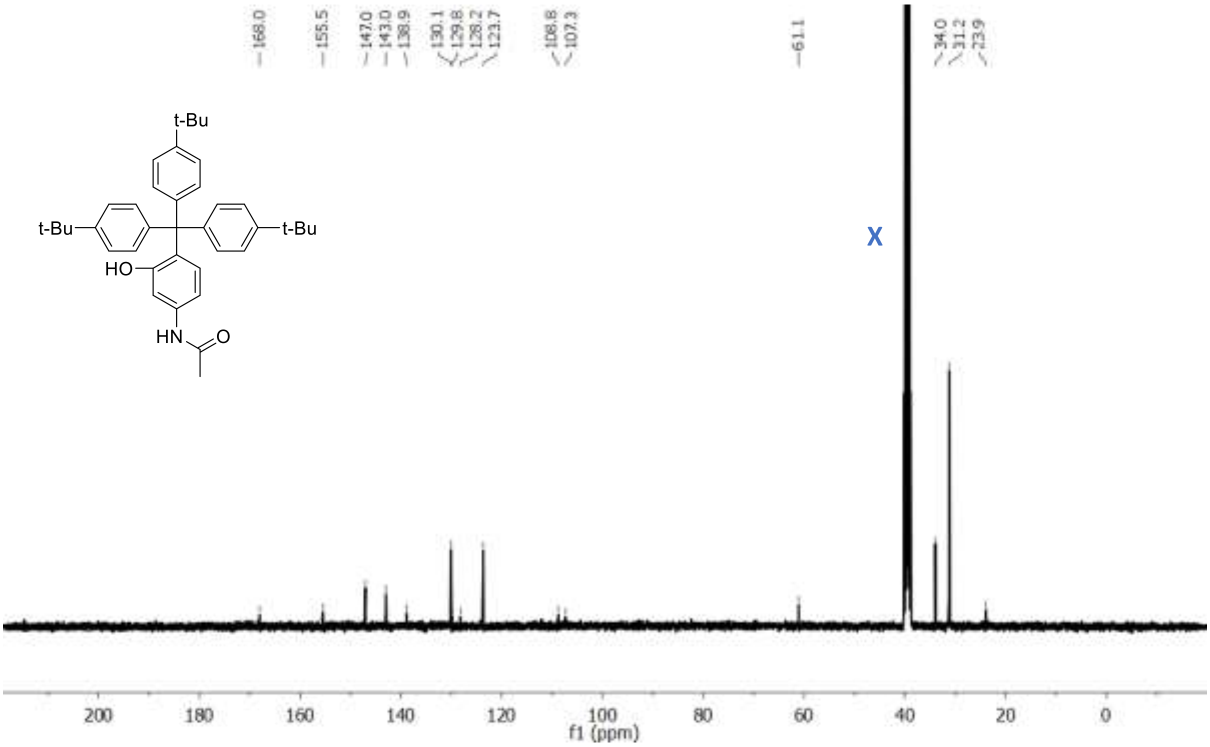

Figure S26. *^{13}C{^{1}H} NMR spectrum of **15**; peak labelled **X** results from incompletely deuterated NMR solvent (101 MHz, DMSO-d$_6$).*

Difficulties encountered in the synthesis of 15

Literature precedent[8] based on the trityl analogue (*i.e.* without the *t*-butyl groups) indicated that **6** could be formed by an acid catalysed electrophilic aromatic substitution of *m*-aminophenol with **8** in the presence of conc. HCl and acetic acid. Unfortunately, no reaction was observed in initial reactions, with decomposition products observed if the reaction time was extended. We never observed formation of significant amounts of **6** and instead found that **15** could sometimes be isolated, but only when *m*-aminophenol was used in excess and after extensive purification and this was not reproducible. Due to difficulties in obtaining a consistent result from the reaction the method was therefore changed to the one described in the main text.

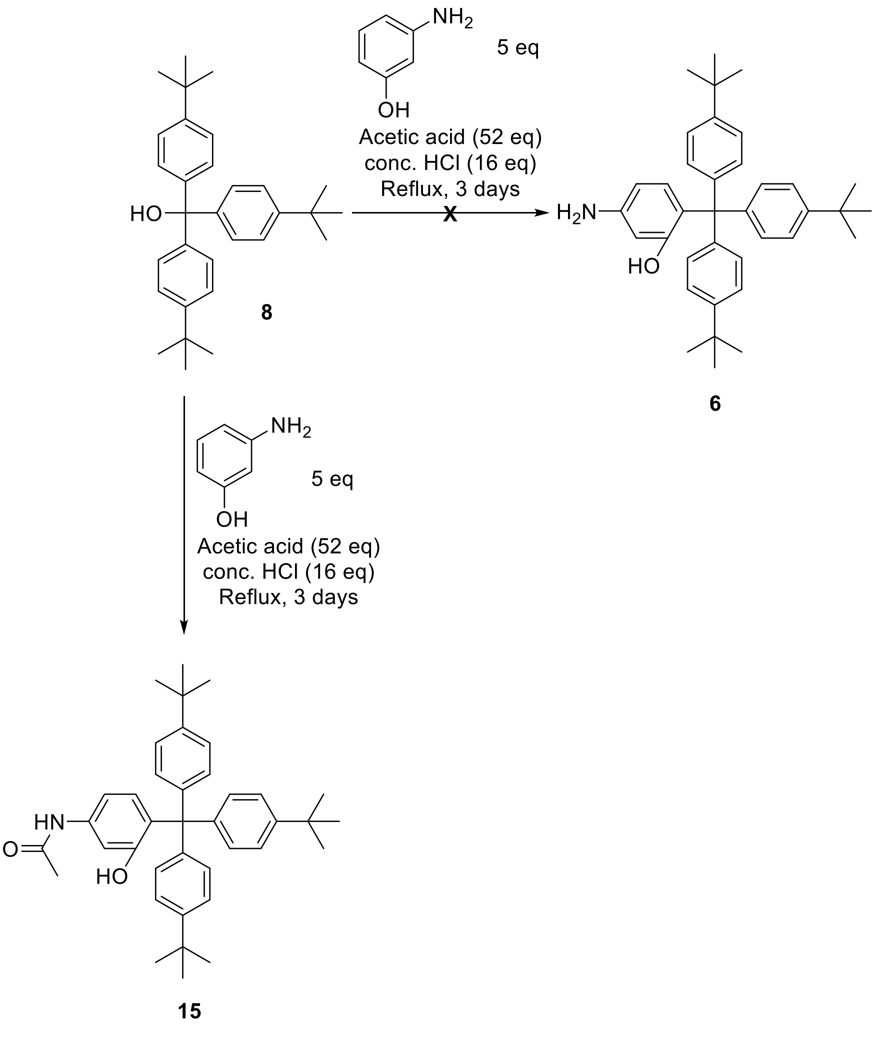

Scheme S1. *Synthesis of* ***15****.*

Quantitative anion binding data

General protocol

Anion binding titration experiments were conducted at 298 K. Initial sample volumes were 0.50 mL and concentrations were 2.0 mmol L^{-1} of host. Anion solutions (100 mmol L^{-1}) as their tetrabutylammonium (TBA) salts were added in aliquots, the samples thoroughly shaken and spectra recorded at 0, 0.2, 0.4, 0.6, 0.8, 1.0, 1.2, 1.4, 1.6, 1.8, 2.0, 2.5, 3.0, 4.0, 5.0, 7.0 and 10 equivalents of anion. Data were fitted using the Bindfit program hosted at Supramolecular.org.[9]

Anion binding data, NMR spectra and isotherms

The following graphs show the data and fitted isotherms used to determine association constants. A global fit was used where possible to determine the association constants, with the resonance movement of peaks **a**, **b** and **c** (Figure S27) being recorded and subsequently graphed. Peak resonances that had significant overlap or broadening were not used, causing some association constants to be determined by resonance **a** alone.

The stacked ^1H NMR spectra (Figures S28-S57) show the titration data obtained from the binding studies between **4·PF$_6$**, **5·PF$_6$** and **1·PF$_6$** and anions as their TBA salts. The stacked ^1H NMR spectra each contain 17 spectra corresponding to the equivalents added, with spectrum **1** (the bottom spectrum) corresponding to 0 equivalents of anion while spectrum **17** (the top spectrum) corresponds to 10 equivalents of anion. Full binding data including all fitting parameters are available at the corresponding URLs.

For the majority of the titrations the receptors' O−H and N−H proton resonance either disappears or broadens and weakens significantly due to H/D exchange.

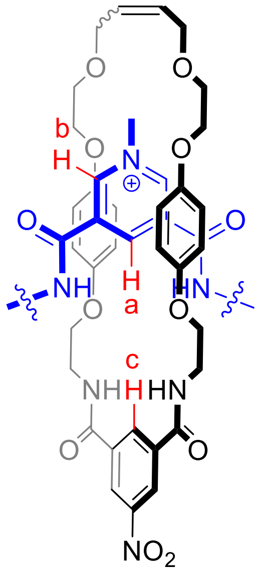

Figure S27. *Structure of **1**$^+$, **4**$^+$, and **5**$^+$, showing global fit proton resonances in red.*

4·PF_6 in 1:1 $CDCl_3$:$CDOD_3$

4·PF_6/TBA·Cl: K_a = 6370 ± 8% M^{-1}
http://app.supramolecular.org/bindfit/view/b6885423-fc31-4326-9c1a-e40e121da1ef

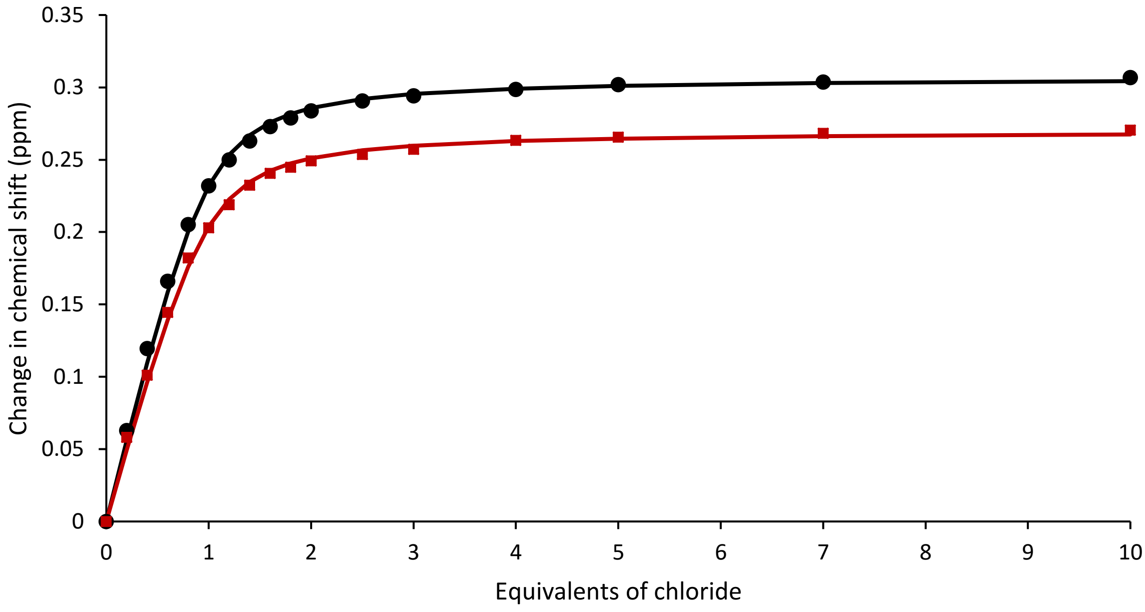

Figure S28. *Movement of peaks at 9.09 ppm (black) and 8.97 ppm (red) upon addition of TBA·Cl (1:1 $CDCl_3$:CD_3OD, 298 K). Points represent observed data, lines represent 1:1 isotherm calculated using Bindfit.*[9]

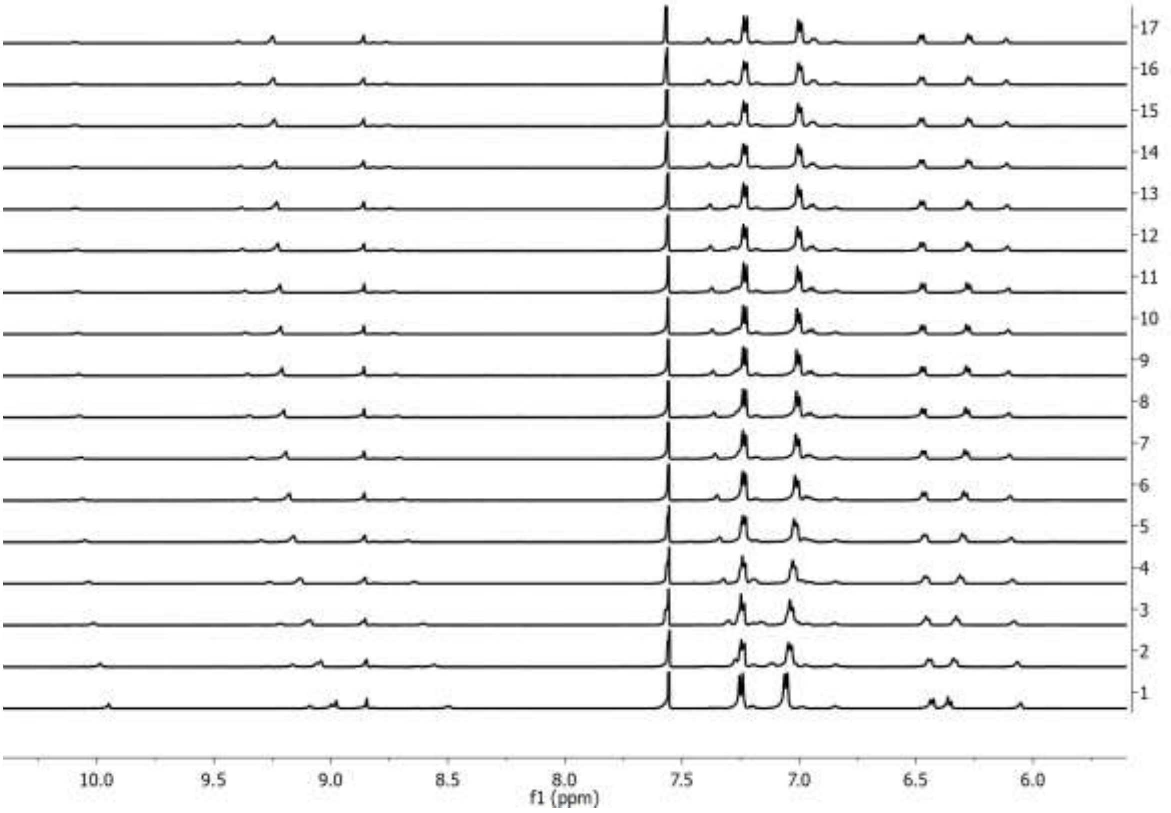

Figure S29. *Truncated* [1]*H NMR spectra of **4·PF_6** upon addition of increasing equivalents of TBA·Cl (1:1 $CDCl_3$:CD_3OD, 298 K, 600 MHz).*

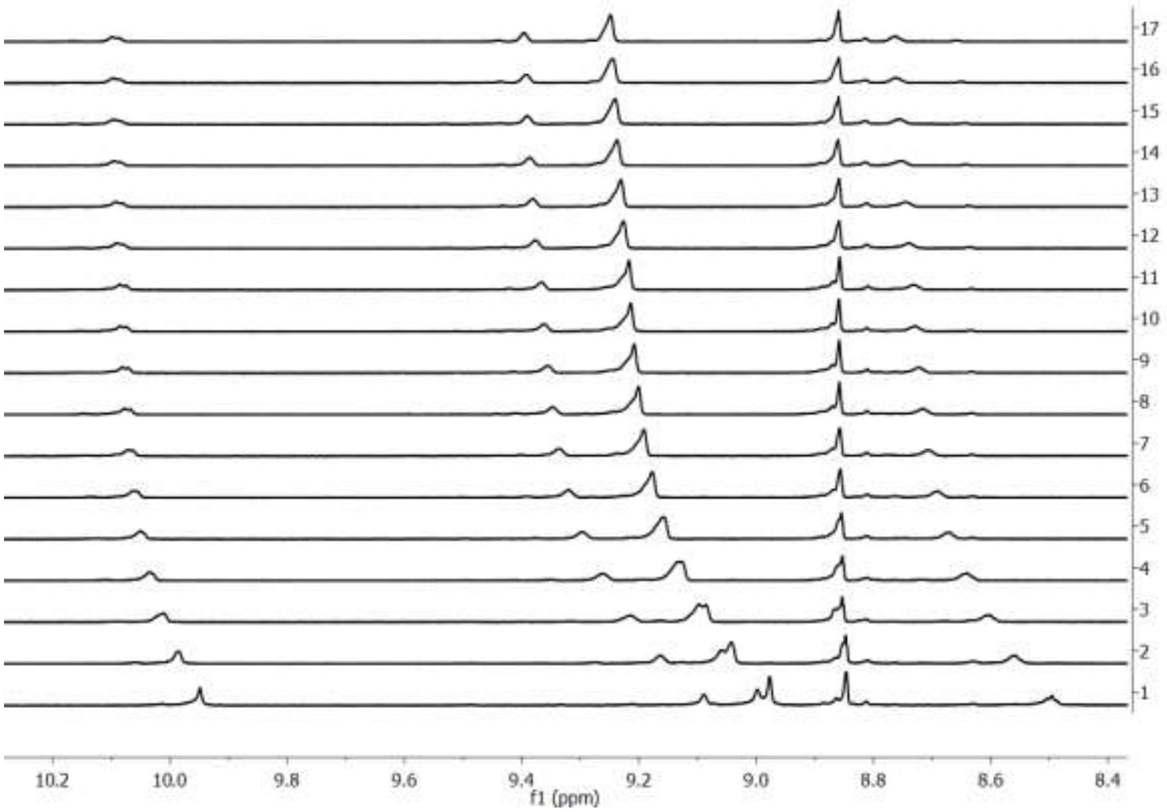

Figure S30. *Truncated ^{1}H NMR spectra of **4·PF$_6$** upon addition of increasing equivalents of TBA·Cl showing downfield region of spectrum (1:1 $CDCl_3$:CD_3OD, 298 K, 600 MHz).*

4·PF$_6$/TBA·I: K_a = 1122 ± 6% M^{-1}

http://app.supramolecular.org/bindfit/view/7145d153-5478-4147-acde-d80fb0a8c732

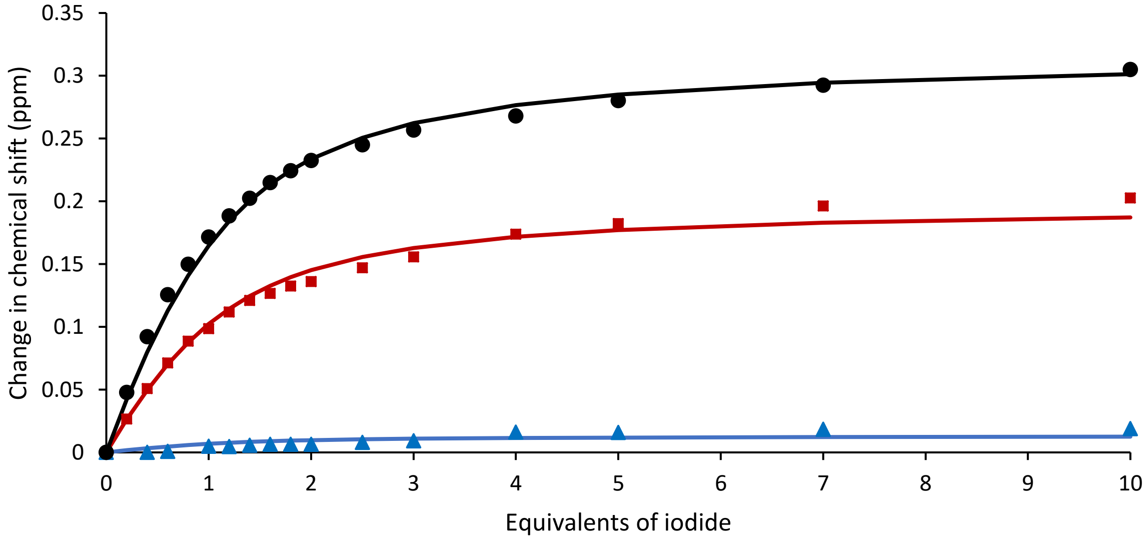

Figure S31. *Movement of peaks at 9.08 ppm (red), 9.00 pm (blue) and 8.97 ppm (black) upon addition of TBA·I (1:1 $CDCl_3$:CD_3OD, 298 K). Points represent observed data, lines represent 1:1 isotherm calculated using Bindfit.*[9]

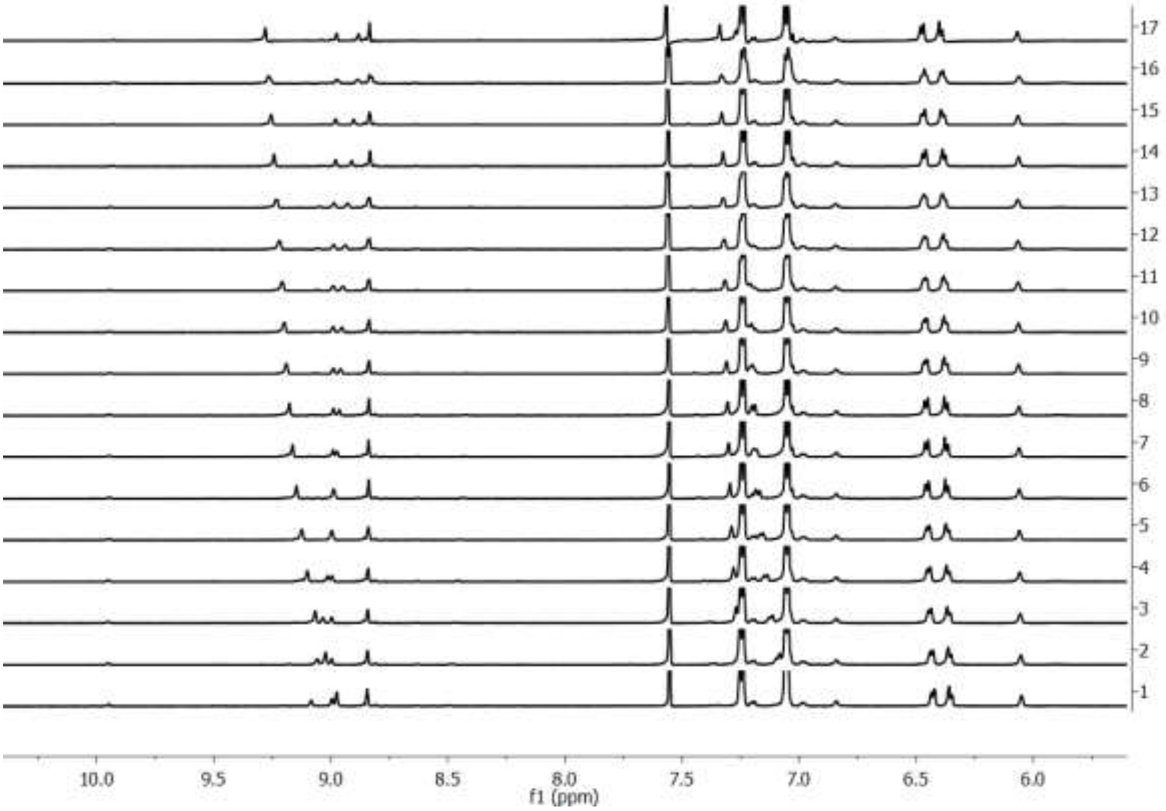

Figure S32. *Truncated ^{1}H NMR spectra of* ***4·PF$_6$*** *upon addition of increasing equivalents of TBA·I (1:1 $CDCl_3$:CD_3OD, 298 K, 600 MHz).*

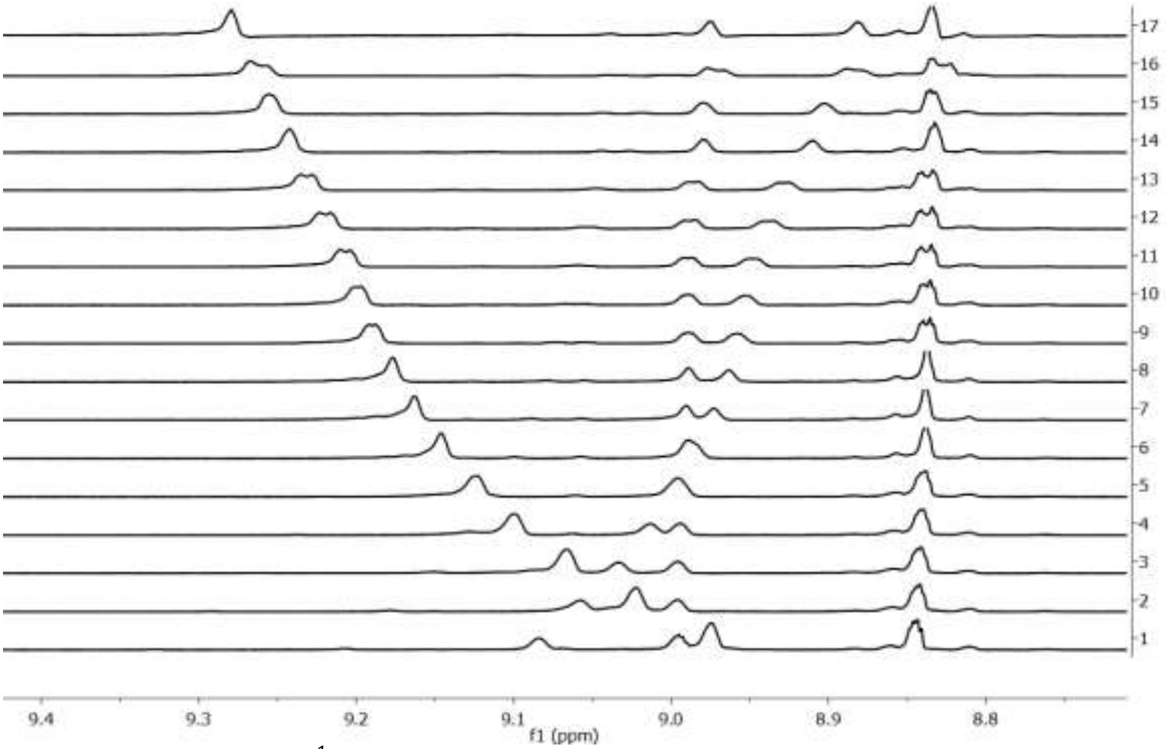

Figure S33. *Truncated ^{1}H NMR spectra of* ***4·PF$_6$*** *upon addition of increasing equivalents of TBA·I showing downfield region of spectrum (1:1 $CDCl_3$:CD_3OD, 298 K, 600 MHz).*

4·PF$_6$/TBA·OAc: K_a = 400 ± 8% M^{-1}

http://app.supramolecular.org/bindfit/view/3b9a8e23-812b-4ee8-86a9-7a72b7fd334c

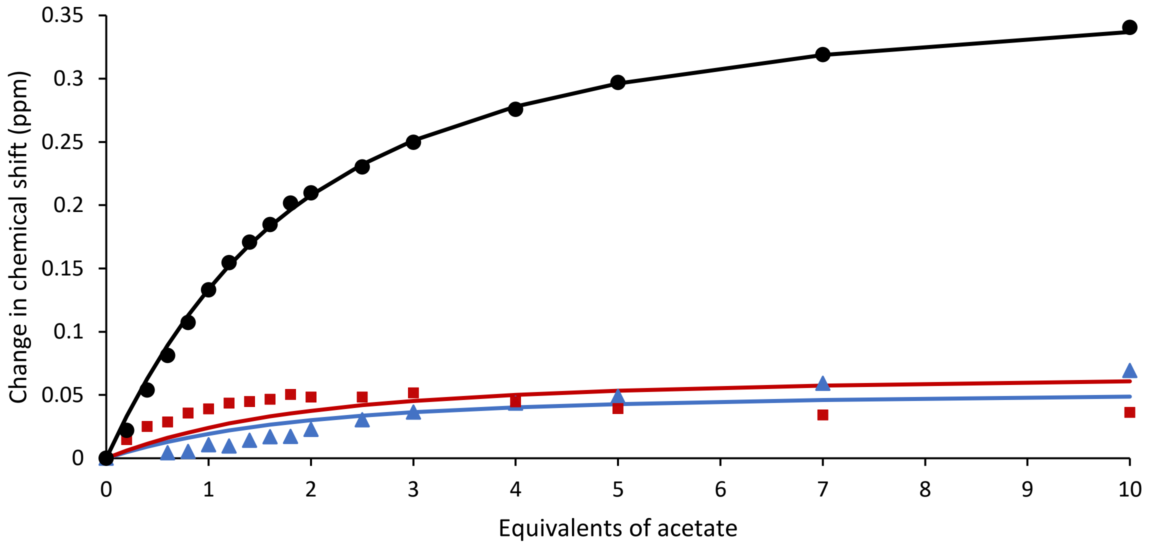

Figure S34. *Movement of peaks at 9.08 ppm (blue), 8.99 ppm (red) and 8.97 ppm (black) upon addition of TBA·OAc (1:1 $CDCl_3$:CD_3OD, 298 K). Points represent observed data, lines represent 1:1 isotherm calculated using Bindfit.*[9]

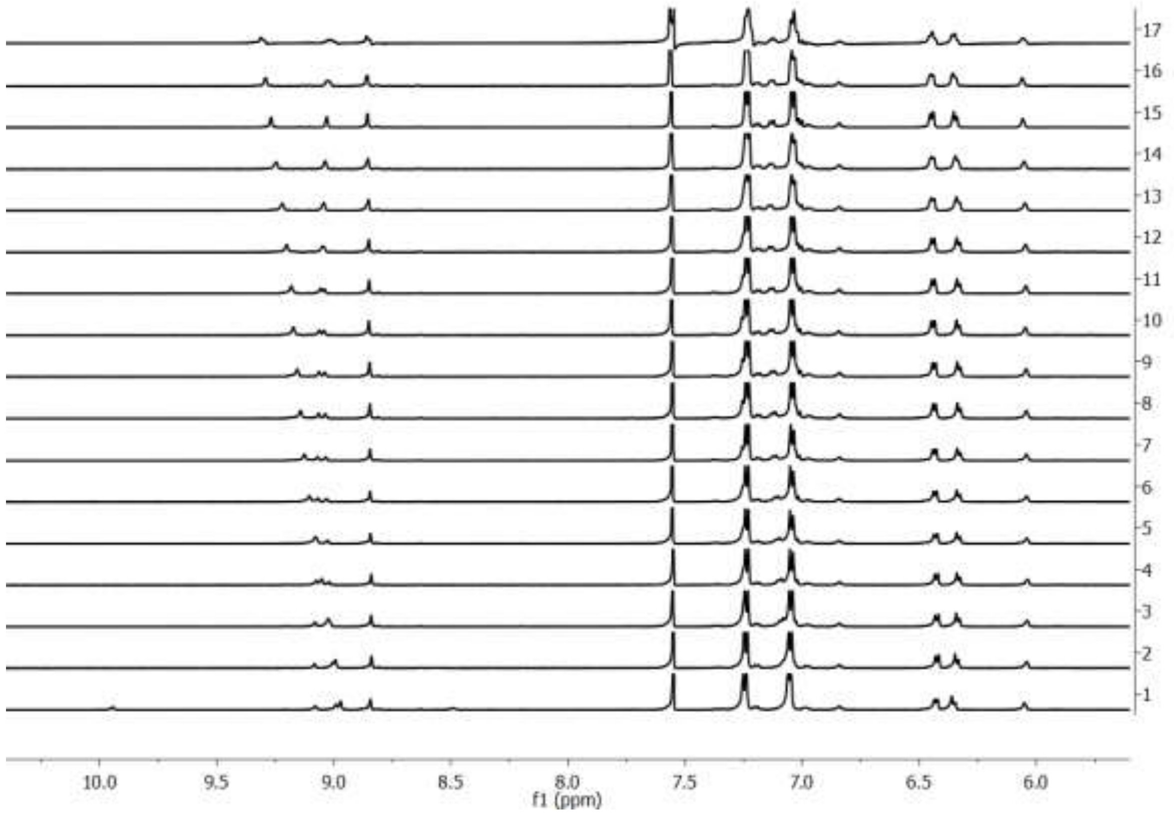

Figure S35. *Truncated ^{1}H NMR spectra of **4·PF$_6$** upon addition of increasing equivalents of TBA·OAc (1:1 $CDCl_3$:CD_3OD, 298 K, 600 MHz).*

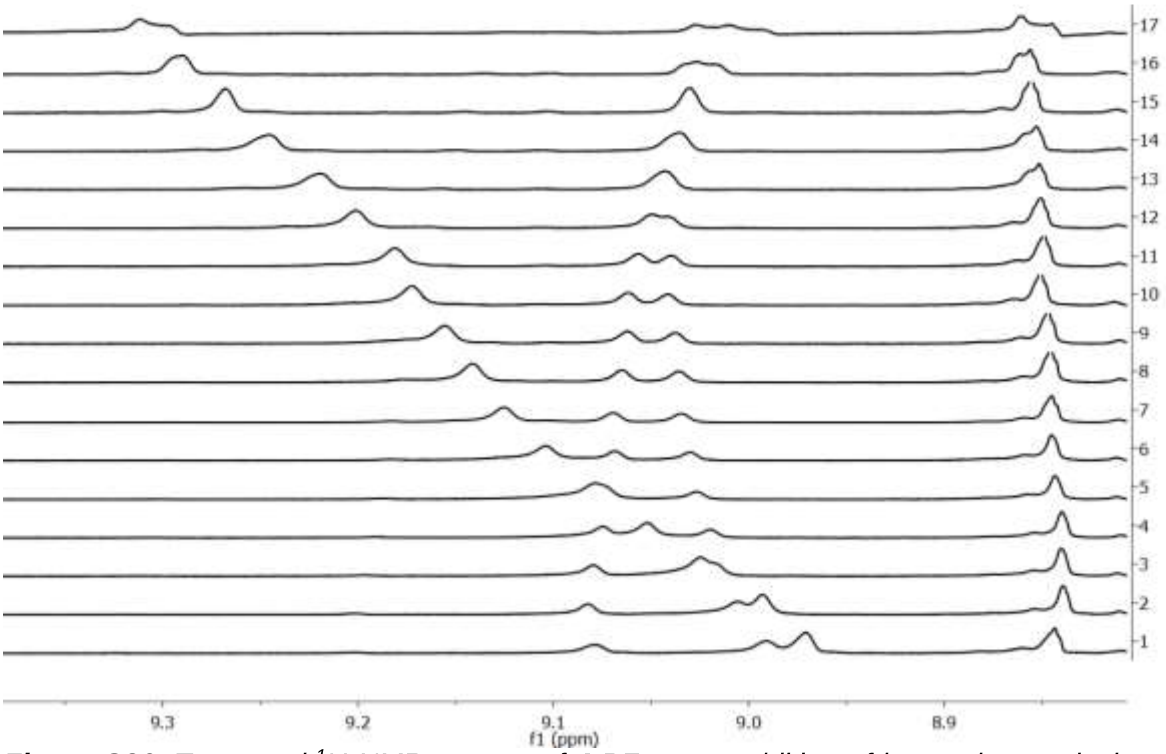

Figure S36. *Truncated ^{1}H NMR spectra of* ***4·PF$_6$*** *upon addition of increasing equivalents of TBA·OAc showing downfield region of spectrum (1:1 $CDCl_3$:CD_3OD, 298 K, 600 MHz).*

4·PF$_6$/TBA· H_2PO_4: K_a = 122 ± 8% M^{-1}
http://app.supramolecular.org/bindfit/view/92afe25c-edb1-400a-a462-d81d9bdcb877

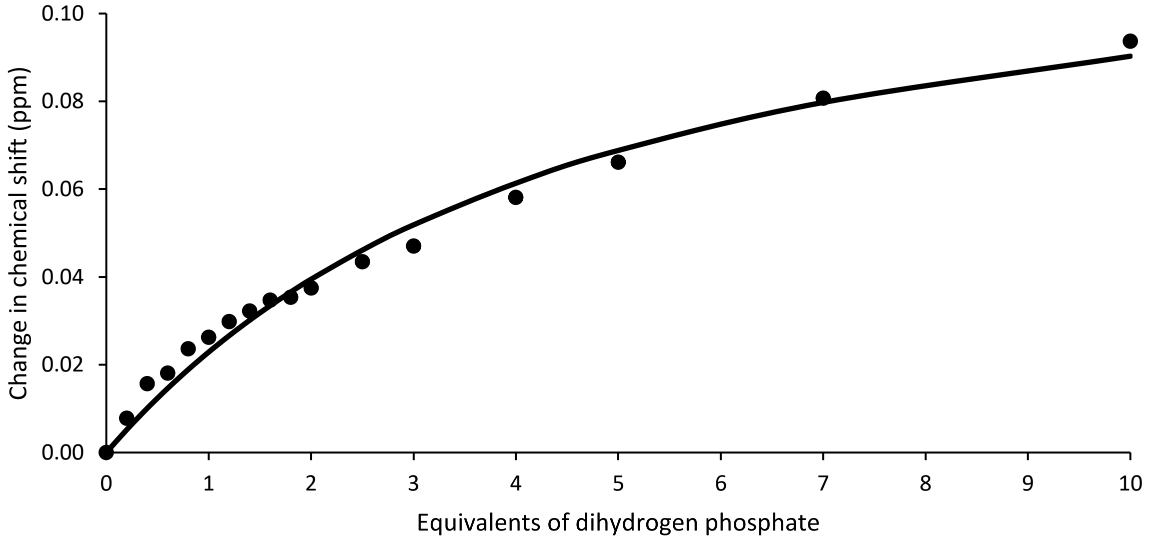

Figure S37. *Movement of peak at 9.09 ppm upon addition of TBA·H_2PO_4 (1:1 $CDCl_3$:CD_3OD, 298 K). Points represent observed data, line represents 1:1 isotherm calculated using Bindfit.*[9]

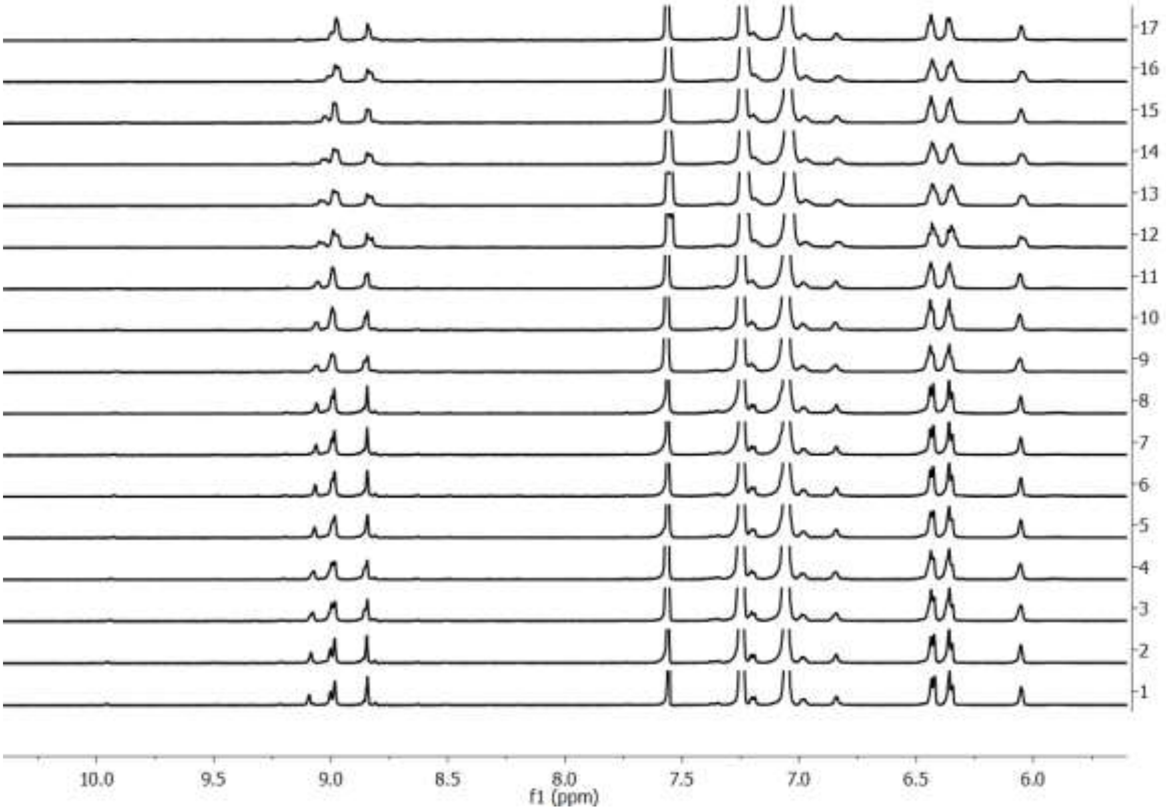

Figure S38. *Truncated ^{1}H NMR spectra of **4·PF6** upon addition of increasing equivalents of TBA·H_2PO_4 (1:1 $CDCl_3$:CD_3OD, 298 K, 600 MHz).*

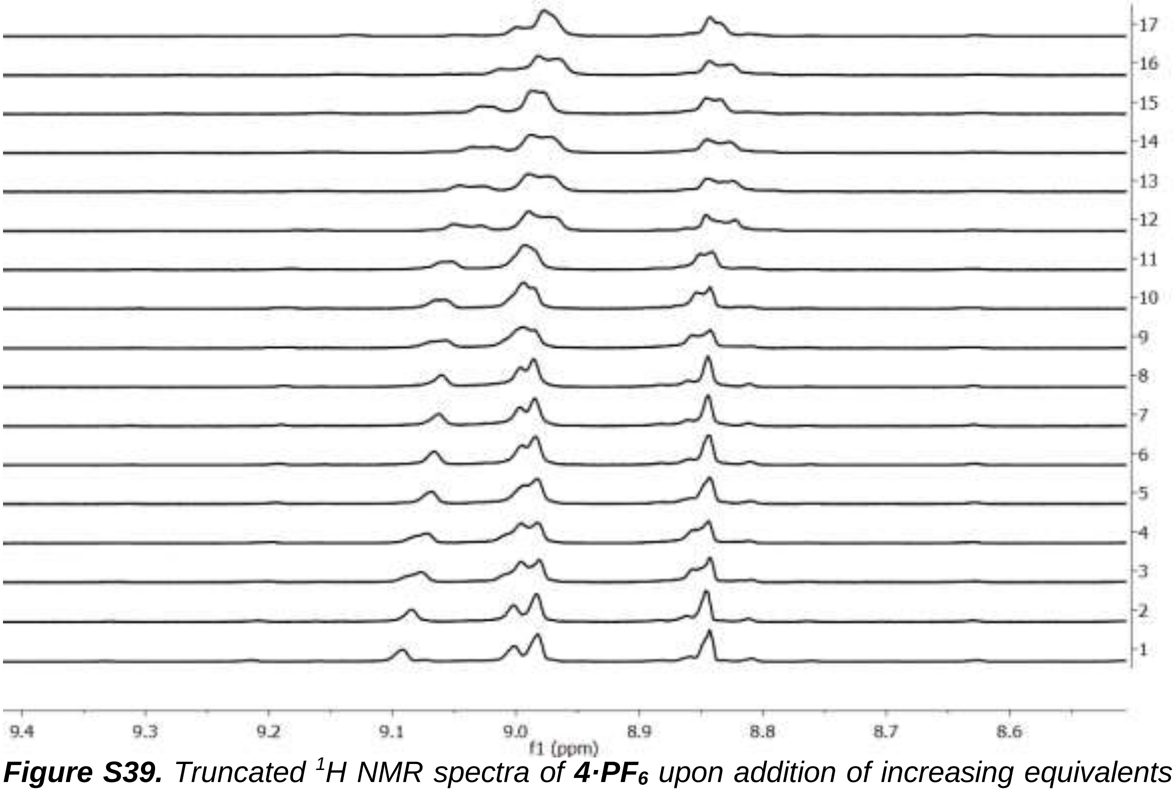

Figure S39. *Truncated ^{1}H NMR spectra of **4·PF6** upon addition of increasing equivalents of TBA·H_2PO_4 showing downfield region of spectrum (1:1 $CDCl_3$:CD_3OD, 298 K, 600 MHz).*

5·PF_6 in 1:1 $CDCl_3$:$CDOD_3$

5·PF_6/TBA·Cl: K_a = 771 ± 5% M^{-1}
http://app.supramolecular.org/bindfit/view/6c149045-7711-4252-8e98-db8dba680b30

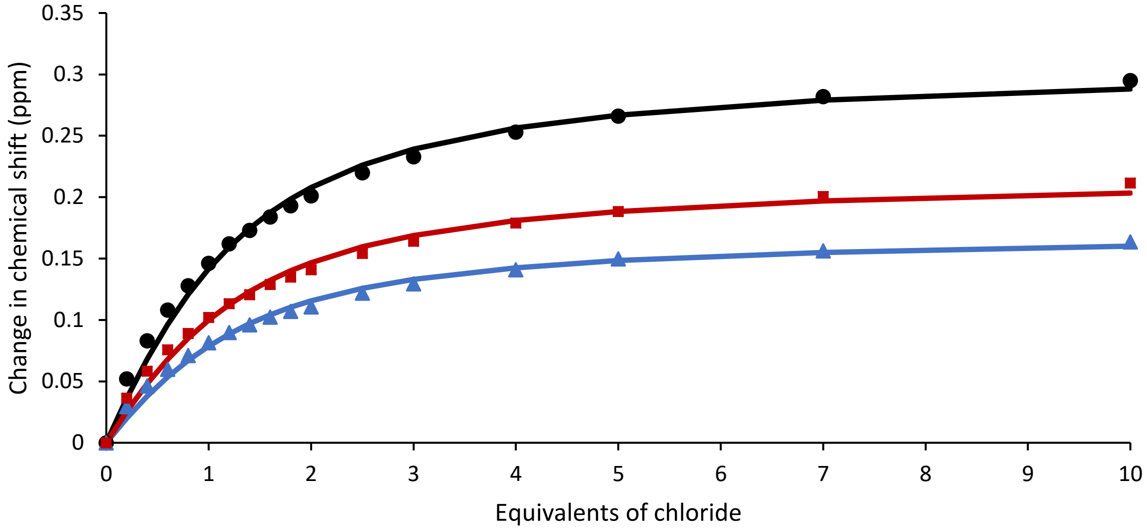

Figure S40. *Movement of peaks at 9.06 ppm (black), 9.03 ppm (blue) and 8.90 ppm (red) upon addition of TBA·Cl (1:1 $CDCl_3$:CD_3OD, 298 K). Points represent observed data, lines represent 1:1 isotherm calculated using Bindfit.*[9]

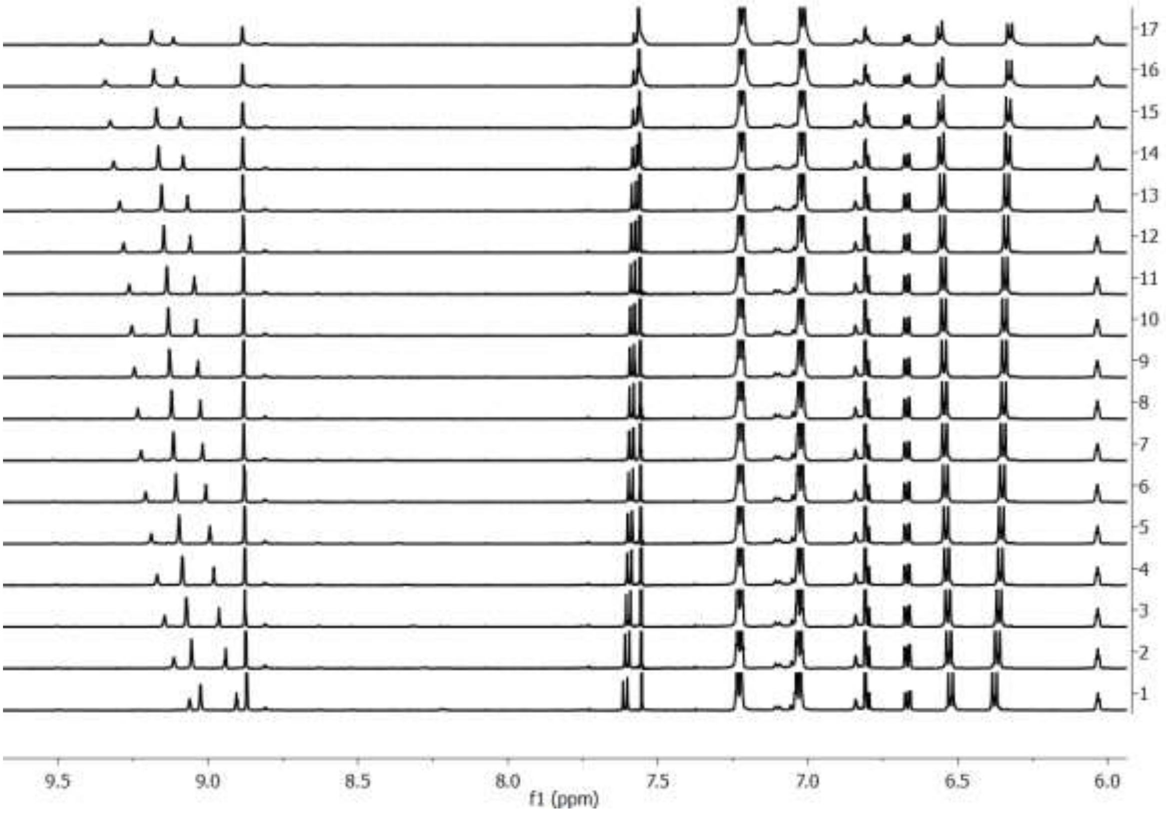

Figure S41. *Truncated* [1]*H NMR spectra of **5·PF_6** upon addition of increasing equivalents of TBA·Cl (1:1 $CDCl_3$:CD_3OD, 298 K, 600 MHz).*

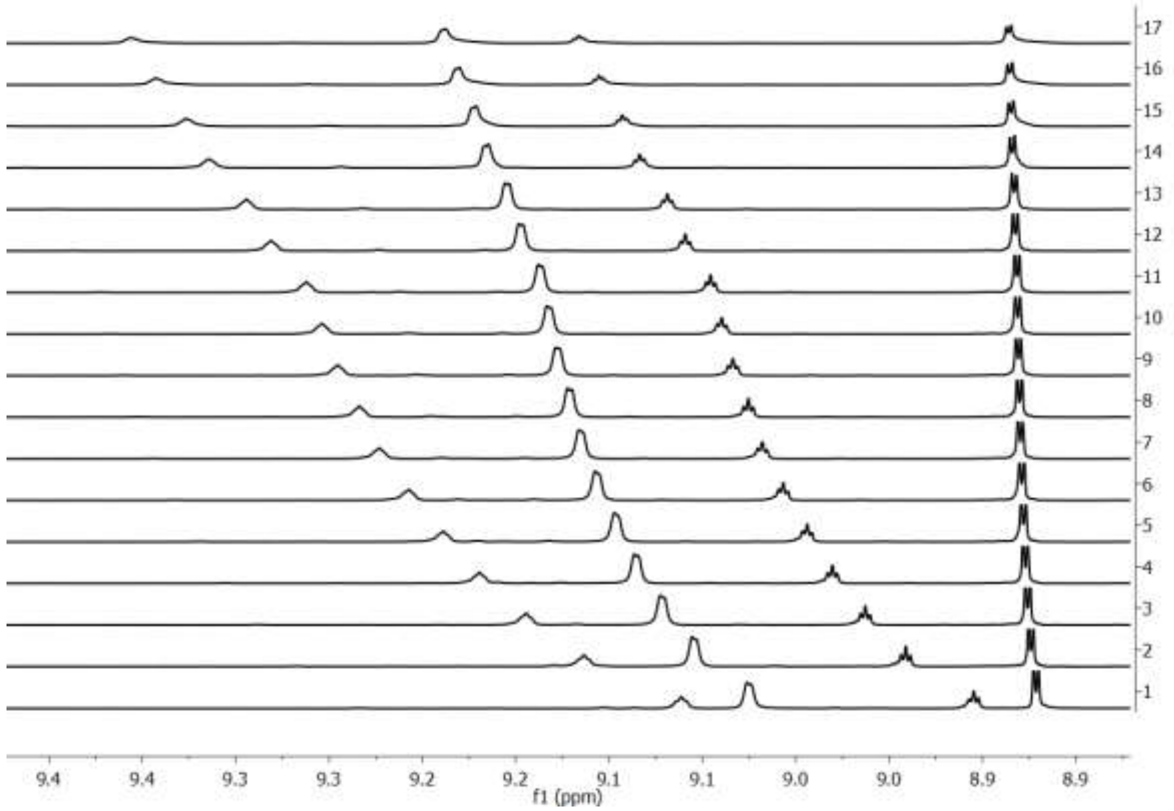

Figure S42. *Truncated ^{1}H NMR spectra of **5·PF₆** upon addition of increasing equivalents of TBA·Cl showing downfield region of spectrum (1:1 $CDCl_3$:CD_3OD, 298 K, 600 MHz).*

5·PF_6/TBA·I: K_a = 157 ± 4% M^{-1}
http://app.supramolecular.org/bindfit/view/2c70b2f7-0122-4ae4-84b9-bc1ecdc8feaa

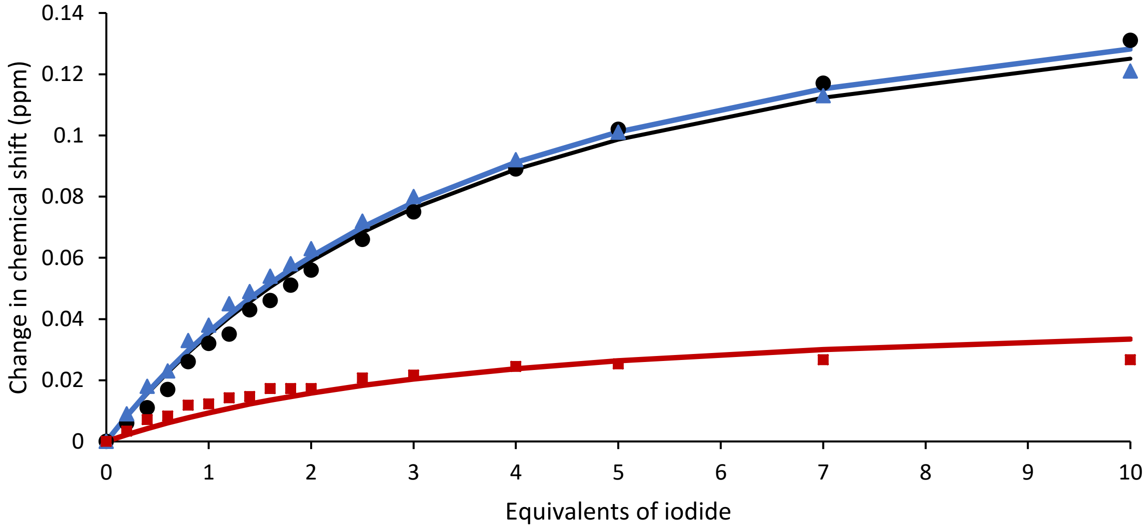

Figure S43. *Movement of peaks at 9.06 ppm (black), 9.03 ppm (blue) and 8.90 ppm (red) upon addition of TBA·I (1:1 $CDCl_3$:CD_3OD, 298 K). Points represent observed data, lines represent 1:1 isotherm calculated using Bindfit.*[9]

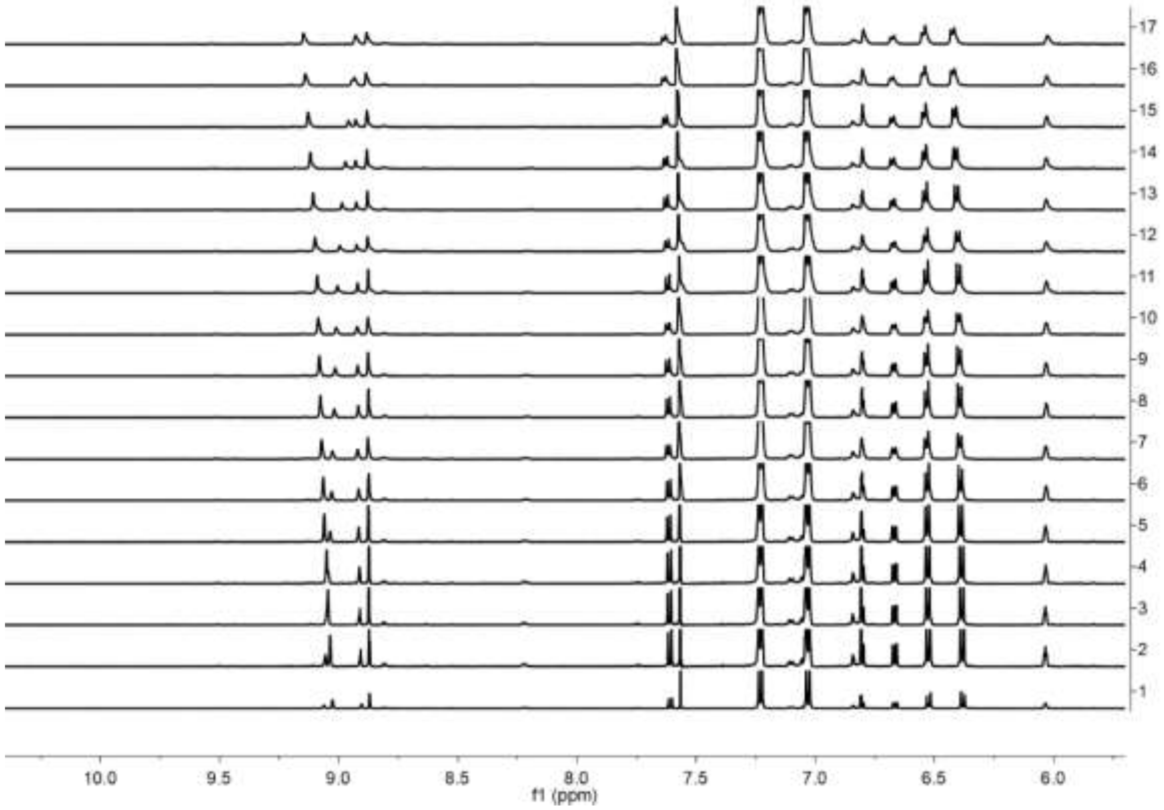

Figure S44. *Truncated* [1]*H NMR spectra of* ***5·PF₆*** *upon addition of increasing equivalents of TBA·I (1:1 $CDCl_3$:CD_3OD, 298 K, 600 MHz).*

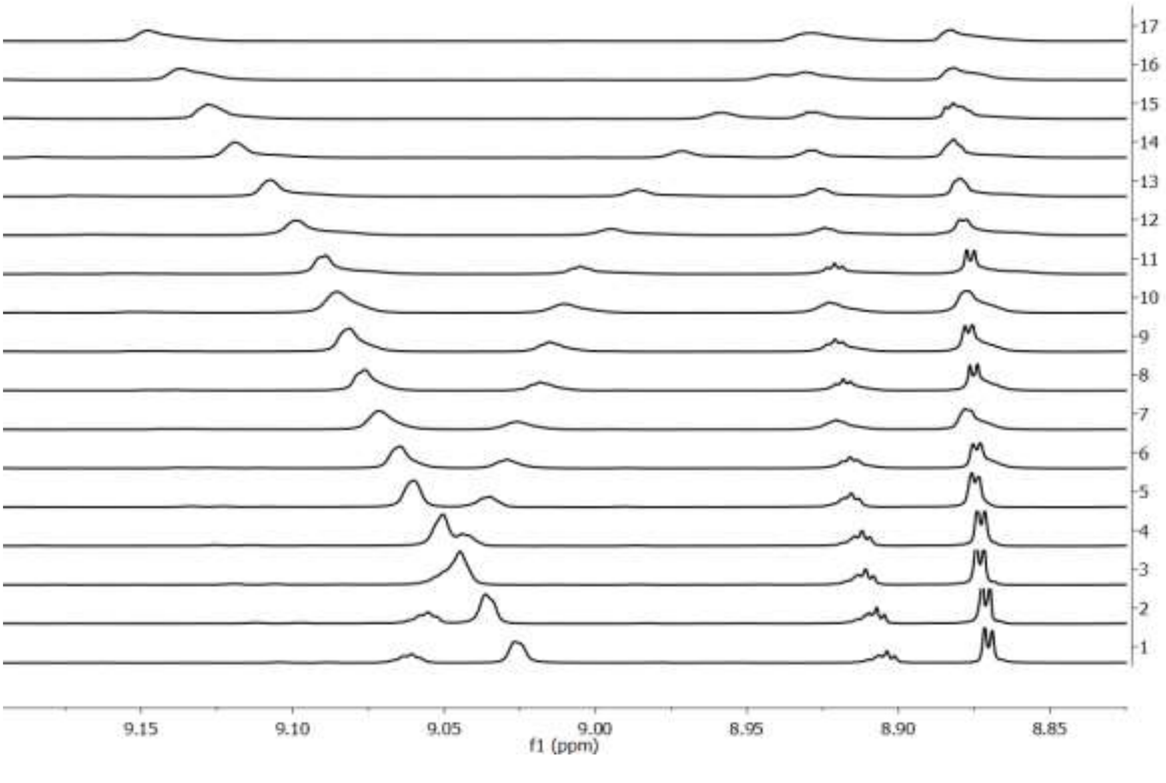

Figure S45. *Truncated* [1]*H NMR spectra of* ***5·PF₆*** *upon addition of increasing equivalents of TBA·I showing downfield region of spectrum (1:1 $CDCl_3$:CD_3OD, 298 K, 600 MHz).*

5·PF_6/TBA·OAc: K_{11} = 210 ± 5% M^{-1}; K_{12} = 175 ± 6% M^{-1}
http://app.supramolecular.org/bindfit/view/e37a10bb-b23c-4862-9ce5-f2f8ef94cb05

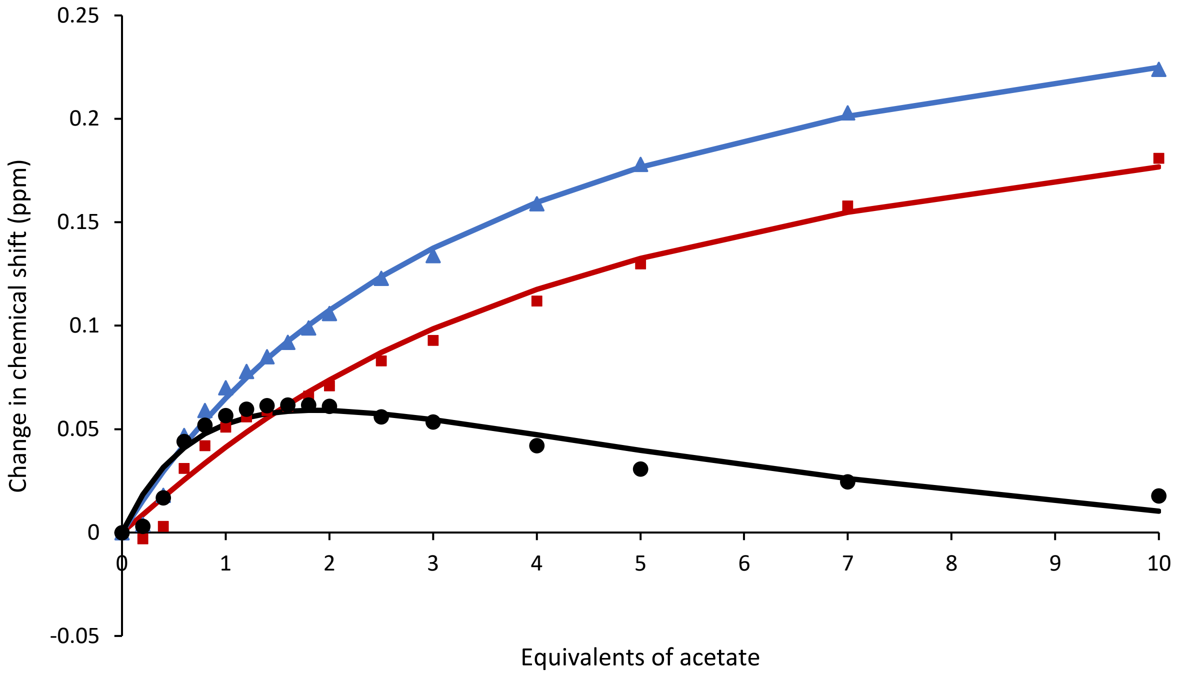

Figure S46. *Movement of peaks at 9.08 ppm (red), 9.03 ppm (blue) and 8.91 ppm (black) upon addition of TBA·OAc (1:1 $CDCl_3$:CD_3OD, 298 K). Points represent observed data, lines represent 1:1 isotherm calculated using Bindfit.*[9]

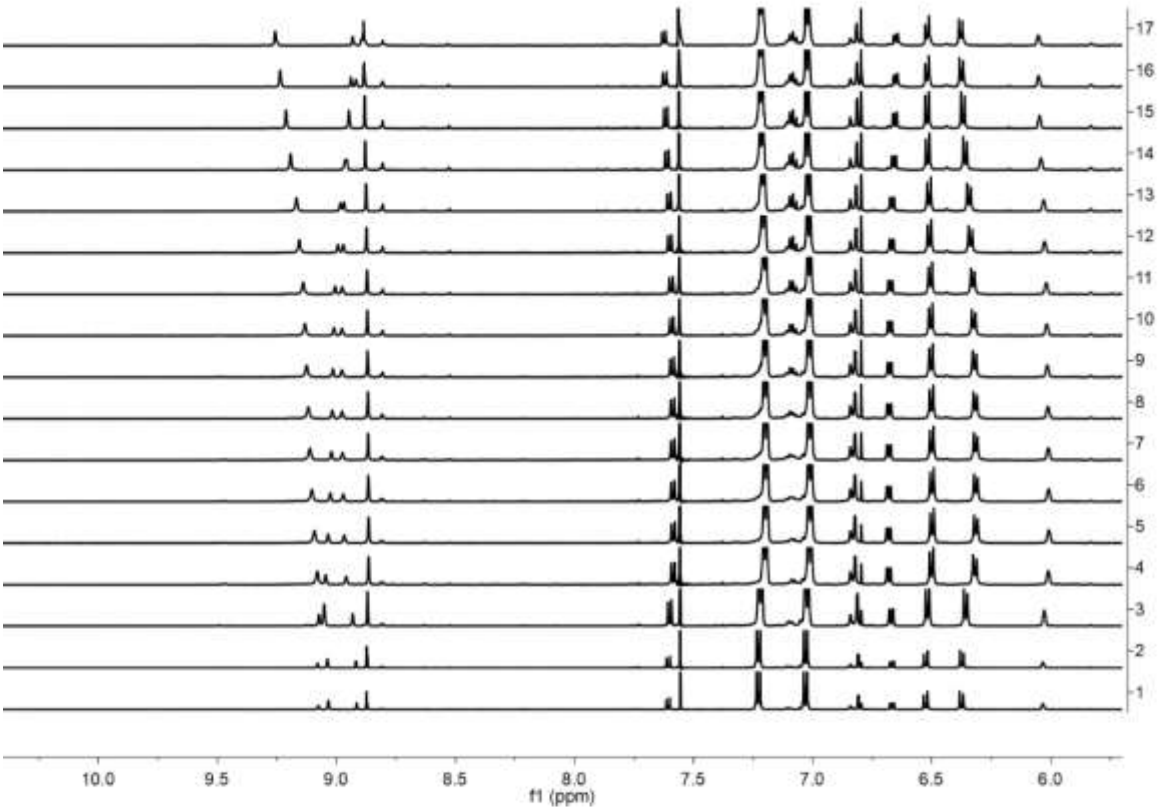

Figure S47. *Truncated ^{1}H NMR spectra of **5·PF_6** upon addition of increasing equivalents of TBA·OAc (1:1 $CDCl_3$:CD_3OD, 298 K, 600 MHz).*

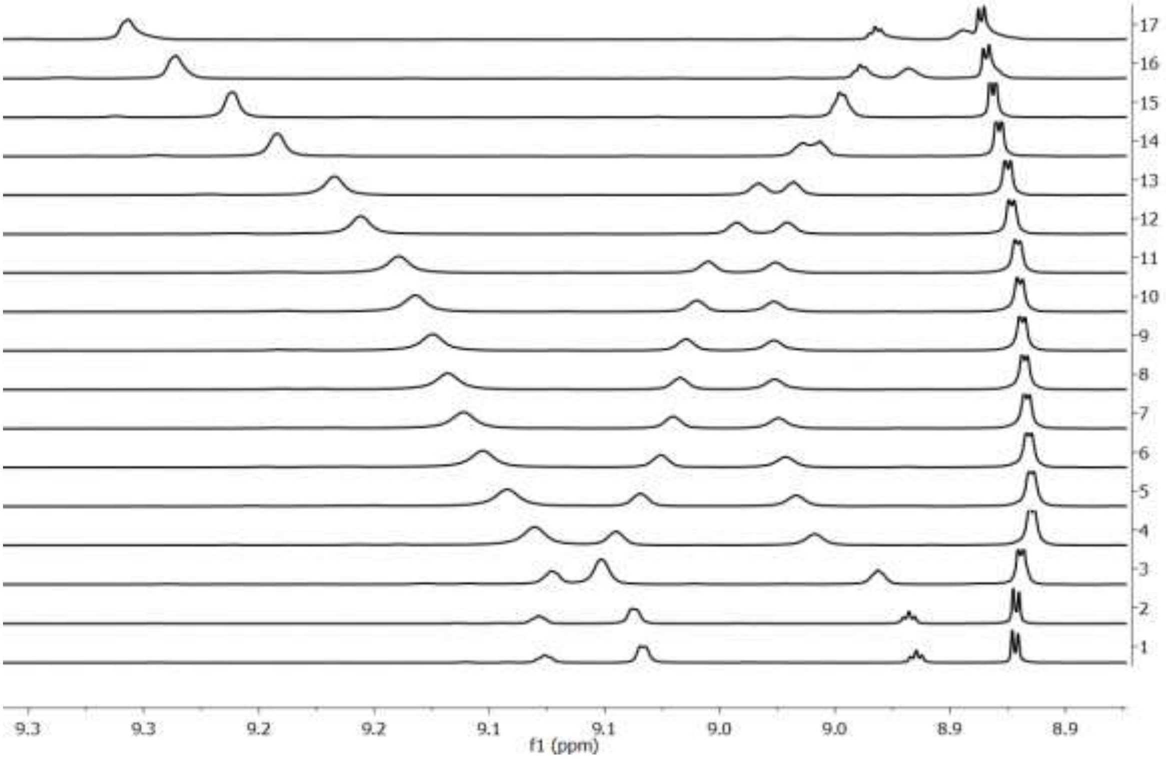

Figure S48. *Truncated ^{1}H NMR spectra of* ***5·PF₆*** *upon addition of increasing equivalents of TBA·OAc showing downfield region of spectrum (1:1 $CDCl_3$:CD_3OD, 298 K, 600 MHz).*

5·PF$_6$/TBA·H$_2$PO$_4$: K_{11} = 1813 ± 11% M^{-1}; K_{12} = 354 ± 11% M^{-1}

http://app.supramolecular.org/bindfit/view/0bfb1c38-fcf0-4e5d-8b10-bcdcc3d2c05d

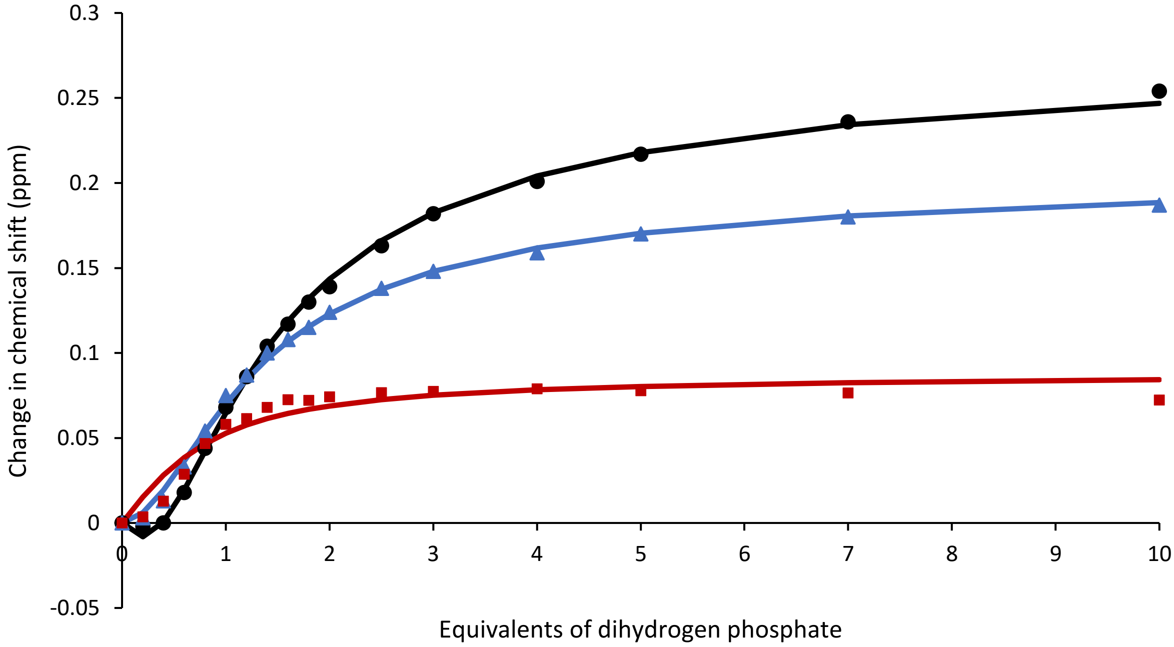

Figure S49. *Movement of peaks at 9.07 ppm (black), 9.03 ppm (blue) and 8.91 ppm (red) upon addition of TBA·H_2PO_4 (1:1 $CDCl_3$:CD_3OD, 298 K). Points represent observed data, lines represent 1:1 isotherm calculated using Bindfit.*[9]

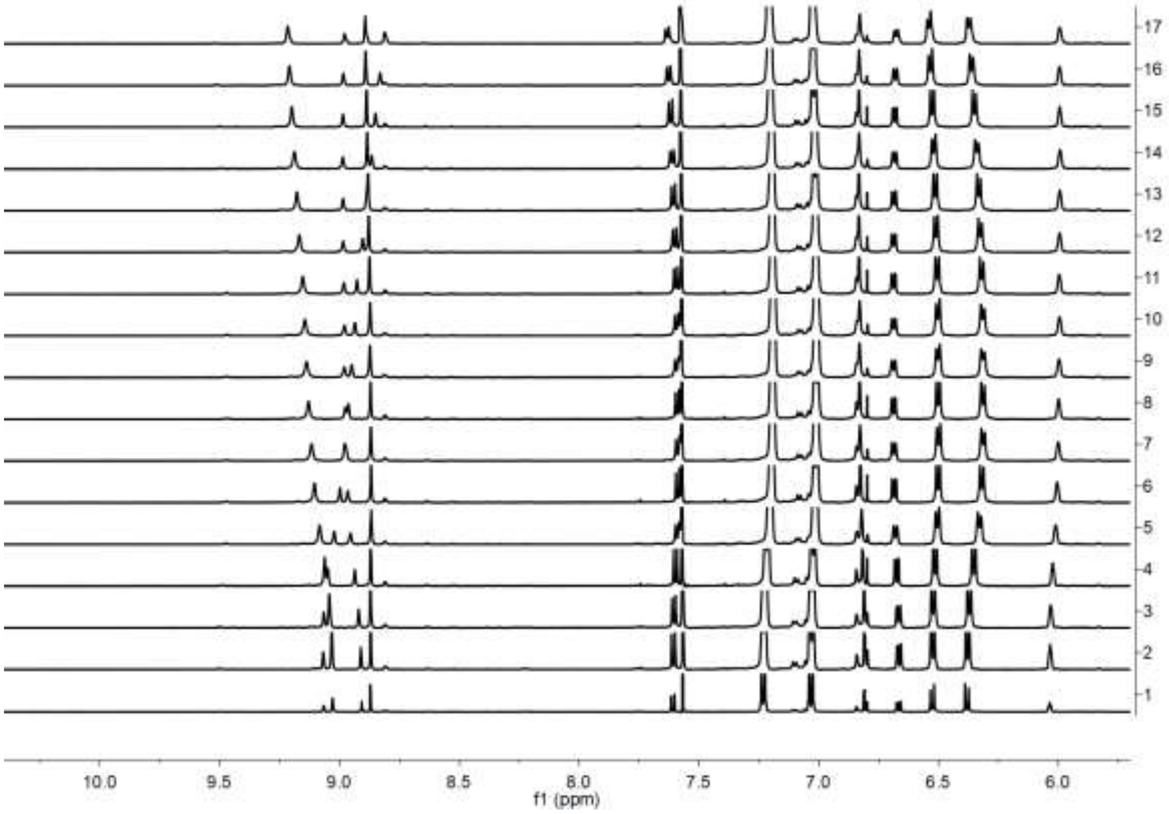

Figure S50. *Truncated* [1]*H NMR spectra of* ***5·PF$_6$*** *upon addition of increasing equivalents of TBA·H_2PO_4 (1:1 $CDCl_3$:CD_3OD, 298 K, 600 MHz).*

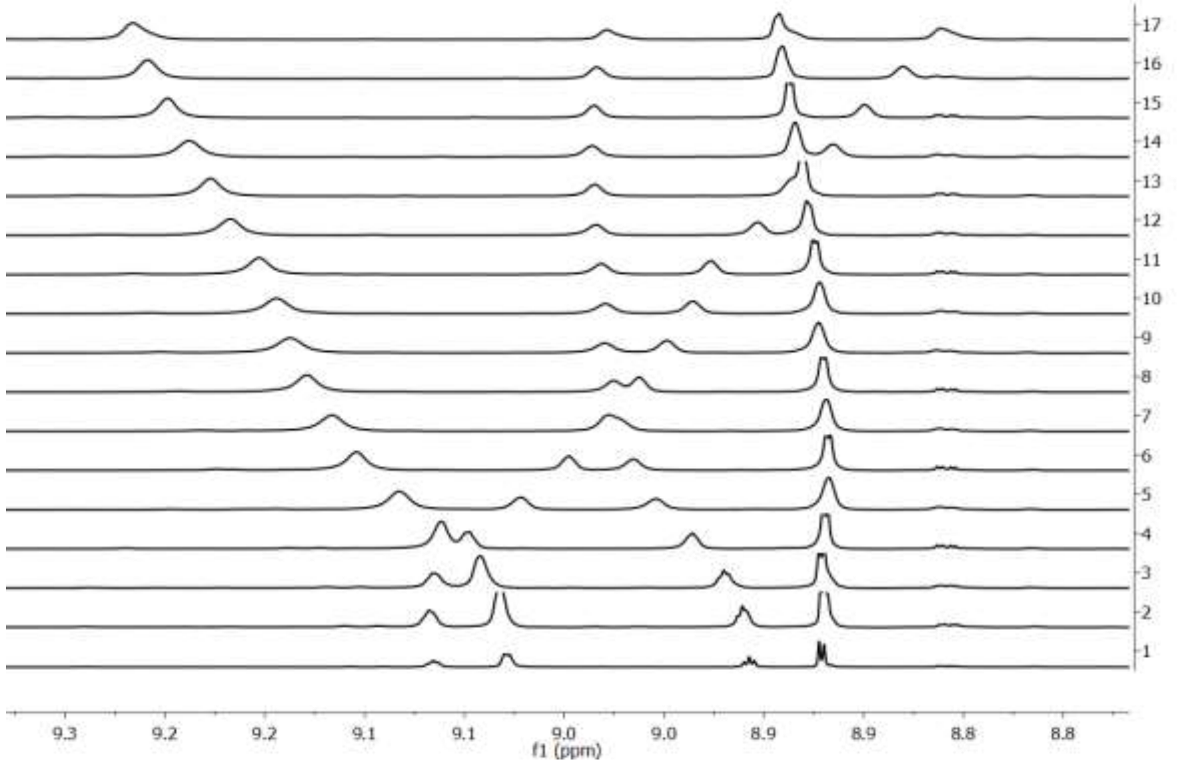

Figure S51. *Truncated* [1]*H NMR spectra of* ***5·PF$_6$*** *upon addition of increasing equivalents of TBA·H_2PO_4 showing downfield region of spectrum (1:1 $CDCl_3$:CD_3OD, 298 K, 600 MHz).*

2:49:49 D_2O:$CDCl_3$:CD_3OD

4·PF_6/TBA·Cl: K_a = 2011 ± 5 % M^{-1}
http://app.supramolecular.org/bindfit/view/5f84fec0-4a8b-4330-b83f-a295cfc4f6df

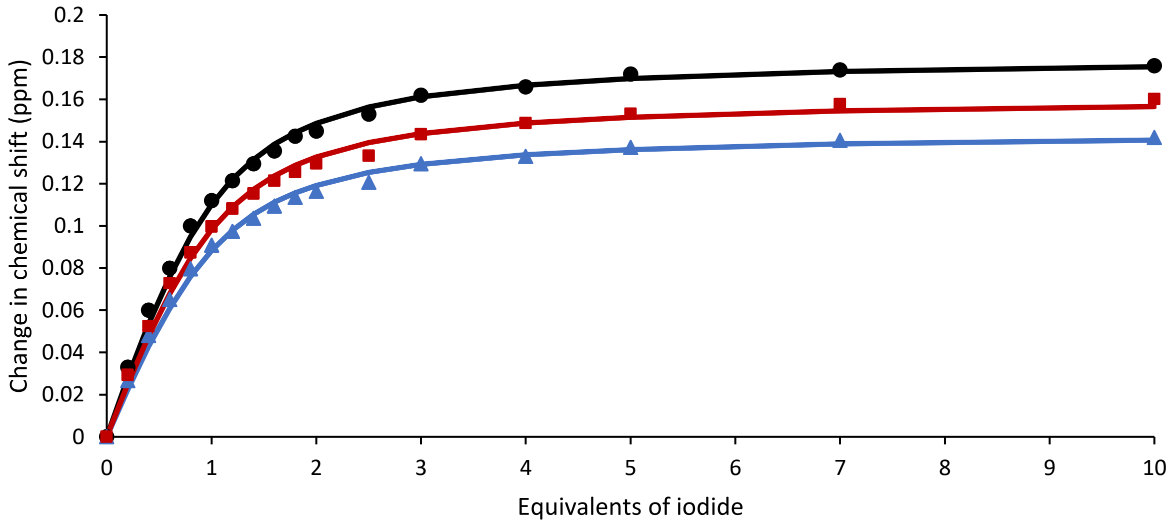

Figure S52. *Movement of peaks at 8.86 ppm (black), 8.78 ppm (blue) and 8.75 ppm (red) upon addition of TBA·Cl (2:49:49 D_2O:$CDCl_3$:CD_3OD, 298 K). Points represent observed data, lines represent 1:1 isotherm calculated using Bindfit.*[9]

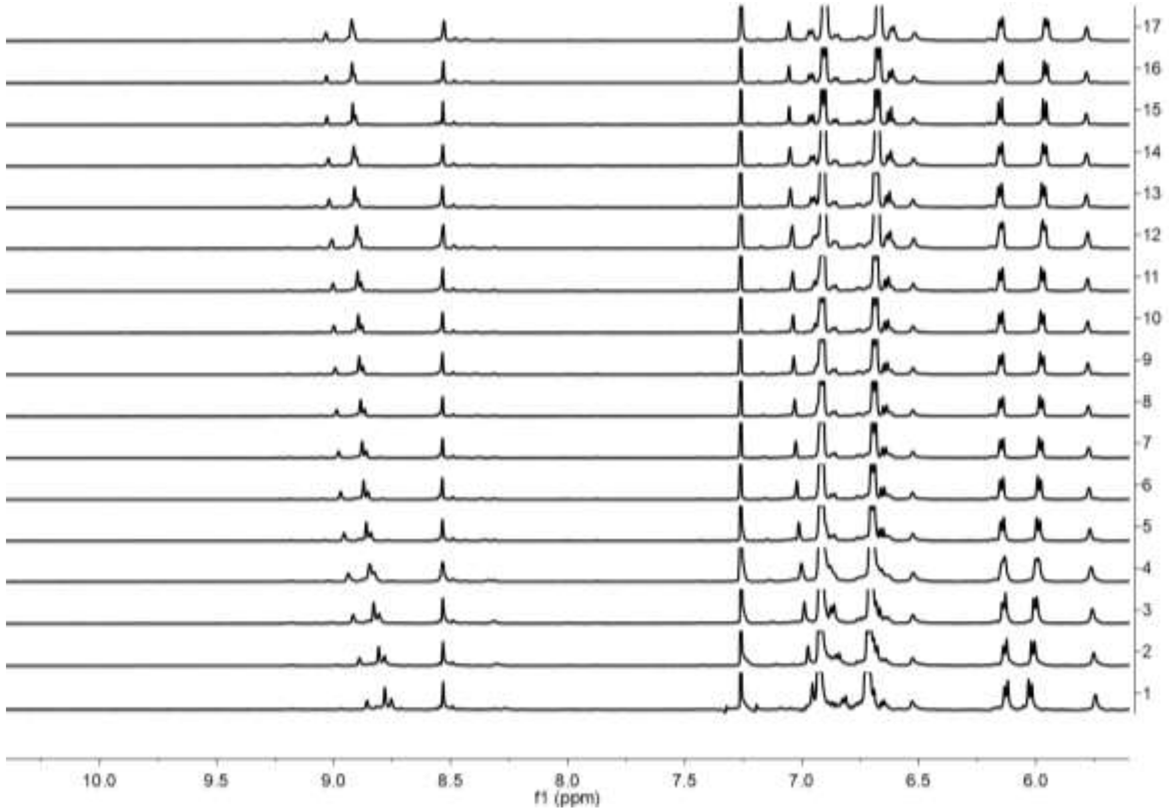

Figure S53. *Truncated ^1H NMR spectra of **4·PF_6** upon addition of increasing equivalents of TBA·Cl (2:49:49 D_2O:$CDCl_3$:CD_3OD, 298 K, 600 MHz).*

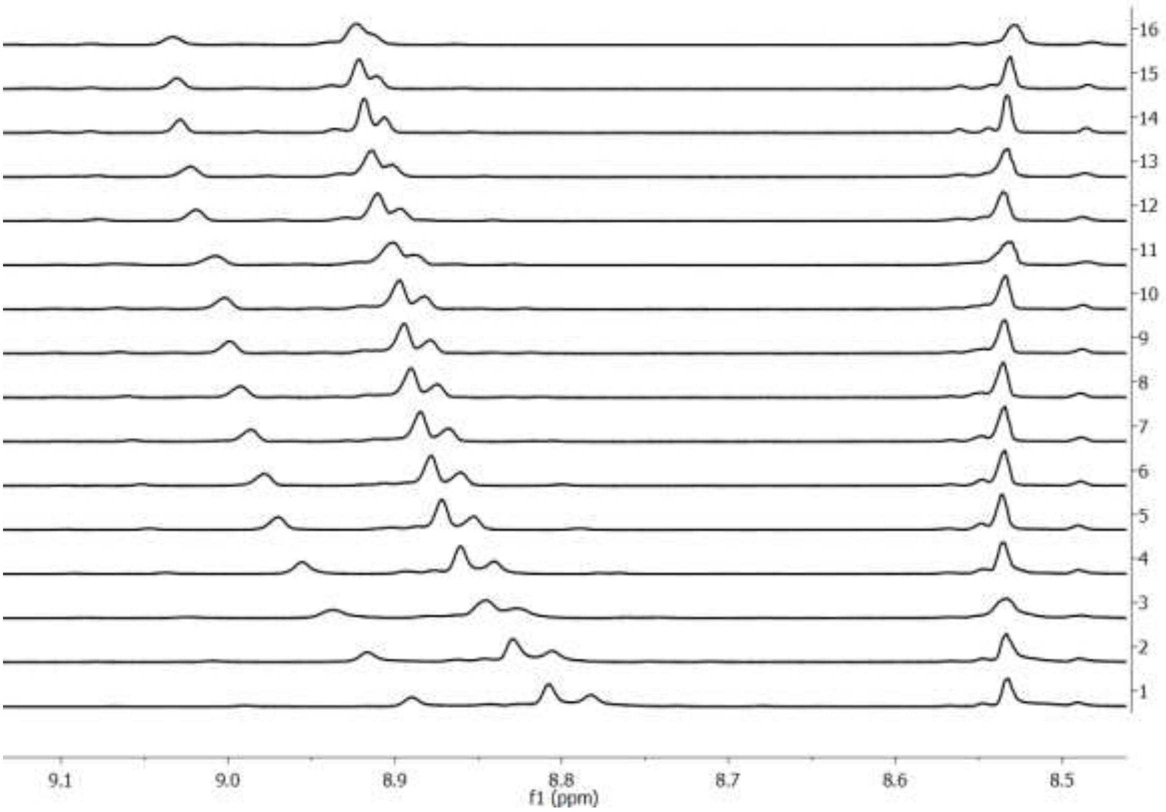

Figure S54. *Truncated ^{1}H NMR spectra of **4·PF$_6$** upon addition of increasing equivalents of TBA·Cl showing downfield region of spectrum (2:49:49 D_2O:$CDCl_3$:CD_3OD, 298 K, 600 MHz).*

1·PF$_6$/TBA·Cl: K_a = 1857 ± 8% M^{-1}
http://app.supramolecular.org/bindfit/view/d991b1d7-6138-4fc3-936f-8092bfaa30ef

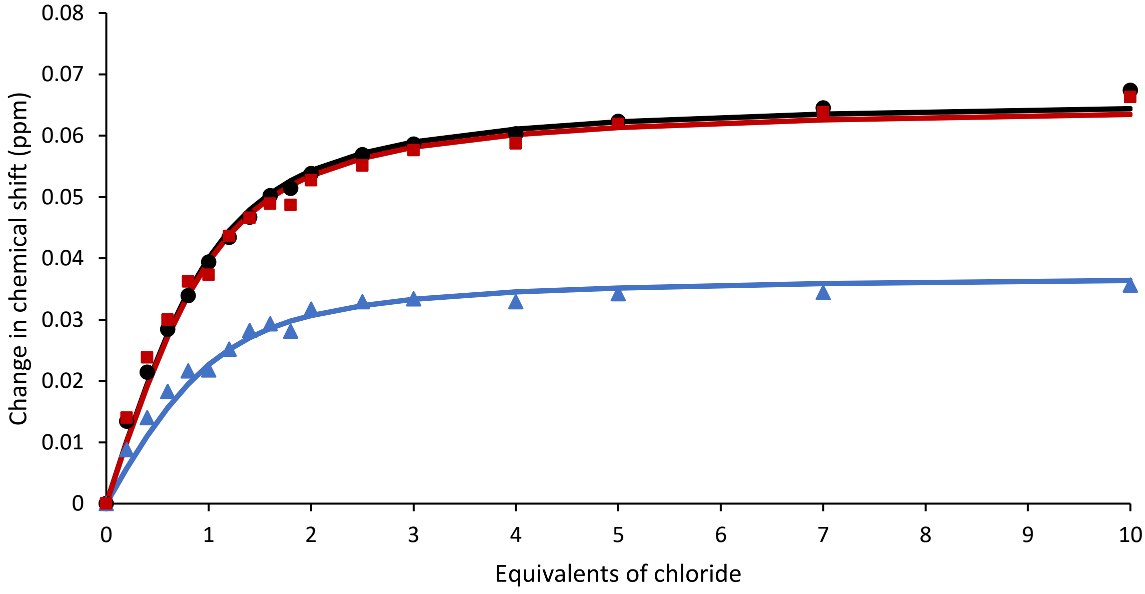

Figure S55. *Movement of peaks at 9.29 ppm (black), 9.40 ppm (blue) and 9.18 ppm (red) upon addition of TBA·Cl (2:49:49 D_2O:$CDCl_3$:CD_3OD, 298 K). Points represent observed data, lines represent 1:1 isotherm calculated using Bindfit.*[9]

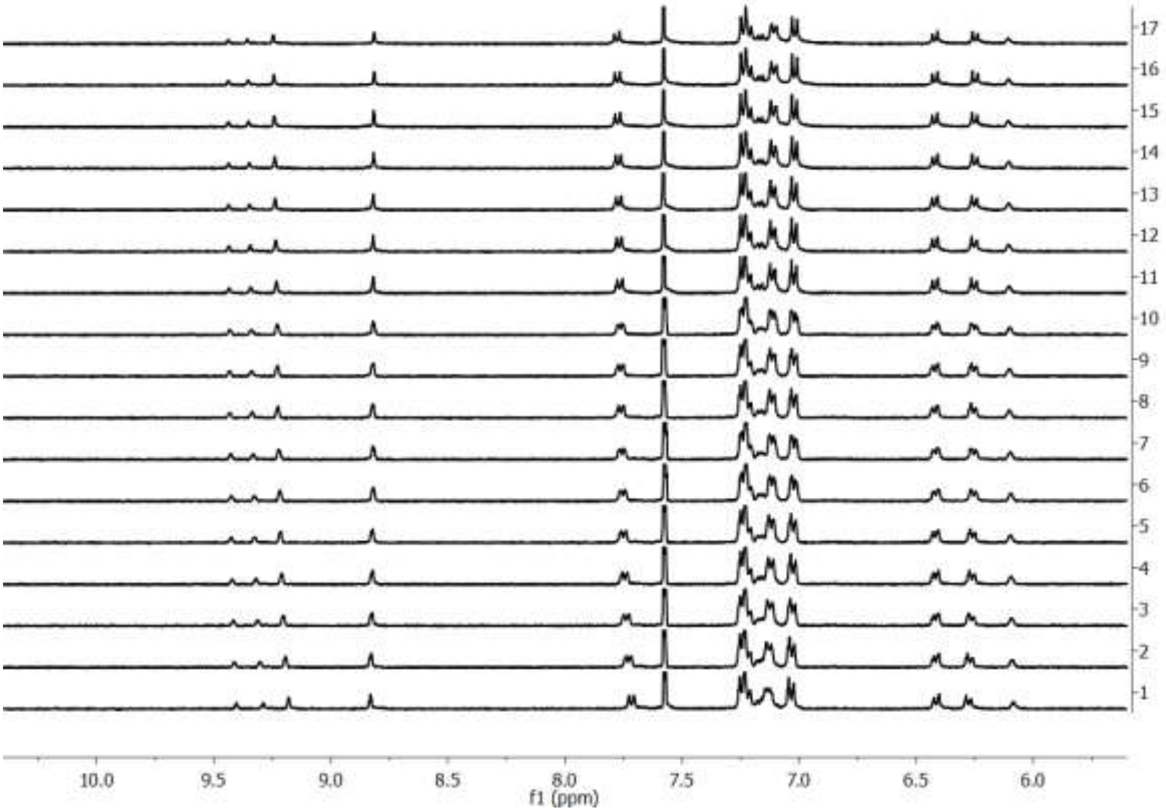

Figure S56. *Truncated* ^{1}*H NMR spectra of* ***1·PF₆*** *upon addition of increasing equivalents of TBA·Cl (2:49:49* D_2O:$CDCl_3$:CD_3OD*, 298 K, 400 MHz).*

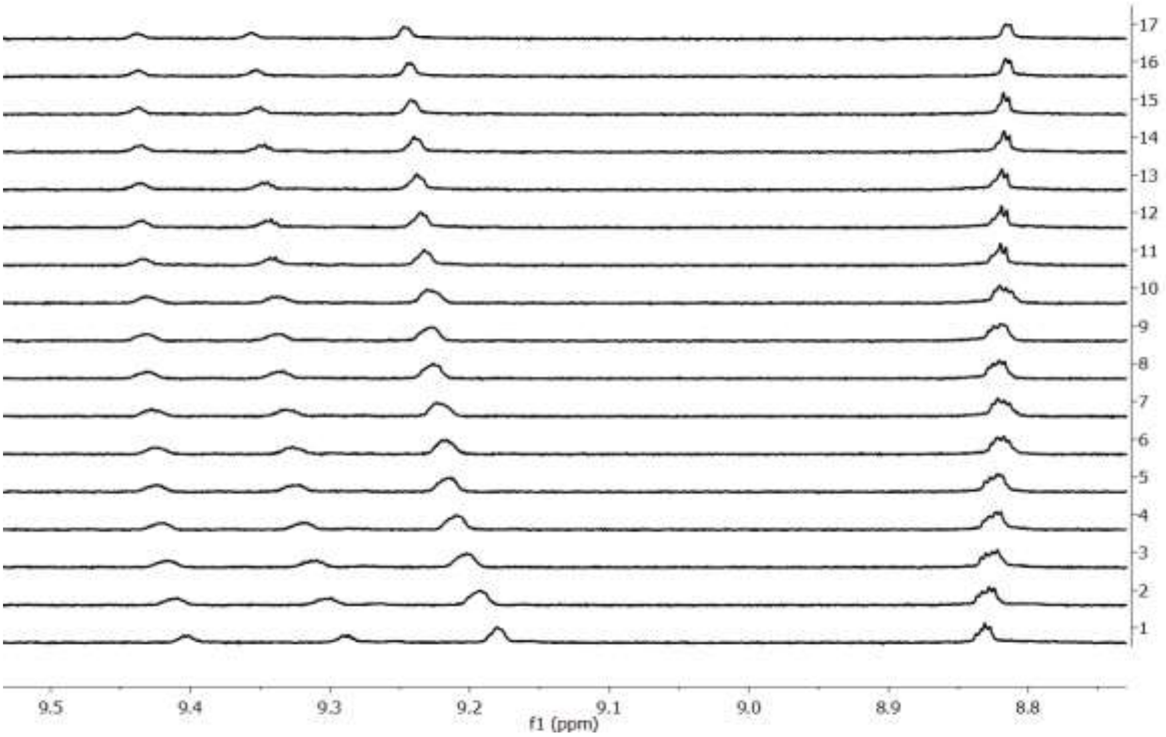

Figure S57. *Truncated* ^{1}*H NMR spectra of* ***1·PF₆*** *upon addition of increasing equivalents of TBA·Cl showing downfield region of spectrum (2:49:49* D_2O:$CDCl_3$:CD_3OD*, 298 K, 400 MHz).*

Single crystal X-ray diffraction data

Data collection
Data for **15** and **5·Cl** were collected on the MX1 beamline[10] at the Australian Synchrotron at 100 K. Raw frame data (including data reduction, interframe scaling and unit cell refinement) were processed using XDS.[11] Data for **4·Cl** were collected on an Oxford Diffraction SuperNova diffractometer using Cu radiation at 150 K. Raw frame data were processed using CrysAlis Pro.[12]

Data refinement
Structures were solved with SUPERFLIP[13] and refined using full-matrix least-squares on F^2 within the CRYSTALS suite.[14] All non-hydrogen atoms were refined with anisotropic displacement parameters. Unless otherwise stated, most C–H hydrogen atoms were visible in the Fourier difference map, and were initially refined with restraints on bond lengths and angles, after which the positions were used as the basis for a riding model.[22] Unless otherwise stated, O–H and N–H hydrogen atoms were visible in the Fourier difference map and were refined with restraints on bond lengths and angles.

Thermal ellipsoid plots
Thermal ellipsoid plots of the crystal structures are shown in Figures S59, S61 and S63. In all cases, ellipsoids are shown at 50% probability levels, and hydrogen atoms are omitted for clarity.

Structure of **15**
Crystals were grown by vapour diffusion of pentane into a dichloromethane solution for the compound. The structure solves and refines in the space group *C2/c*. Apart from restraints on N–H and O–H hydrogen atom positions, it was not necessary to use any crystallographic restraints in the refinement.

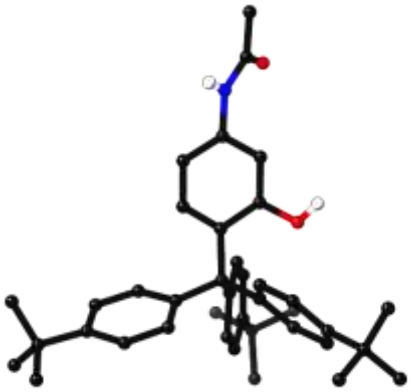

Figure S58. *Single X-ray crystal structure of **15**. Hydrogen atoms omitted for clarity.*

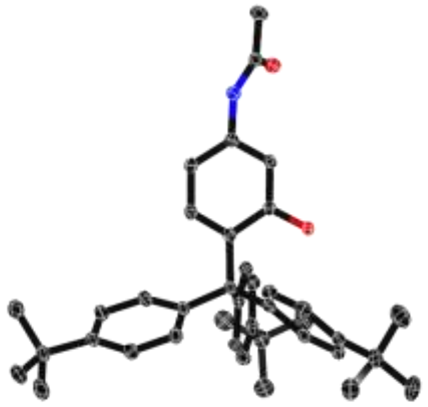

Figure S59. *Thermal ellipsoid plot of* ***15****.*

Structure of **4·Cl**

Crystals were grown by vapour diffusion of diethyl ether into at 1:1 MeOH:DCM solution of the compound. The structure solves and refines in the space group *Cc* and contains one methanol solvent in addition to the rotaxane.

The methanol solvent molecule was disordered over two positions and it was necessary to add restraints to the bond lengths and thermal and vibrational ellipsoid parameters of this molecule as well as to one *t*-butyl group, the nitro group, and a small part of the PEG group of the macrocycle in order to achieve a sensible refinement. O–H and N–H hydrogen atoms were added at idealised hydrogen bonding positions and these positions used as the basis for a riding model.

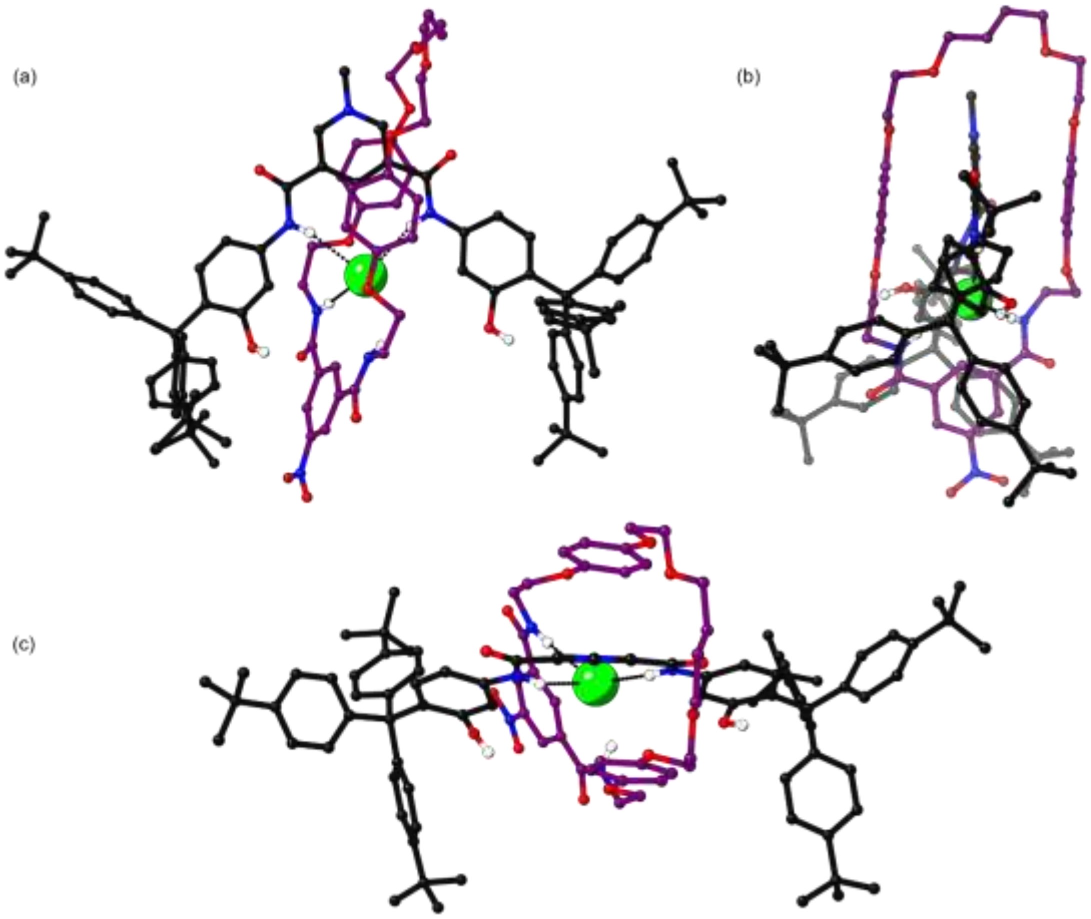

Figure S60. *Different views of the single X-ray crystal structure of **4·Cl**. Most hydrogen atoms and solvent molecules are omitted for clarity. Colour key: axle carbon = black; macrocycle carbon = purple; hydrogen = white; nitrogen = blue; oxygen = red; chloride = green.*

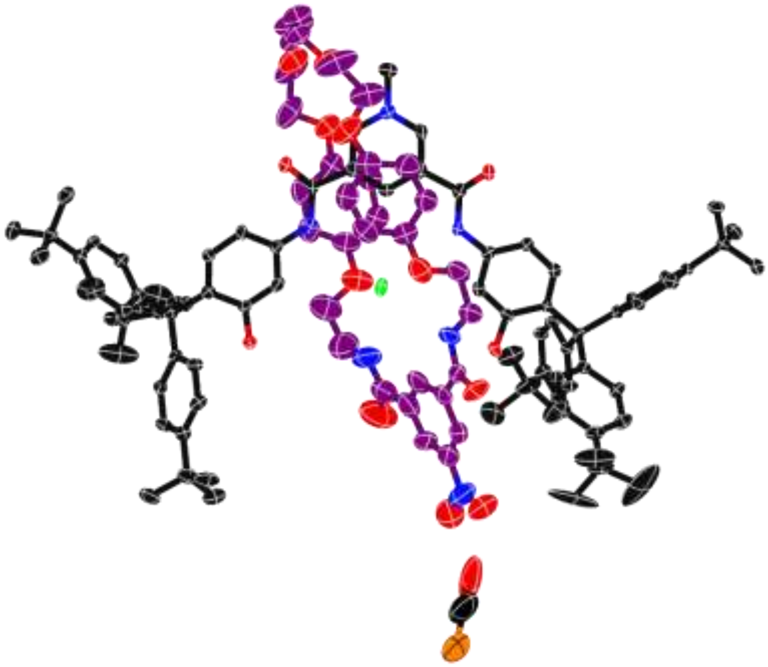

Figure S61. *Thermal ellipsoid plot of **4·Cl**. Disordered solvent molecule included with second position of oxygen atom shown in orange.*

Structure of **5·Cl**

The structure solves and refines in the space group *Cc*. It was necessary to add restarints to the bond length of one of the NH–CH_2–CH_2–O groups of the macrocycle. Extinction was observed within the data set and so an extinction correction was implemented within Crystals. C-H hydrogen atoms were inserted at idealised geometric positions, while O–H and N–H hydrogen atoms were added manually at idealised hydrogen bonding positions. These positions were then used as the basis for a riding model.

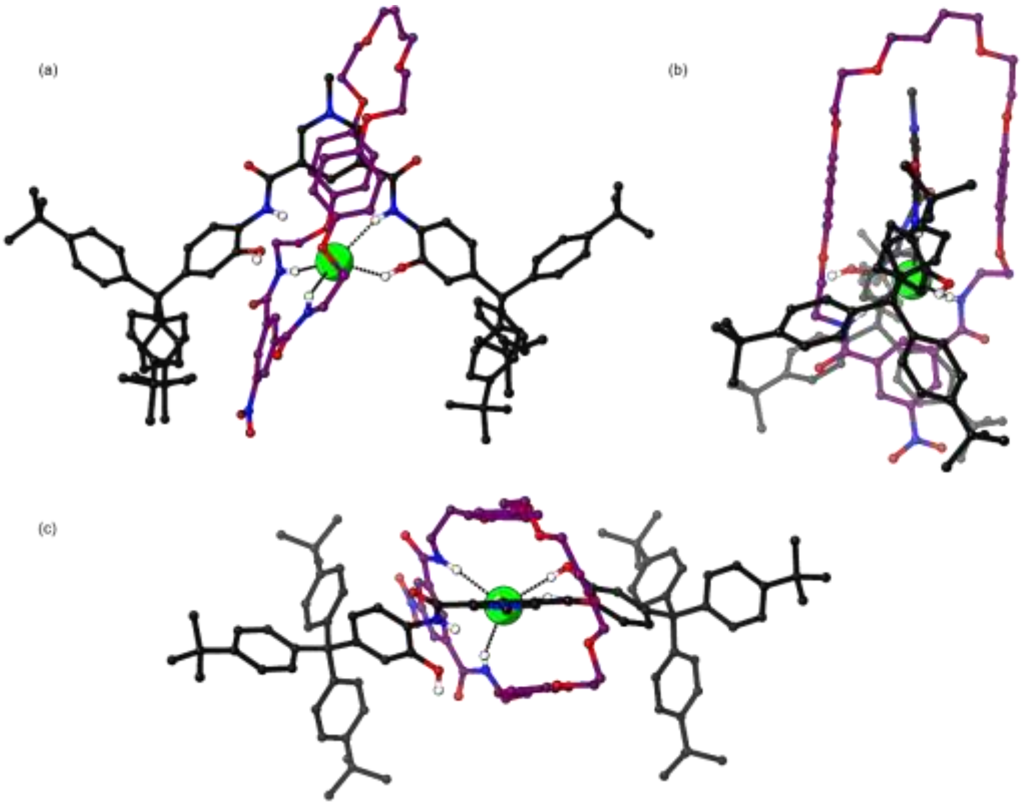

Figure S62. *Different views of the single X-ray crystal structure of **5·Cl**. Most hydrogen atoms and solvent molecules are omitted for clarity. Colour key: axle carbon = black; macrocycle carbon = purple; solvent carbon = orange; hydrogen = white; nitrogen = blue; oxygen = red; chloride = green.*

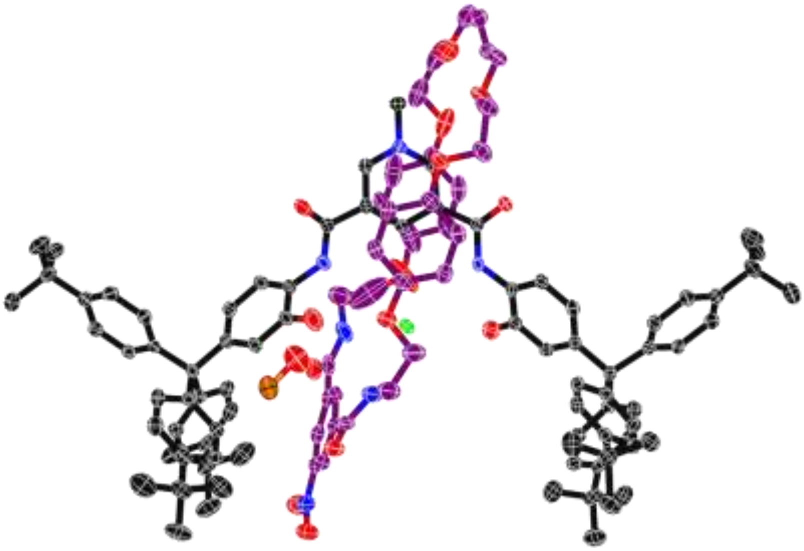

Figure S63. *Thermal ellipsoid plot of **5·Cl**.*

Table of crystallographic data

Table S1. *Selected crystallographic data*

Compound	15	4·Cl	5·Cl
Radiation (wavelength)	synchrotron (0.71073 Å)	Cu Kα (1.54184 Å)	synchrotron (0.71073 Å)
Formula	$C_{39}H_{47}NO_2$	$C_{115}H_{133}ClN_6O_{15}$	$C_{115}H_{135}ClN_6O_{16}$
Formula weight	561.81	1874.81	1892.82
a (Å)	22.09220(7)	18.59430(3)	18.94790(6)
b (Å)	20.00390(6)	14.159640(19)	13.86340(5)
c (Å)	14.87700(5)	38.88574(3)	38.99100(9)
α (˚)	90	90	90
β (˚)	102.404(10)	94.326(3)	98.418(10)
γ (˚)	90	90	90
Unit cell volume (Å^3)	6421.1(2)	10209.0(5)	10131.9(3)
Crystal system	monoclinic	monoclinic	monoclinic
Space group	*C2/c*	*Cc*	*Cc*
Z	8	4	4
Reflections (all)	42194	30344	67652
Reflections (unique)	7074	9317	10233
R_{int}	0.080	0.047	0.132
R_1 [$I > 2\sigma(I)$]	0.0479	0.0716	0.0830
$wR_2(F^2)$ (all data)	0.1248	0.2027	0.2243
CCDC number	2150002	2150001	2150003

Computational simulations

Simulation details

All simulations were conducted using quantum mechanical extended tight binding method GFN2-xTB 6.4.0,[15] as implemented in the *xtb* code, in triplicate. The initial geometry of **1·Cl** was taken from the previously reported X-ray crystal structure (CCDC: 187740), the initial geometries of **4·Cl** and **5·Cl** were taken from the X-ray crystal structures reported in this work. For simulations with acetate and hexafluorophosphate anions IQMOL 2.11[16] was used to add the anions into the crystal structure in a position within 3.0 Å of the binding pocket. These were then energy minimized with the MMFF forcefield in IQMOL 2.11 and this pre-optimized geometry was used as the starting geometry (Figures S74-S82). All GFN2-xTB molecular dynamics (MD) calculations were performed with initial "loose" optimization [loose: E_{conv} (energy convergence) = 5×10^{-5} E_{h}; G_{conv} (gradient convergence) = 4×10^{-3} $E_{\text{h}}\cdot\alpha^{-1}$; accuracy (for integral cutoffs and SCF criteria) = 2.00] and the solvent methanol was represented with an implicit solvation model (ALPB, as implemented in the xTB code).[17] Lebedev grid level used was "normal" with 230 grid points (as implemented in the xTB code).[18] The simulations were conducted in the NVT ensemble with the system temperature being maintained at 298.15 K. All bonds were constrained using the SHAKE algorithm.[19] Each simulation was run for 5000 ps, with trajectory output every 150 fs, with a propagation time step of 4 fs.

Each simulation contained either **1⁺**, **4⁺** and **5⁺** and one chloride, acetate or hexafluorophosphate anion in implicit methanol.

Analysis details

The conformer ensemble sampling program CREST[20] was used to sort the given trajectories according to energy, RMSD and rotational constant. An energy window of 10 kcal/mol was used. To determine the lowest energy structures the first 90 frames were removed from the data set so as to remove the initial loose optimization. The lowest energy conformers were then used to inform the analysis and identify binding modes that were the most stable.

Time trace bond lengths were measured in VMD[21] and images were prepared in CrystalMaker®.[22] Non-covalent interactions were measured from the hydrogen atom to the hydrogen bond acceptor, with hydrogen bonding events between the rotaxanes and chloride anion being defined as any interaction <3.02 Å (the sum of the van der Waals radii of hydrogen and chlorine[23]), with the angle of the interaction not being considered. For simulations with an acetate anion, the hydrogen bonding length was approximated to <3.70 Å due to the necessity to measure to the central acetate carbon (Figure S75 C1, S78 C1 and S81 C1). Again, the first 90 dataset points were not included in the time traces to remove the initial optimization conformers. Within the graphs, all of the data points are included as a transparent scatterplot and then a moving average, with an interval of 100 data points, is represented as an opaque scatterplot.

Simulation 1 for **5·Cl** encountered unbinding issues, where the chloride anion unbound from the rotaxane and rapidly distanced itself from the rotaxane as the *xtb* code lacks periodic boundary conditions. This occurred after 1800 ps, and as such the data used for the time trace and percentage binding time data excluded the data from 1700 ps to 5000 ps.

Simulation 1 for **5·OAc** failed to run for the intended time, instead finishing at 3880 ps. This was due to an internal bug within the program where it failed to restart. Despite the reduced run time, it was deemed that there was enough data to determine the most stable conformer as well as the main binding modes.

The various intercomponent interactions between the macrocycle and axle were identified through the generated CREST conformers. These interactions were then analysed over the entirety of the simulation, with the five most persistent interactions being graphed as time traces (Figures S83-S148). Within the graphs, all of the data points are included as a transparent scatterplot and then a moving average, with an interval of 100 data points, is represented as an opaque scatterplot.

The distance between the interior pyridinium proton (CH) to the centre of the anion was used to approximate the percentage of time that the chloride or acetate anion was in the cavity of the rotaxanes. Again, when determining the percentage of binding time between the hydrogen bond donors and the interior pyridinium proton, the first 90 dataset points were not included in the time traces so as to remove the initial optimization conformers.

*CREST data analysis of **1·Cl**, **4·Cl** and **5·Cl***

CREST data of **1·Cl** and **4·Cl** indicated that there were three main most stable binding modes, with either one, two or three amides binding to the chloride anion. Within the **1·Cl** conformers, other secondary inter-component interactions were also observed between non-anion bound amides and either a carbonyl oxygen (Figure S64a and S64d) or glycol oxygen (Figure S64b and S64c). These lowest energy structures were consistent with the binding interactions that were observed over the duration of the simulations (Table S2, Figures S83-S88).

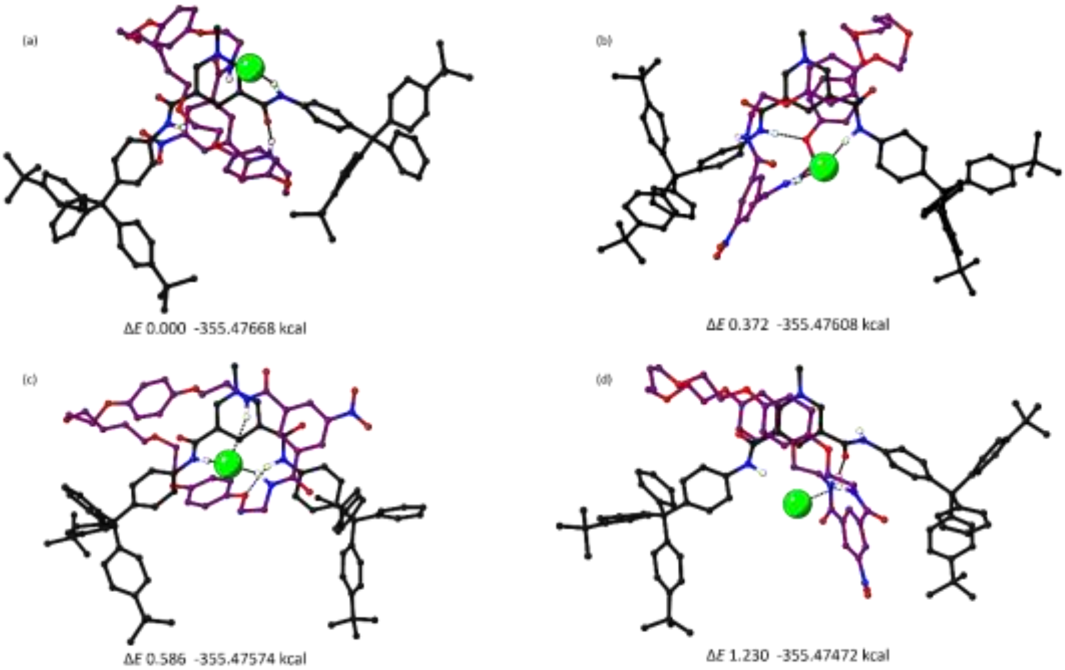

Figure S64. *Representative structures from CREST sampling program; (a)* ***1·Cl*** *with anion bound outside of the cavity with one N−H···anion hydrogen bond; (b)* ***1·Cl*** *with anion bound inside the cavity with two N−H···anion hydrogen bonds; (c)* ***1·Cl*** *with anion bound inside the cavity with three N−H···anion hydrogen bonds; (d)* ***1·Cl*** *with anion bound inside the cavity with one N−H···anion hydrogen bonds; dotted black lines indicate hydrogen bond interactions (the*

sum of the van der Waal radii). Most hydrogen atoms omitted for clarity. All ΔE energies are relative to the most stable conformer (a).

These inter-component binding events were also identified within the **4·Cl** simulations between non-anion bound amides and either a carbonyl oxygen (Figure S65a) or hydroxy oxygen (Figure S65c). As stated in the main text, hydrogen bonding between the hydroxy groups and chloride anion was rarely observed (Table S2), the hydroxy groups either interacting with the macrocycle amides or carbonyl oxygens (Figures S65b and S65c), or they interacted with the implicit solvent. These lowest energy structures are consistent with the binding interactions that were observed over the duration of the simulations (Table S2, Figures S101-S109).

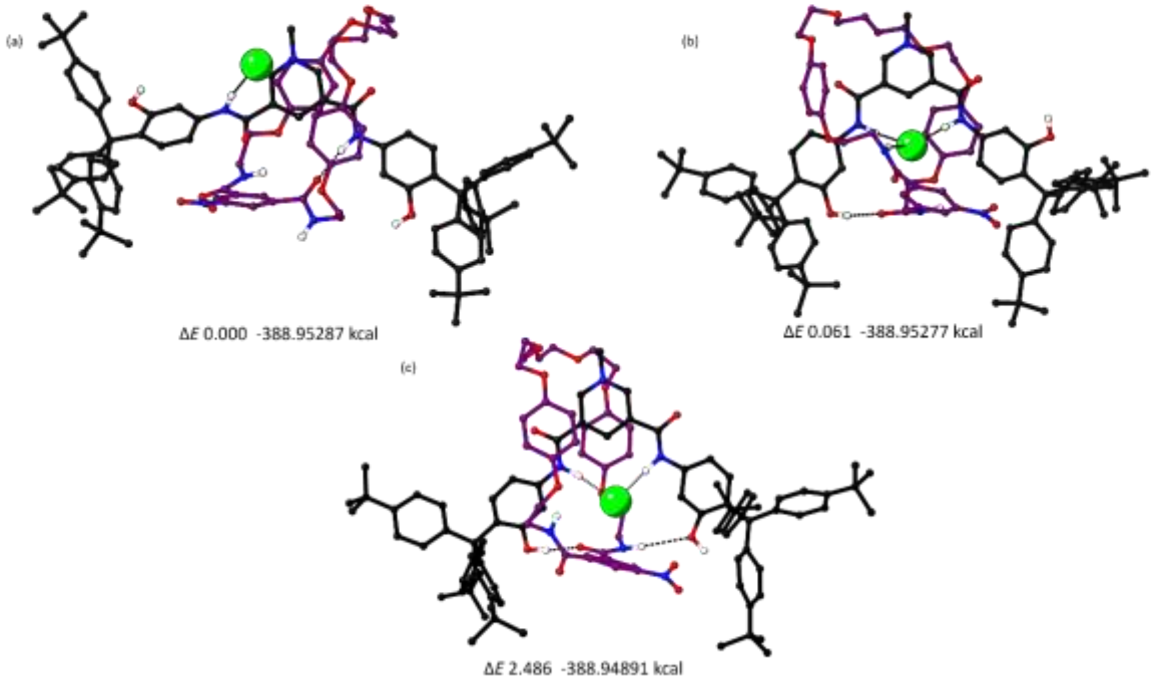

Figure S65. *Representative structures from CREST sampling program; (a)* ***4·Cl*** *with anion bound outside of the cavity with one N−H···anion hydrogen bond; (b)* ***4·Cl*** *with anion bound inside the cavity with three N−H···anion hydrogen bonds; (c)* ***4·Cl*** *with anion bound inside the cavity with two N−H···anion hydrogen bonds; dotted black lines indicate hydrogen bond interactions (the sum of the van der Waal radii). Most hydrogen atoms omitted for clarity. All ΔE energies are relative to the most stable conformer (a).*

As highlighted in Table S2 and the following graphs (Figures S125-S133), the most stable **5·Cl** conformers had one amide and one hydroxy group hydrogen bonding to the chloride anion. This binding arrangement was routinely observed over the duration of the simulations (Figure S66a). While one hydroxy was hydrogen bonding to the chloride anion, often the other hydroxy group would hydrogen bond to the macrocycle carbonyl oxygen (Figure S66b).

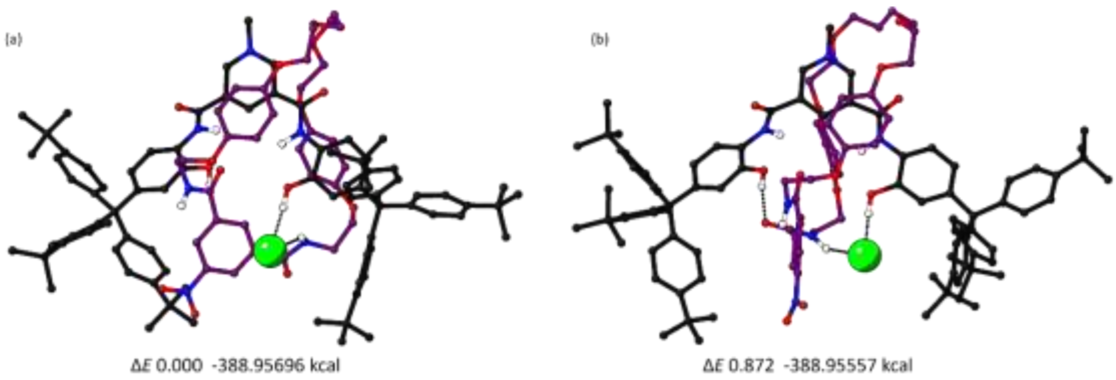

Figure S66. *Representative structures of **5·Cl** from CREST sampling program with anion bound on the exterior of the rotaxane cavity with one N−H···anion hydrogen bond and one O−H···anion hydrogen bond; dotted black lines indicate hydrogen bond interactions (the sum of the van der Waal radii). Most hydrogen atoms omitted for clarity. All ΔE energies are relative to the most stable conformer (a).*

*CREST data analysis of **1·OAc**, **4·OAc** and **5·OAc***

CREST data of **1·OAc** simulations indicated that the most stable conformers contained the acetate anion within the interior of the rotaxane with either two or four N−H···anion hydrogen bonds. As indicated in Table S2, over the duration of the simulations there appeared to be a preference for two N−H···anion hydrogen bonds rather than four, despite Figure S67a being the most stable conformer. This may be due to the steric constraints of the rotaxane interior binding pocket (Figure S67).

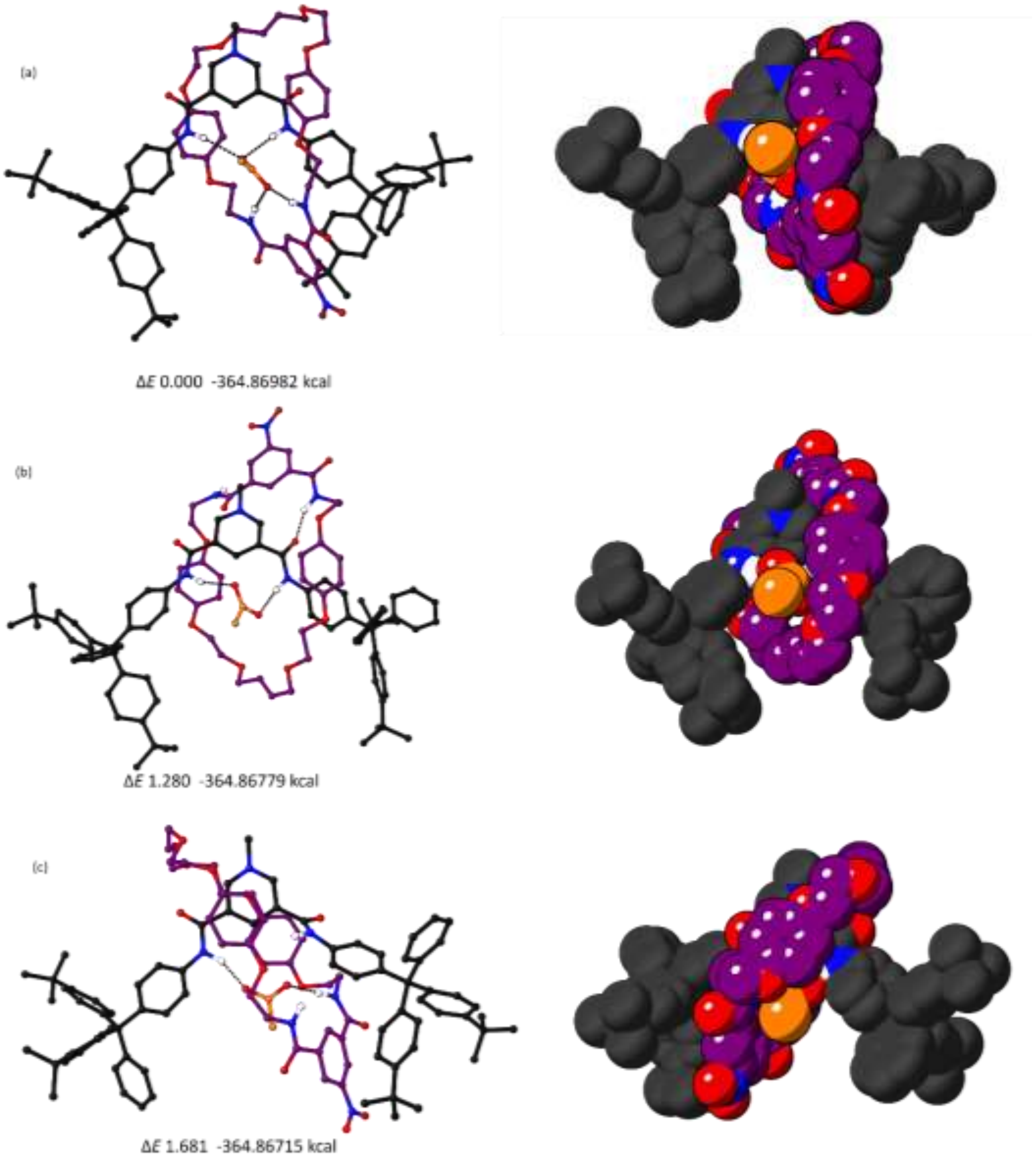

Figure S67. *Representative structures from CREST sampling program in both ball and stick and VDW representation; (a)* ***1·OAc*** *with anion bound inside of the cavity with four N−H···anion hydrogen bonds; (b)* ***1·OAc*** *with anion bound inside the cavity with two N−H···anion hydrogen bonds; (c)* ***1·OAc*** *with anion bound inside the cavity with two N−H···anion hydrogen bonds; dotted black lines indicate hydrogen bond interactions (the sum of the van der Waal radii). Most hydrogen atoms omitted for clarity, acetate anion carbon shown in orange. All ΔE energies are relative to the most stable conformer (a).*

Similar to **1·OAc**, the most stable CREST conformers for **4·OAc** indicated that three N−H···anion hydrogen bonds generated the lowest energy structures (Figure S68a and S68b). However, over the duration of the simulations, it was found that there was a slight preference towards conformers with one amide and one hydroxy group hydrogen bonding to the acetate anion on the exterior of the rotaxane while the axle amides hydrogen bonded to the macrocycle carbonyl oxygens (Figure S68c; Table S3). This may be due to steric constraints, with the acetate anion ‘perching’ out of the binding pocket (Figure S69). Intercomponent interactions between the hydroxy groups and the macrocycle amides and carbonyl oxygens

were again observed, potentially reducing the favourability of the anion residing within the pocket, as discussed in the main text.

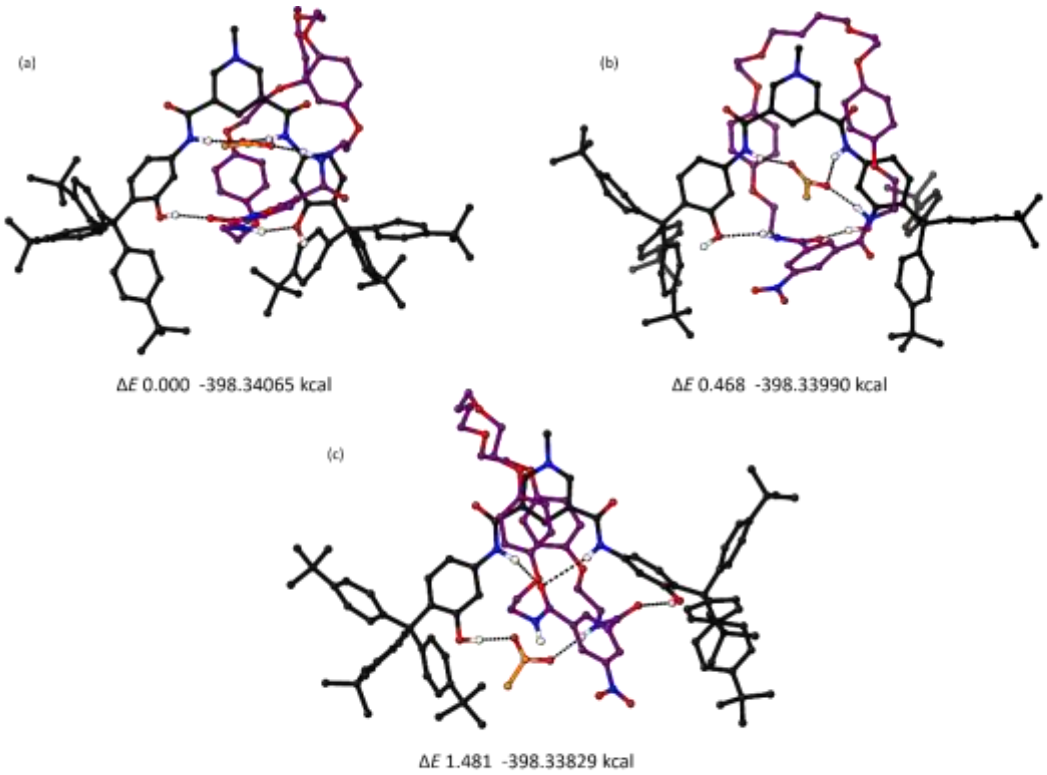

Figure S68. *Representative structures from CREST sampling program; (a)* ***4·OAc*** *with anion bound inside of the cavity with three N−H···anion hydrogen bonds; (b)* ***4·OAc*** *with anion bound inside the cavity with three N−H···anion hydrogen bonds; (c)* ***4·OAc*** *with anion bound outside of the cavity with one N−H···anion hydrogen bond and one O−H···anion hydrogen bond; dotted black lines indicate hydrogen bond interactions (the sum of the van der Waal radii). Most hydrogen atoms omitted for clarity; acetate anion carbon shown in orange. All ΔE energies are relative to the most stable conformer (a).*

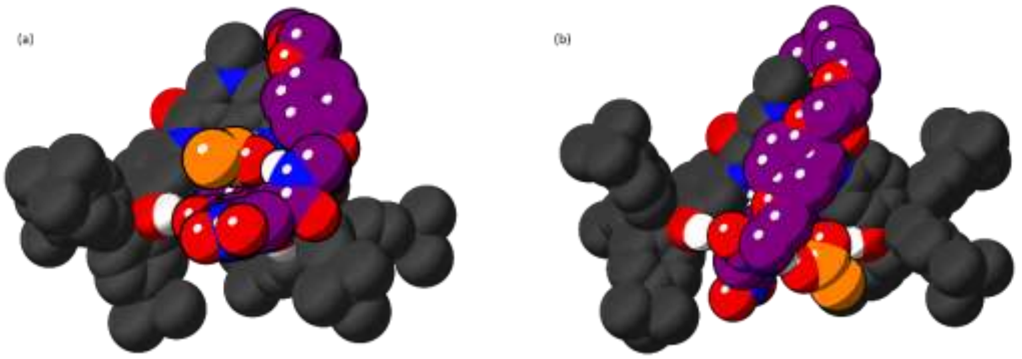

Figure S69. *Representative structures from CREST sampling program; (a)* ***4·OAc*** *with anion bound inside of the cavity; (b)* ***4·OAc*** *with anion bound outside of the cavity; most hydrogen atoms omitted for clarity, acetate anion carbon shown in orange.*

CREST data for the simulations of **5·OAc** indicated that, similar to **5·Cl**, the most stable conformers bound the acetate anion on the exterior of the rotaxane, with one amide hydrogen bond and one hydroxy hydrogen bond (Figure S70). As seen in **4·OAc** simulations, if the acetate left the interior binding pocket, the axle amides would bind with the macrocycle

carbonyl oxygens within the binding cavity, effectively competing for the interior binding pocket. Competitive intramolecular hydrogen bonding between the hydroxy groups and axle carbonyl oxygen was also observed, consistent with literature results.[24]

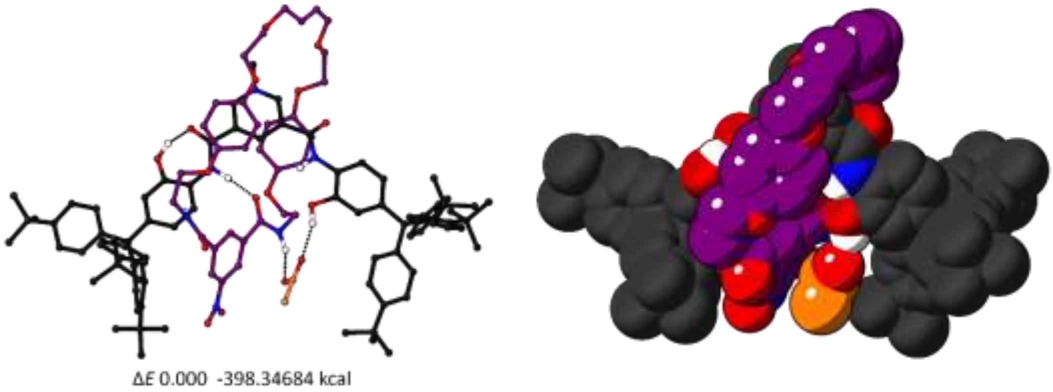

Figure S70. *Lowest energy structure of* ***5·OAc*** *from CREST sampling program in ball and stick and VDW representation with anion bound on the exterior of the rotaxane cavity with one N−H···anion hydrogen bond and one O−H···anion hydrogen bond; dotted black lines indicate hydrogen bond interactions (the sum of the van der Waal radii). Most hydrogen atoms omitted for clarity, acetate anion carbon shown in orange.*

Table S2. *Percentage binding time between rotaxane hydrogen bond donors and chloride anion.*

[2]Rotaxane·anion	Total cavity binding time	Binding to 4 amide groups	Binding to 3 amide groups	Binding to 2 amide groups	Binding to 1 amide group	Binding to 2 hydroxy groups	Binding to 1 hydroxy group
1·Cl	57%	7%	21%	30%	41%	-	-
4·Cl	48%	1%	17%	17%	47%	0%	0.2%
5·Cl	16%	0%	0.2%	14%	33%	0%	83%

Table S3. *Percentage binding time between rotaxane hydrogen bond donors and acetate anion.*

[2]Rotaxane.anion	Total cavity binding time	Binding to 4 amide groups	Binding to 3 amide groups	Binding to 2 amide groups	Binding to 1 amide group	Binding to 2 hydroxy groups	Binding to 1 hydroxy group
1·OAc	76%	20%	29%	42%	8%	-	-
4·OAc	44%	6%	28%	20%	40%	0%	41%
5·OAc	31%	1%	10%	35%	50%	10%	89%

*CREST data analysis of **1·PF₆**, **4·PF₆**and **5·PF₆***

In order to study the conformers of **1**$^+$, **4**$^+$ and **5**$^+$ in the presence of a noncoordinating anion, simulations were conducted with the PF_6^- anion. By doing this, it was possible to investigate what sort of intercomponent interactions may be occurring within the rotaxanes before the coordination of either a chloride or acetate anion, *i.e.* the preorganisation of the rotaxane.

The CREST data of **1·PF₆** indicated that the most stable conformers again contained intercomponent interactions between the macrocycle and axle, with these interactions drawing the macrocycle close to the axle (Figure S71).

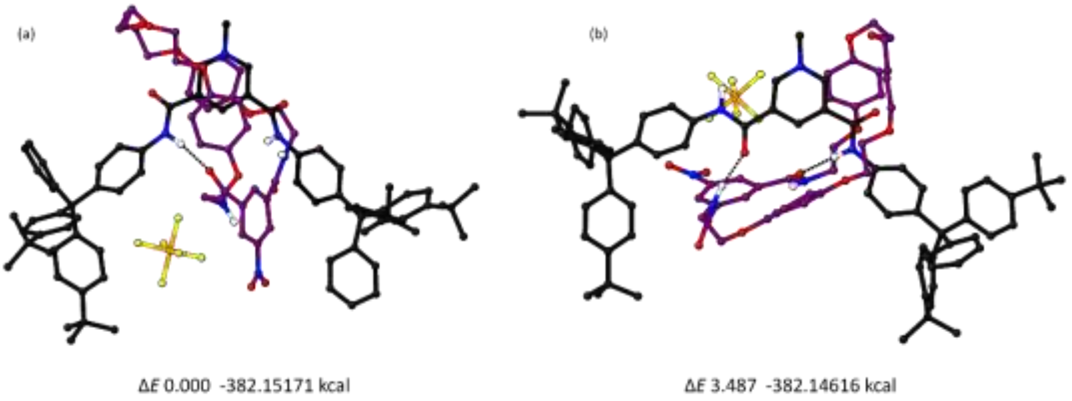

***Figure S71.** Representative structures of **1·PF₆** from CREST sampling program showing intercomponent interactions; dotted black lines indicate hydrogen bond interactions (the sum of the van der Waal radii). Most hydrogen atoms omitted for clarity. All ΔE energies are relative to the most stable conformer (a).*

In the simulations of **4·PF₆**, hydrogen bonding between the macrocycle and axle was again observed, with one of the hydroxy groups interacting with the macrocycle. In contrast to **1·PF₆**, the **4·PF₆** lowest energy conformers appear to have a more open and preorganised structure between the macrocycle and the axle (Figure S72a), potentially due to the hydrogen bonding between the hydroxy group and the nitro oxygen.

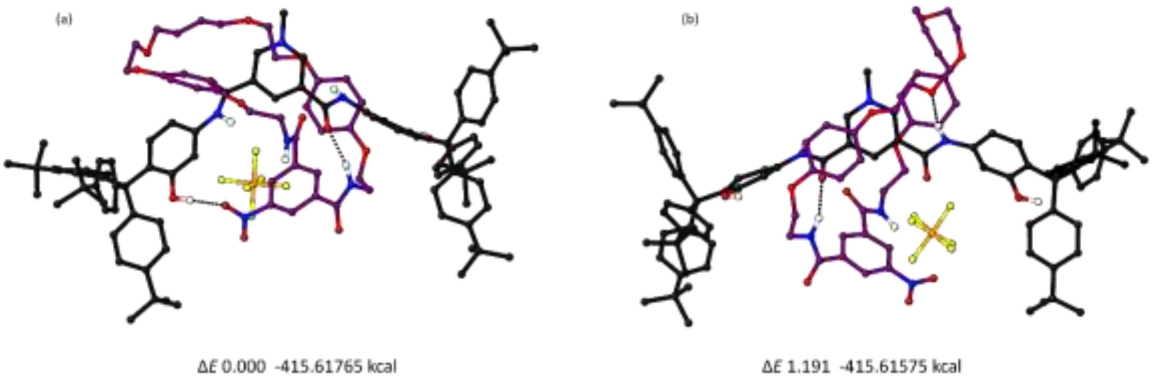

***Figure S72.** Representative structures of **4·PF₆** from CREST sampling program showing intercomponent interactions; dotted black lines indicate hydrogen bond interactions (the sum of the van der Waal radii). Most hydrogen atoms omitted for clarity. All ΔE energies are relative to the most stable conformer (a).*

Similar to conformers observed in simulations of **5·OAc**, **5·PF₆** conformers showed various intercomponent interactions between the macrocycle and axle, with the the axle amides again

hydrogen bonding to the macrocycle carbonyl oxygens within the binding cavity. This appears to again collapse the binding pocket, potentially decreasing the ability for subsequent guest binding (Figure S73).

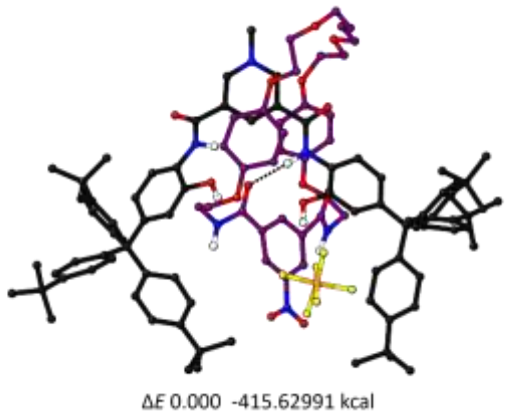

Figure S73. *Lowest energy structure of* ***5·PF$_6$*** *from CREST sampling program showing intercomponent interactions; dotted black lines indicate hydrogen bond interactions (the sum of the van der Waal radii). Most hydrogen atoms omitted for clarity. All ΔE energies are relative to the most stable conformer.*

Starting geometries

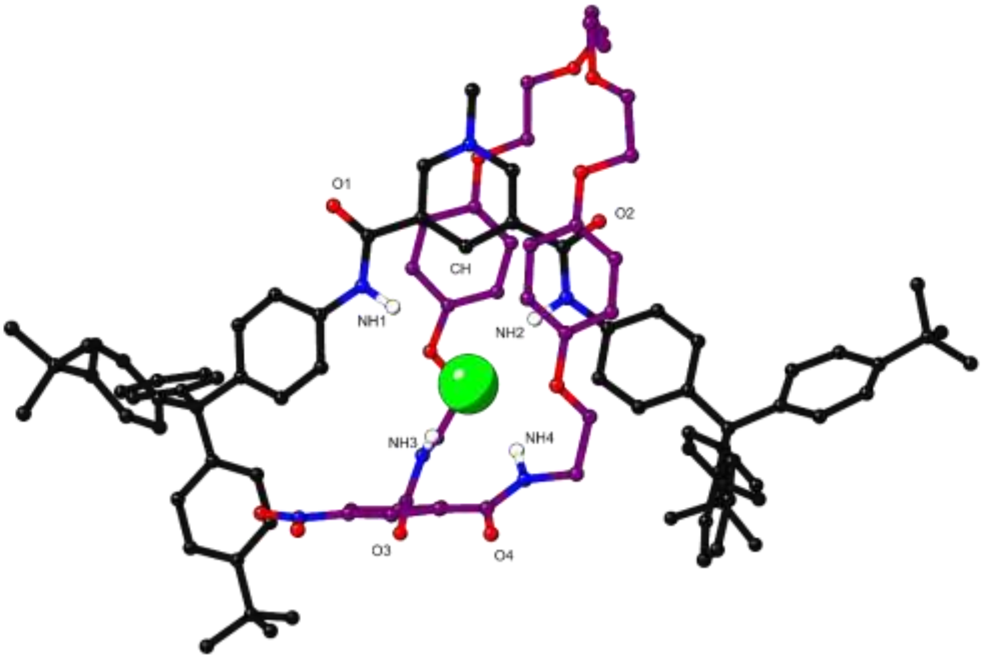

Figure S74. *Starting geometry of **1·Cl**. Atom labels are coordinated with graph legends in Figures S83-S88.*

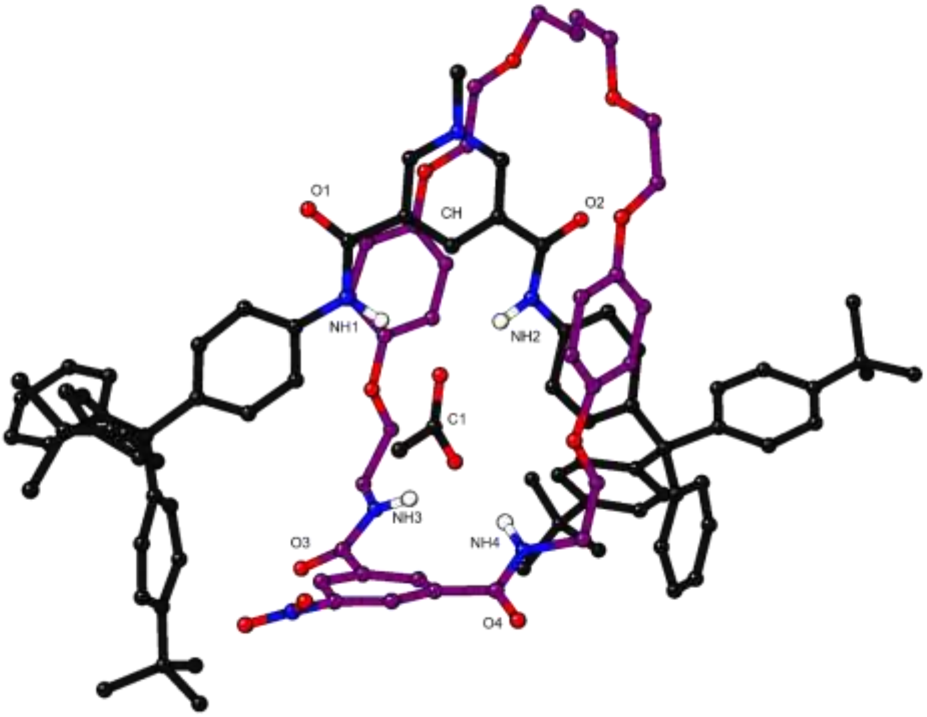

Figure S75. *Starting geometry of **1·OAc**. Atom labels are coordinated with graph legends in Figures S89-S94.*

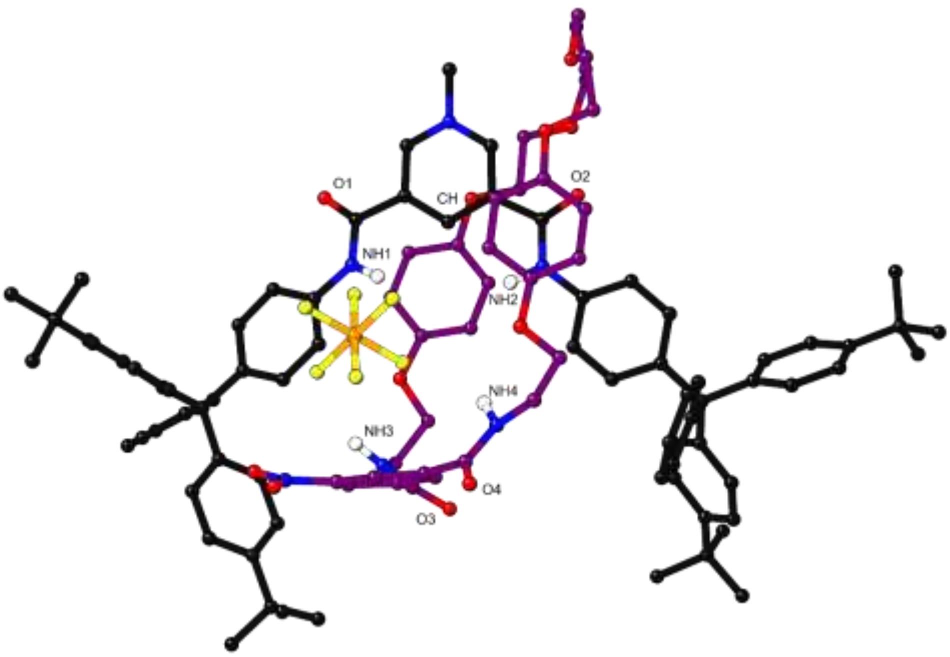

Figure S76. *Starting geometry of **1·PF₆**. Atom labels are coordinated with graph legends in Figures S95-S100.*

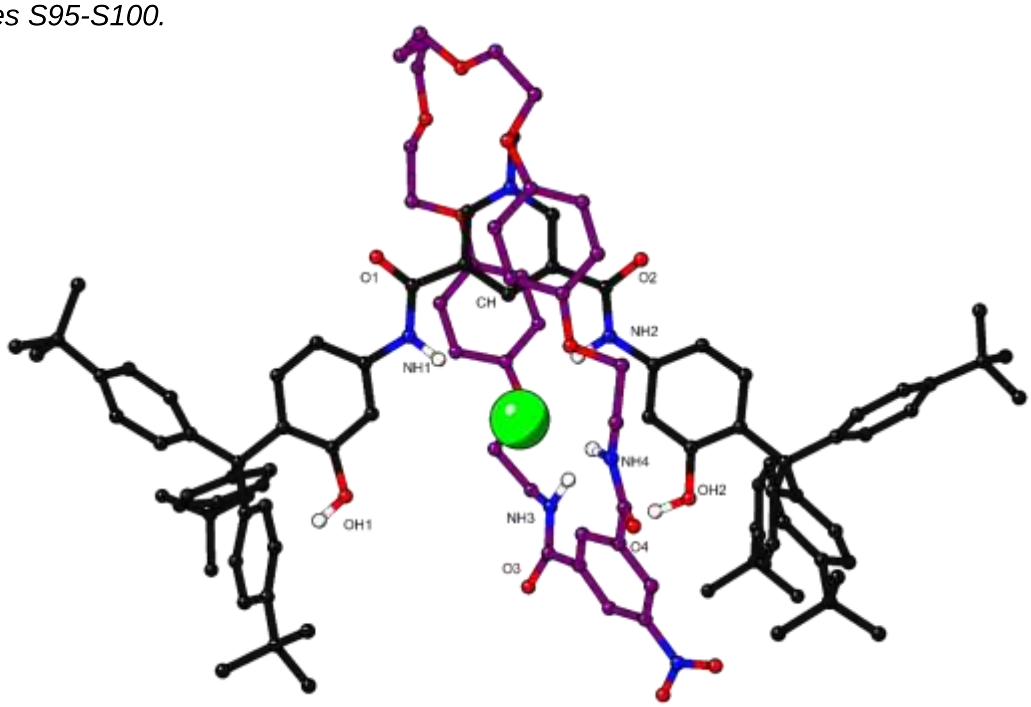

Figure S77. *Starting geometry of **4·Cl**. Atom labels are coordinated with graph legends in Figures S101-S109.*

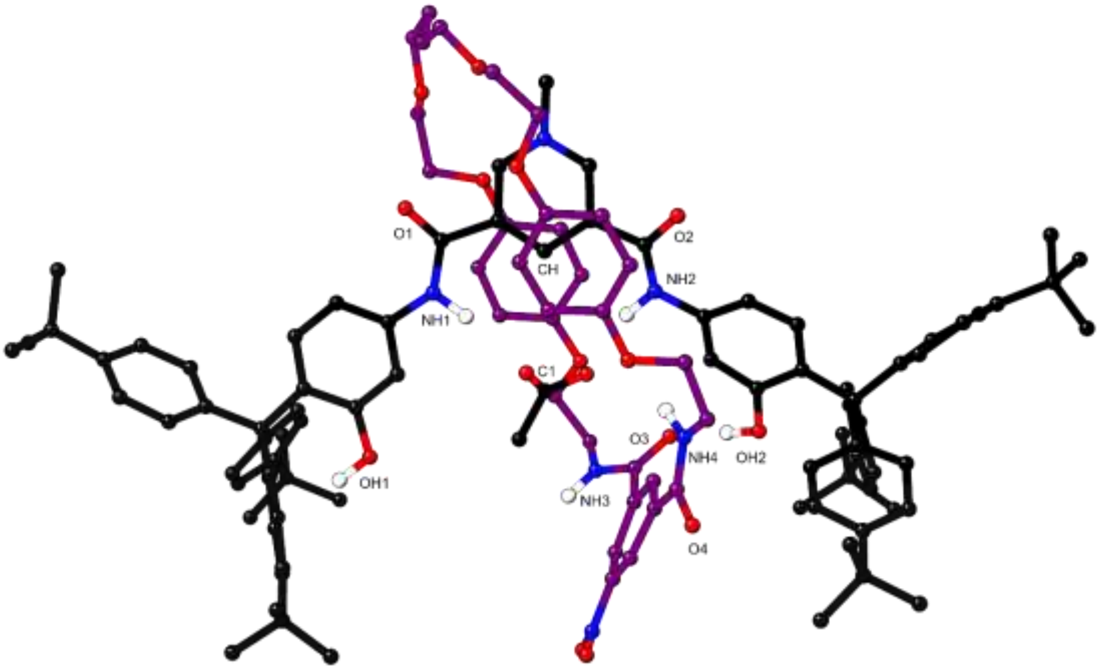

Figure S78. *Starting geometry of* ***4·OAc****. Atom labels are coordinated with graph legends in Figures S110-S118.*

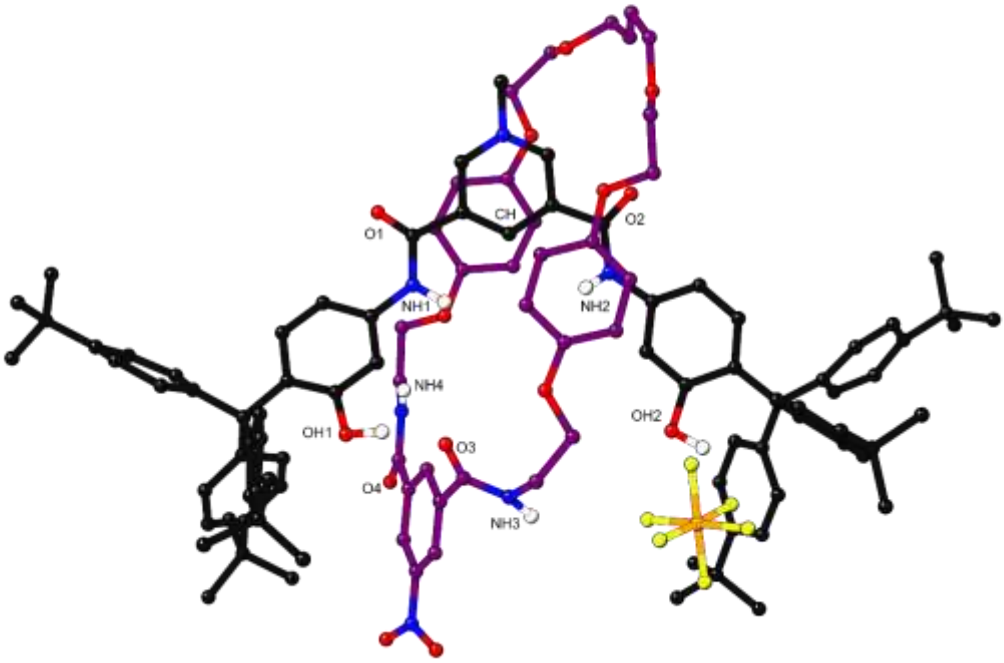

Figure S79. *Starting geometry of* ***4·PF$_6$****. Atom labels are coordinated with graph legends in Figures S119-S124.*

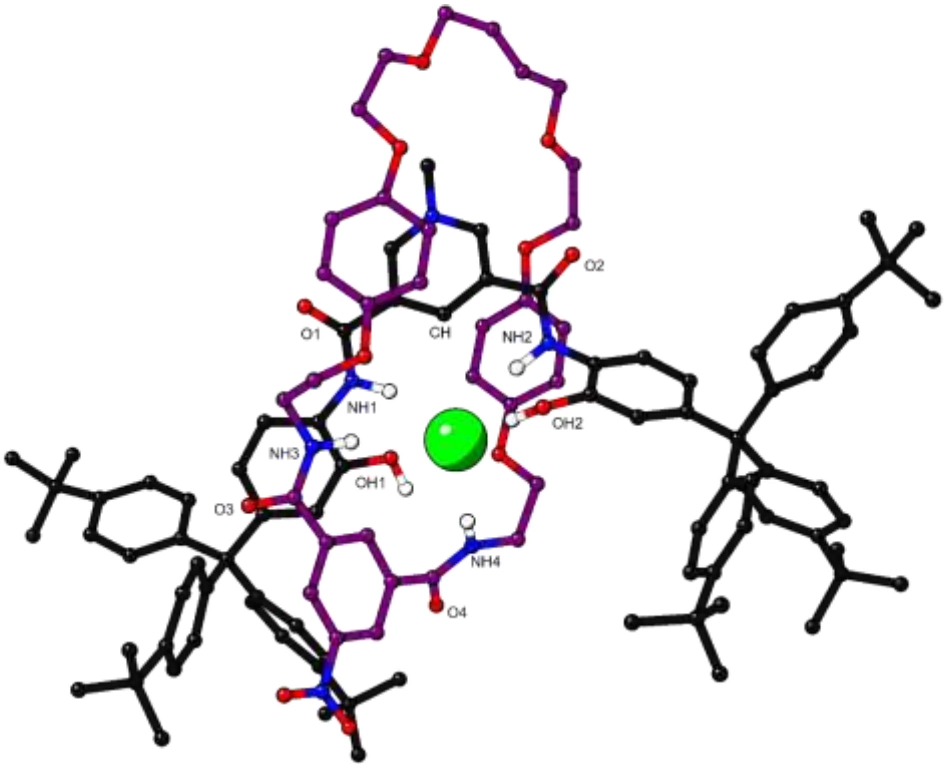

Figure S80. *Starting geometry of **5·Cl**. Atom labels are coordinated with graph legends in Figures S125-S133.*

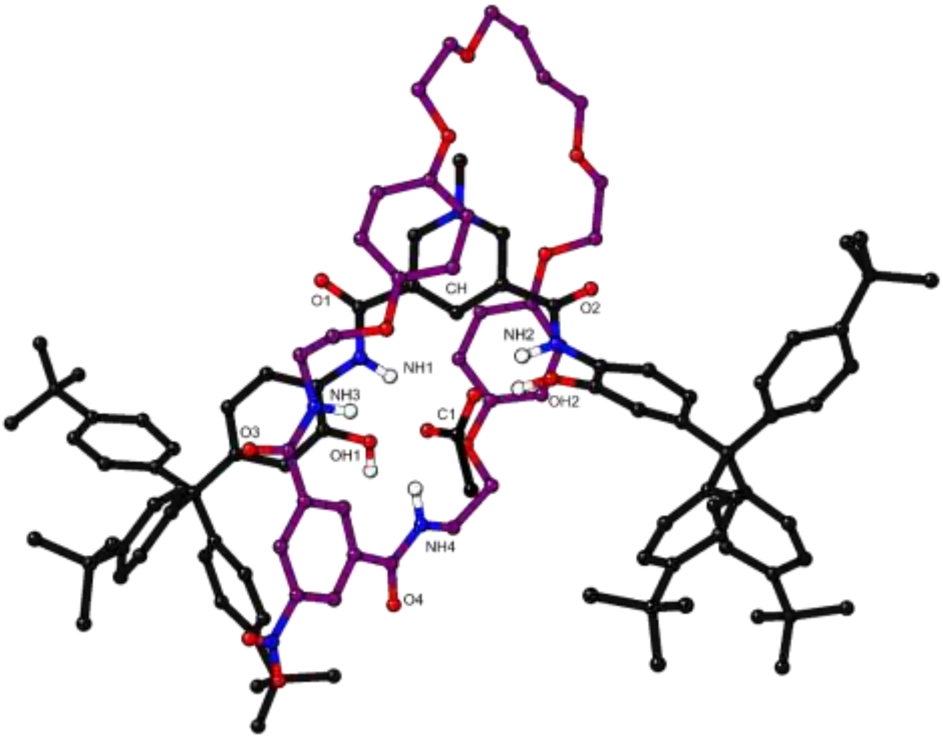

Figure S81. *Starting geometry of **5·OAc**. Atom labels are coordinated with graph legends in Figures S134-S142.*

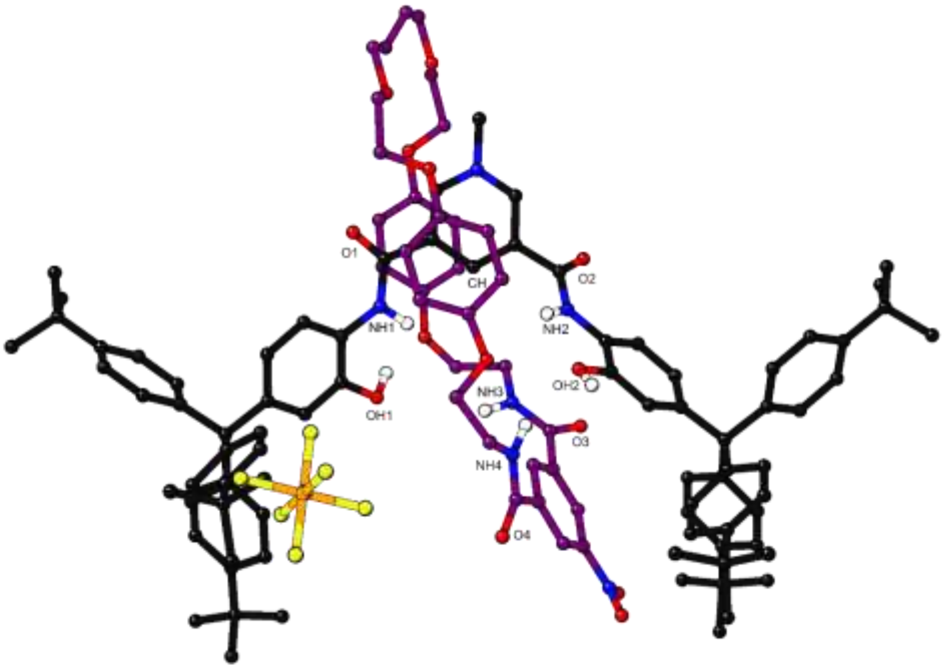

Figure S82. *Starting geometry of* ***5·PF₆***. *Atom labels are coordinated with graph legends in Figures S143-S148.*

*Time trace graphs of **1·Cl***

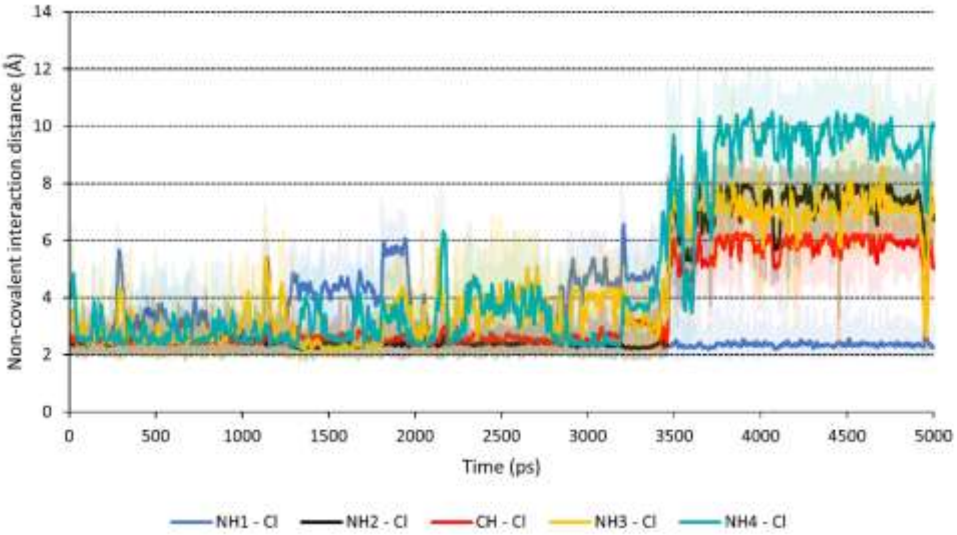

Figure S83. 1·Cl *time trace 1 of non-covalent interactions between hydrogen bond donors and anion.*

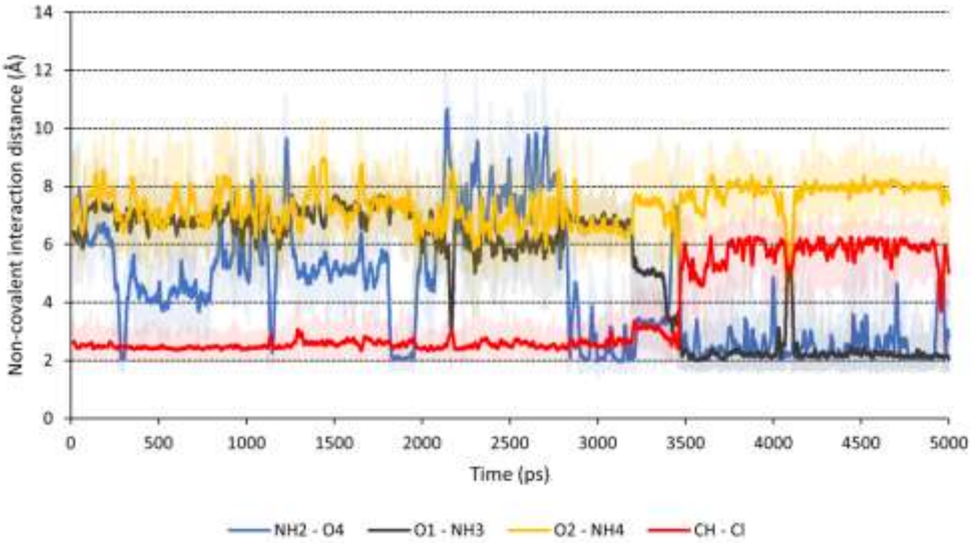

Figure S84. 1·Cl *time trace 1 of non-covalent intercomponent interactions compared to the interior pyridinium proton distance to the anion (CH – Cl).*

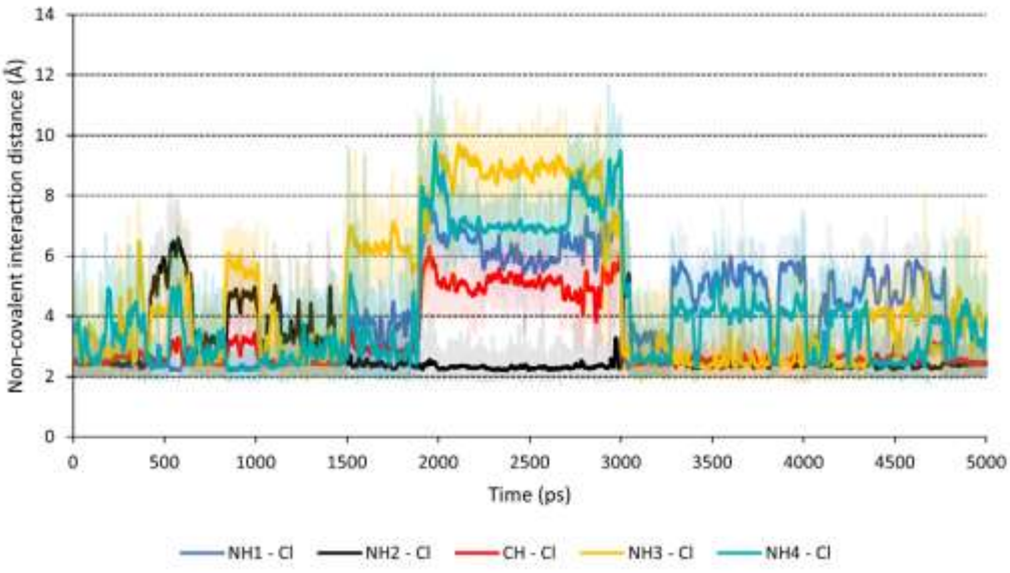

Figure S85. 1·Cl *time trace 2 of non-covalent interactions between hydrogen bond donors and anion.*

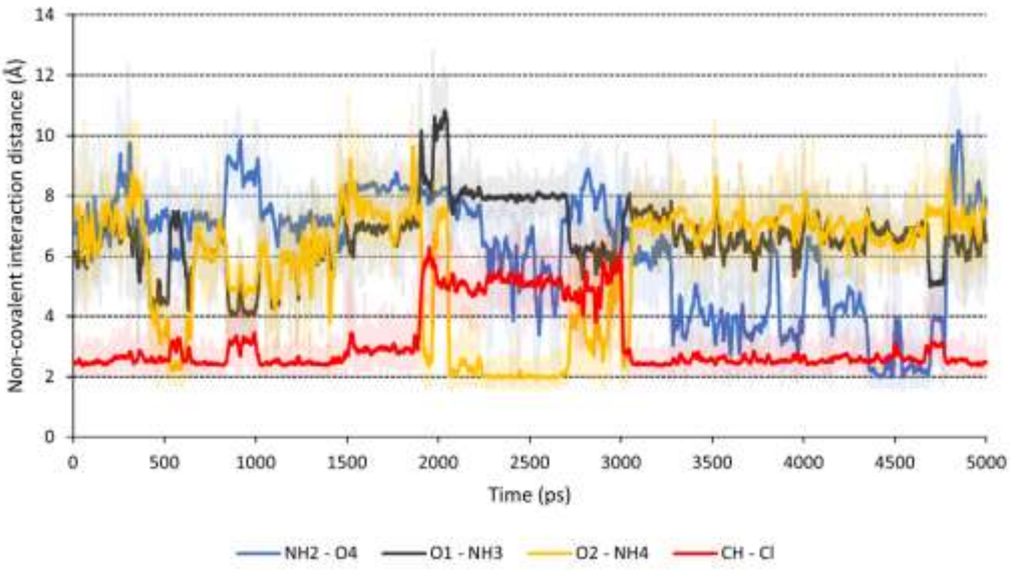

Figure S86. 1·Cl *time trace 2 of non-covalent intercomponent interactions compared to the interior pyridinium proton distance to the anion (CH – Cl).*

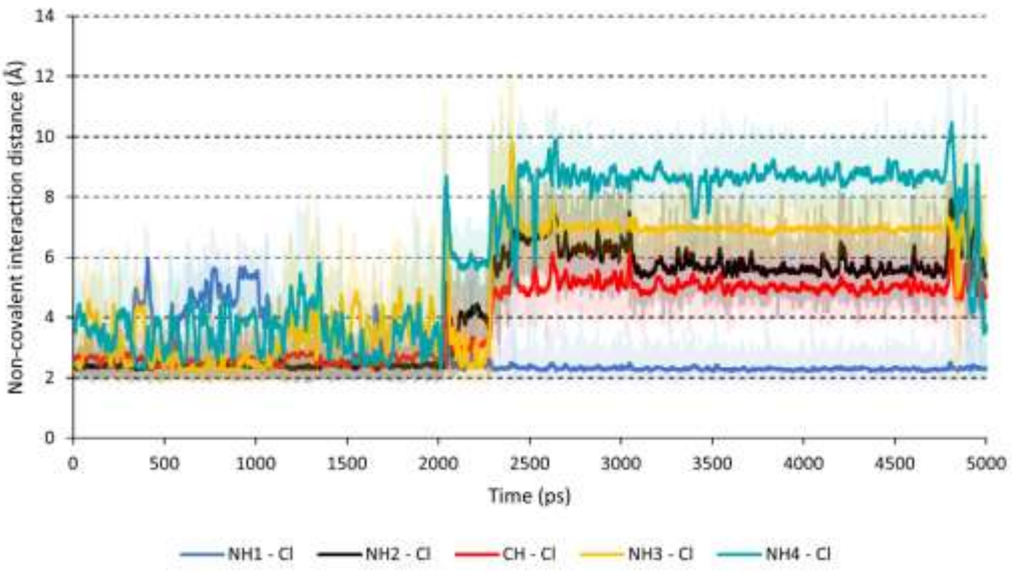

***Figure S87. 1·Cl** time trace 3 of non-covalent interactions between hydrogen bond donors and anion.*

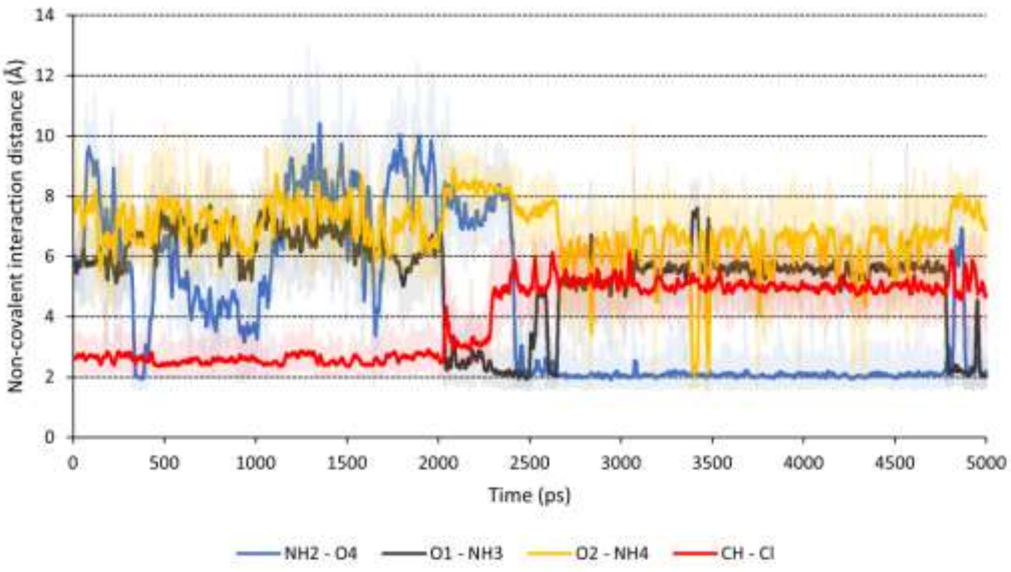

***Figure S88. 1·Cl** time trace 3 of non-covalent intercomponent interactions compared to the interior pyridinium proton distance to the anion (CH – Cl).*

Time trace graphs of ***1·OAc***

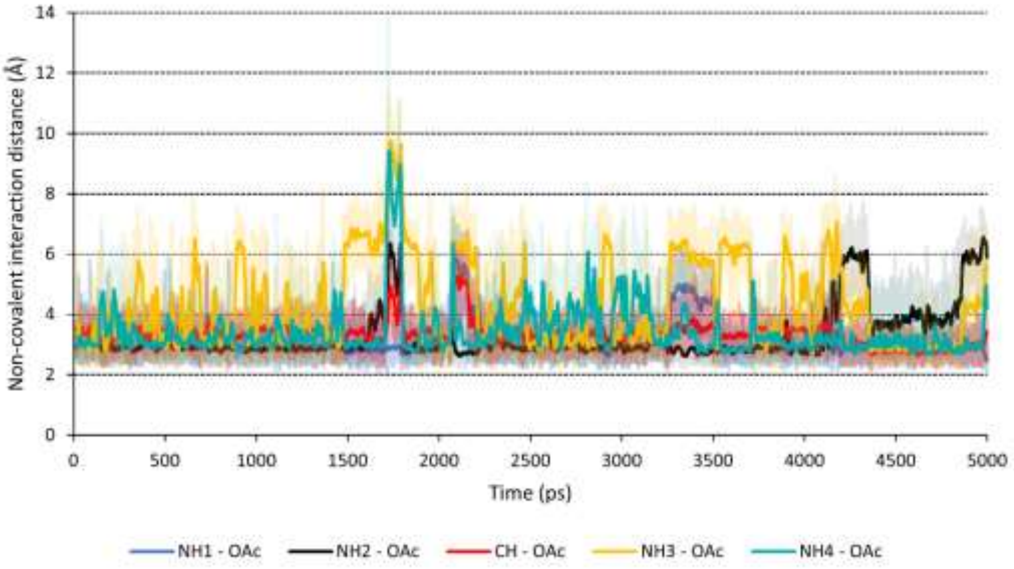

Figure S89. 1·OAc *time trace 1 of non-covalent interactions between hydrogen bond donors and anion.*

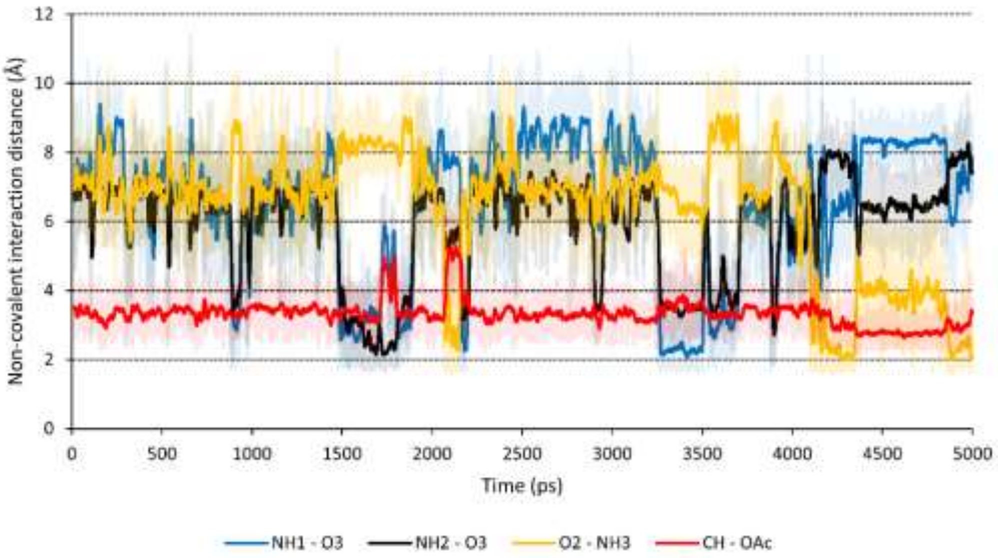

Figure S90. 1·OAc *time trace 1 of non-covalent intercomponent interactions compared to the interior pyridinium proton distance to the anion (CH – OAc).*

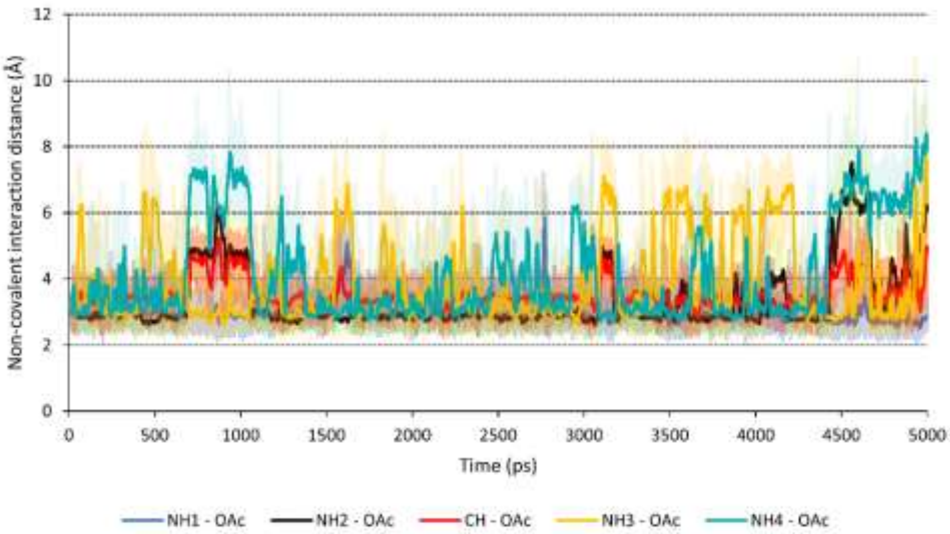

Figure S91. 1·OAc *time trace 2 of non-covalent interactions between hydrogen bond donors and anion.*

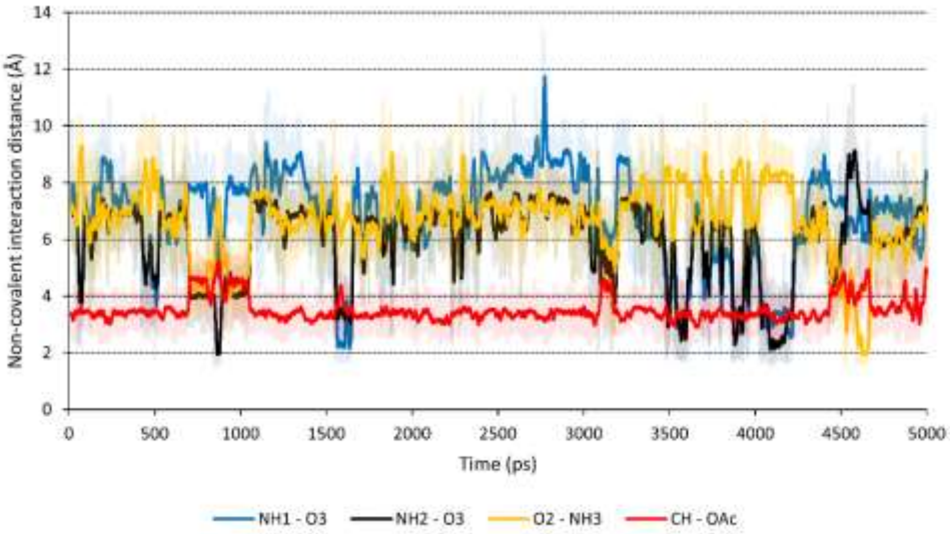

Figure S92. 1·OAc *time trace 2 of non-covalent intercomponent interactions compared to the interior pyridinium proton distance to the anion (CH – OAc).*

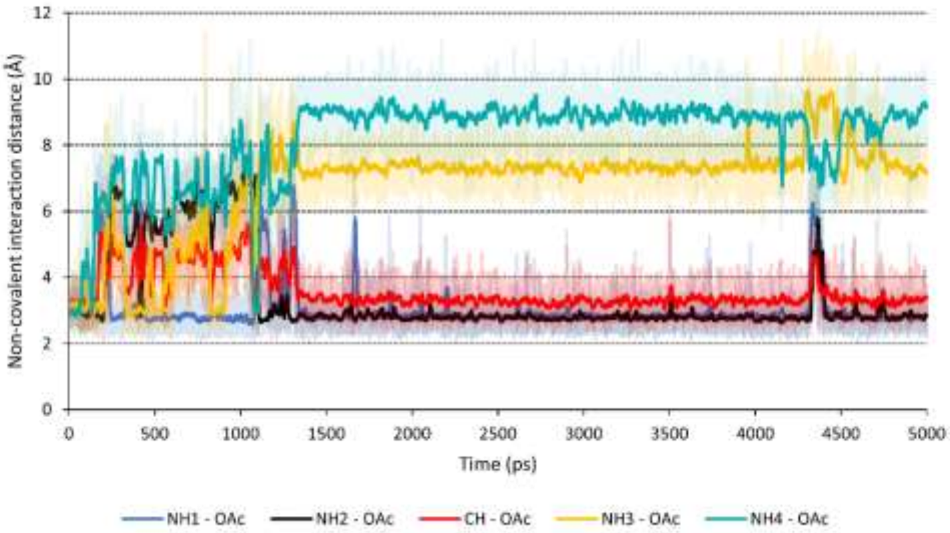

Figure S93. 1·OAc *time trace 3 of non-covalent interactions between hydrogen bond donors and anion.*

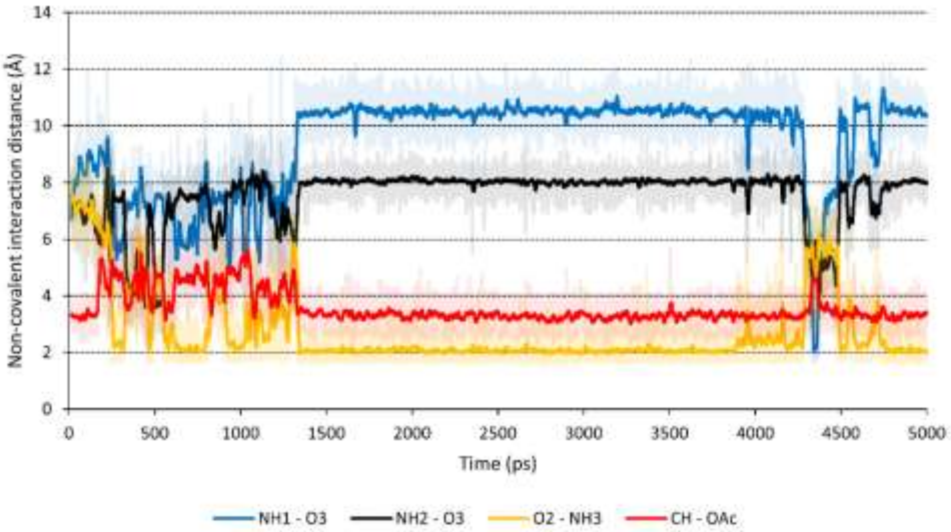

Figure S94. 1·OAc *time trace 3 of non-covalent intercomponent interactions compared to the interior pyridinium proton distance to the anion (CH – OAc).*

Time trace graphs of ***1·PF₆***

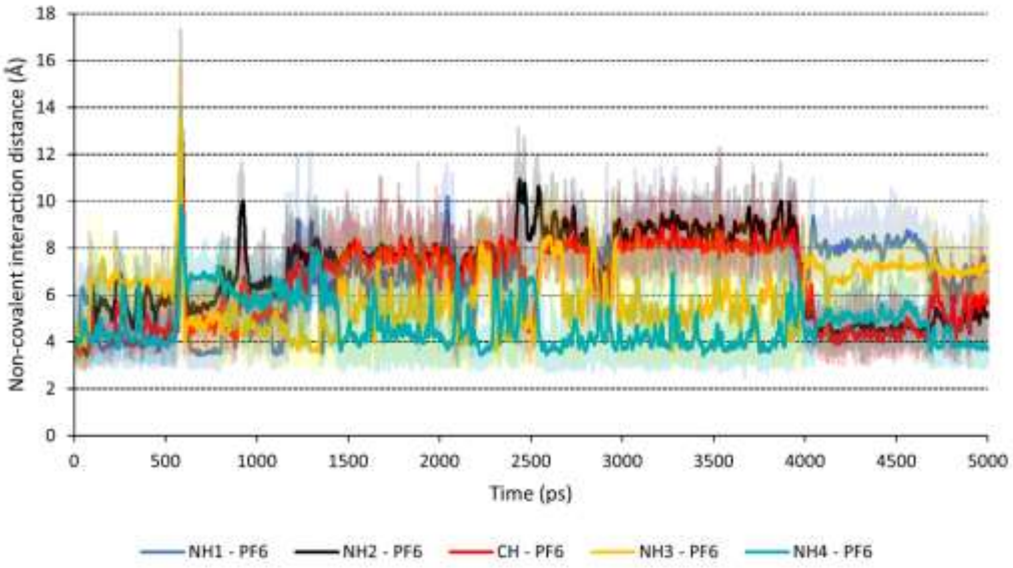

Figure S95. 1·PF$_6$ *time trace 1 of non-covalent interactions between hydrogen bond donors and anion.*

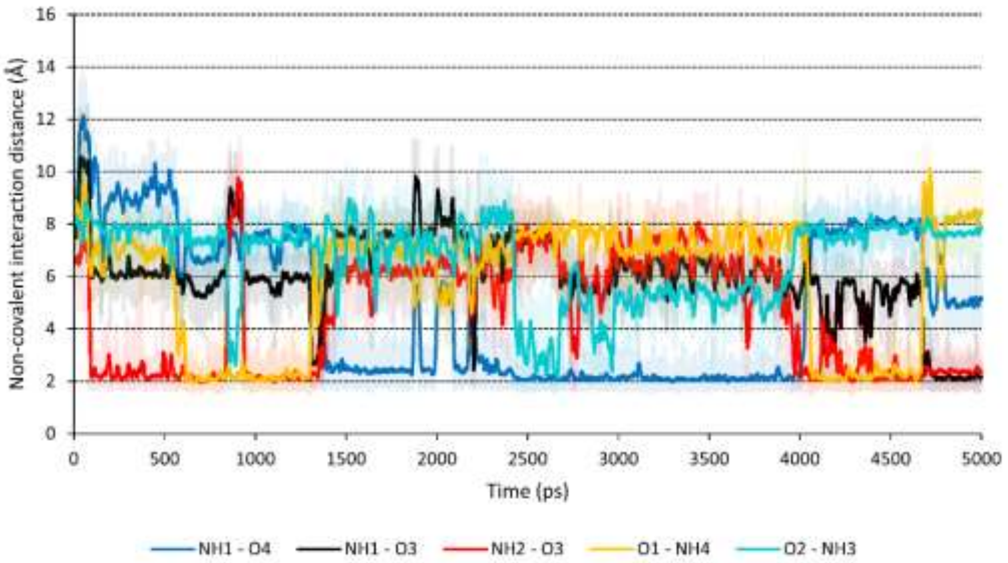

Figure S96. 1·PF$_6$ *time trace 1 of non-covalent intercomponent interactions.*

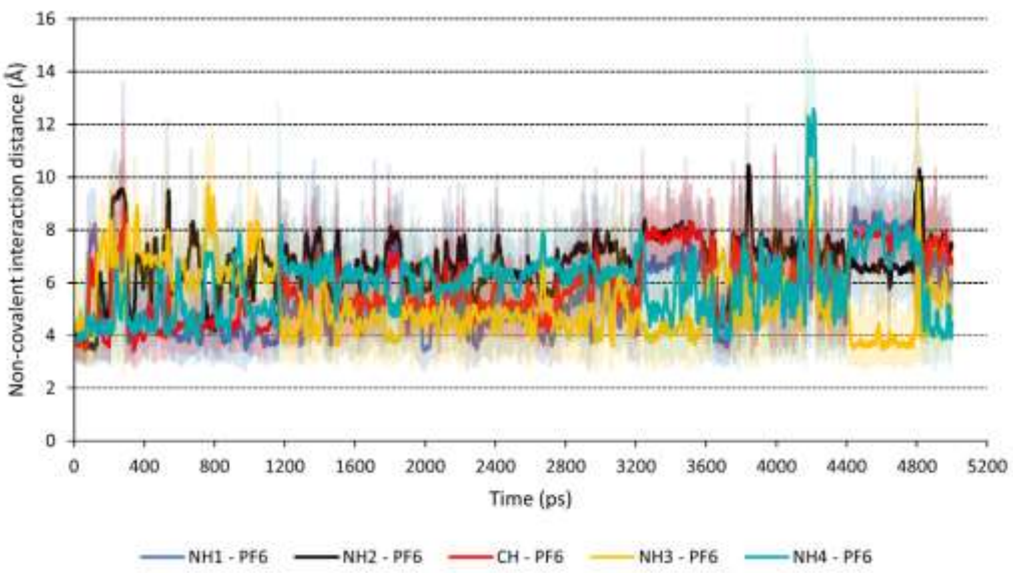

Figure S97. 1·PF$_6$ *time trace 2 of non-covalent interactions between hydrogen bond donors and anion.*

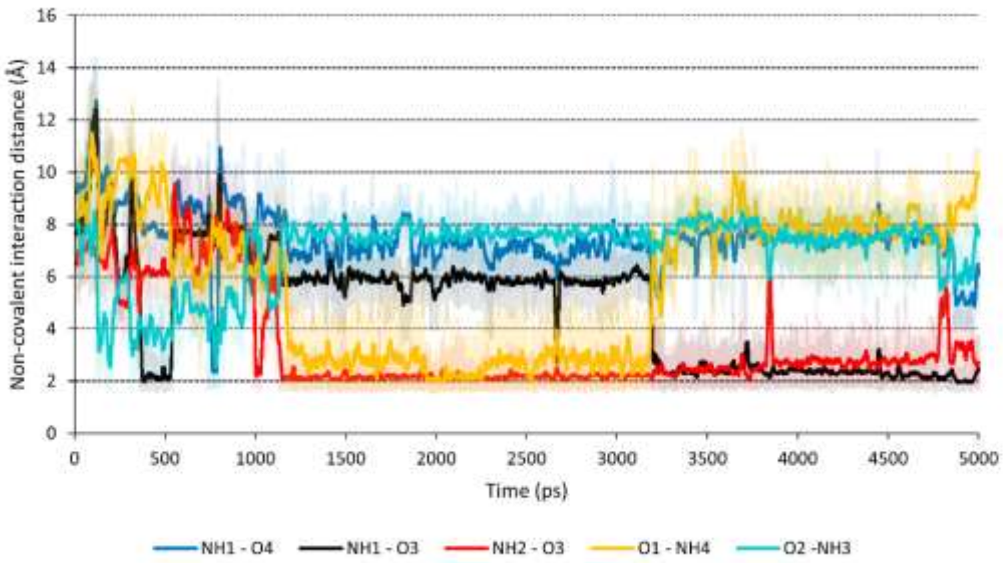

Figure S98. 1·PF$_6$ *time trace 2 of non-covalent intercomponent interactions.*

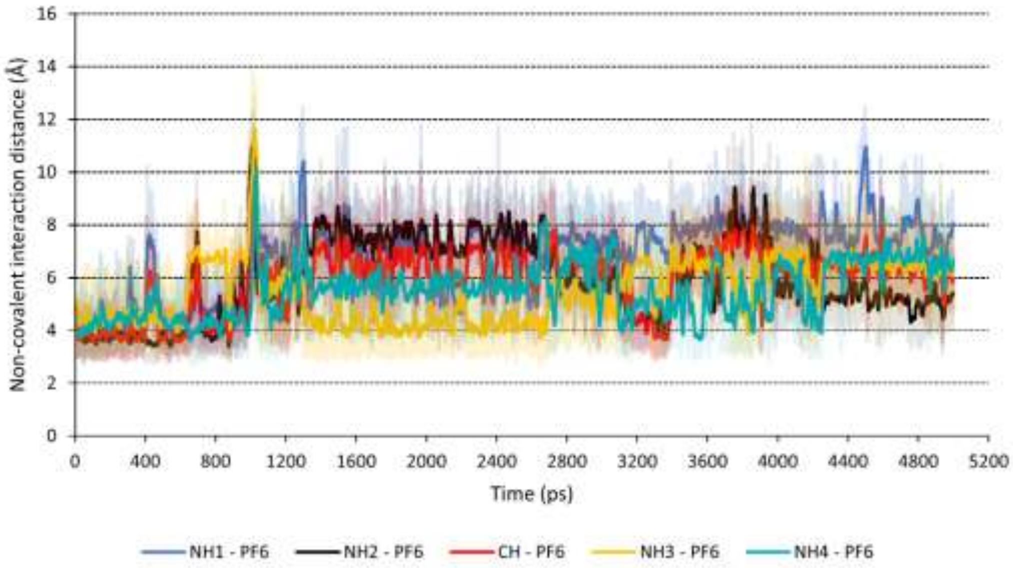

Figure S99. 1·PF$_6$ *time trace 3 of non-covalent interactions between hydrogen bond donors and anion.*

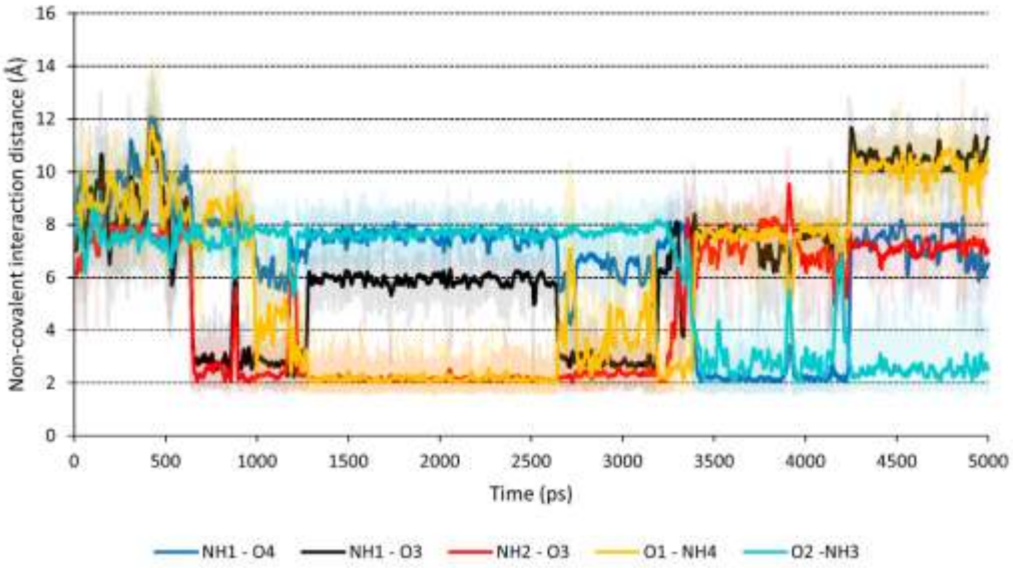

Figure S100. 1·PF$_6$ *time trace 3 of non-covalent intercomponent interactions.*

*Time trace graphs of **4·Cl***

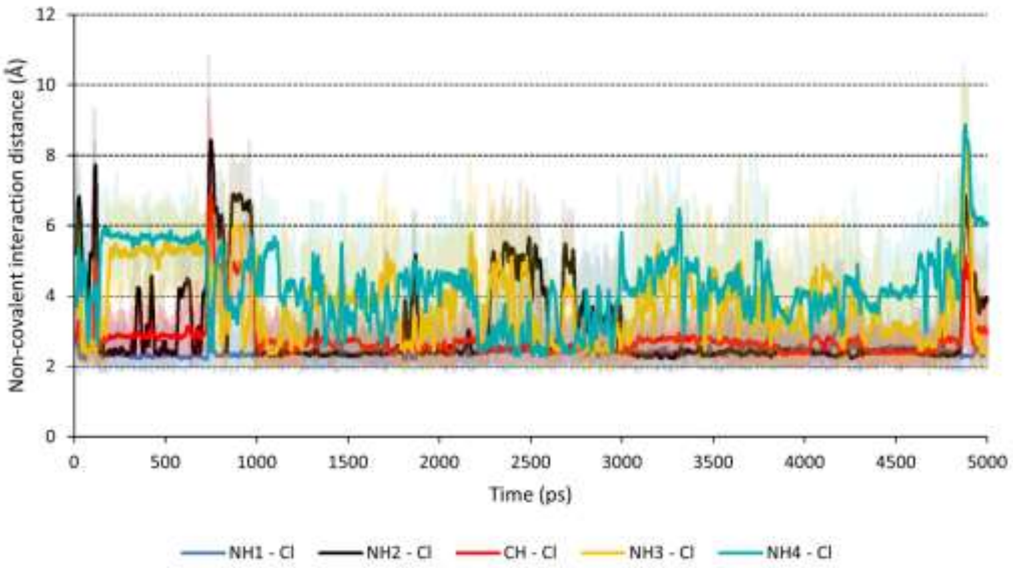

***Figure S101. 4·Cl** time trace 1 of non-covalent interactions between hydrogen bond donors and anion.*

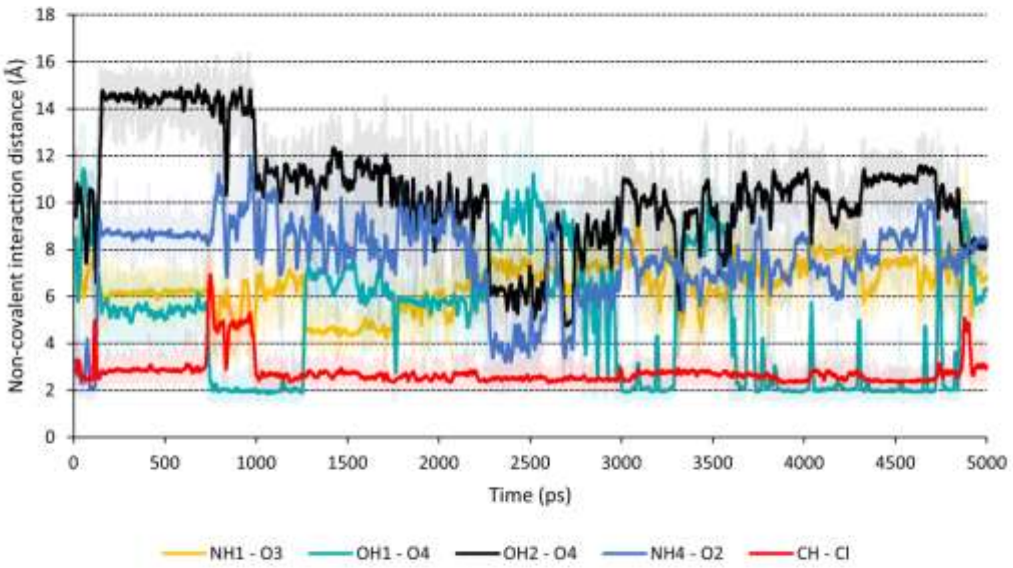

***Figure S102. 4·Cl** time trace 1 of non-covalent intercomponent interactions compared to the interior pyridinium proton distance to the anion (CH – Cl).*

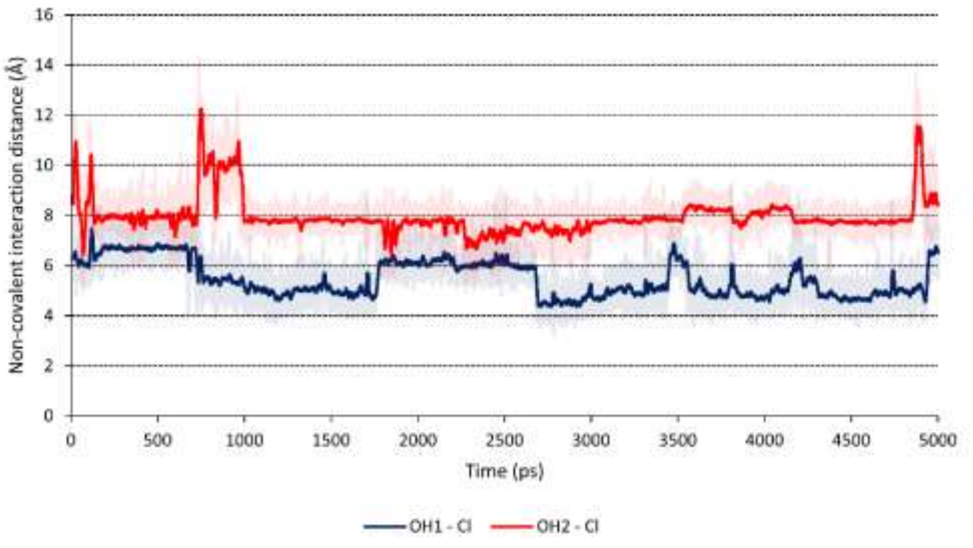

Figure S103. 4·Cl *time trace 1 of non-covalent interactions between hydroxy bond donors and anion.*

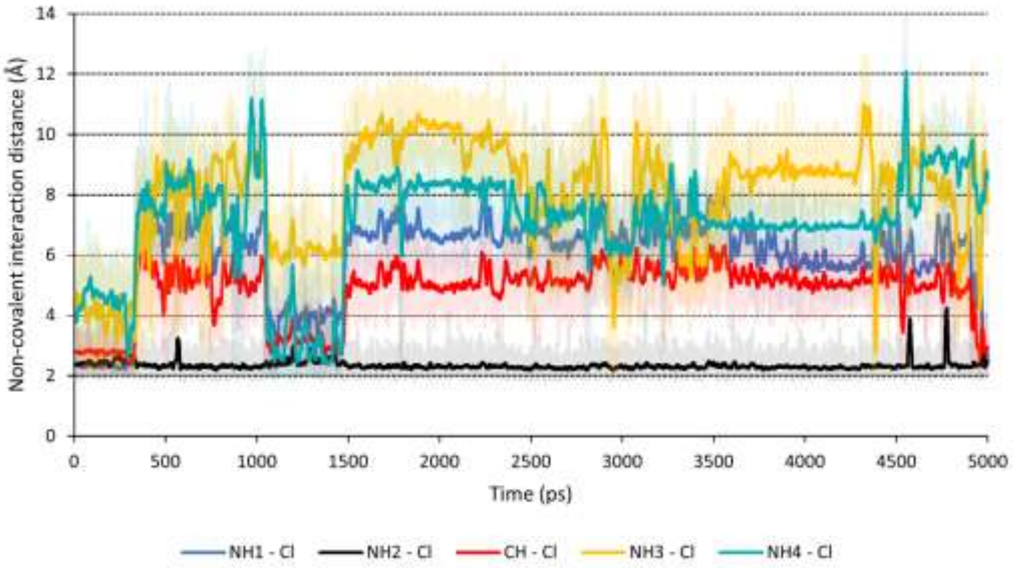

Figure S104. 4·Cl *time trace 2 of non-covalent interactions between hydrogen bond donors and anion.*

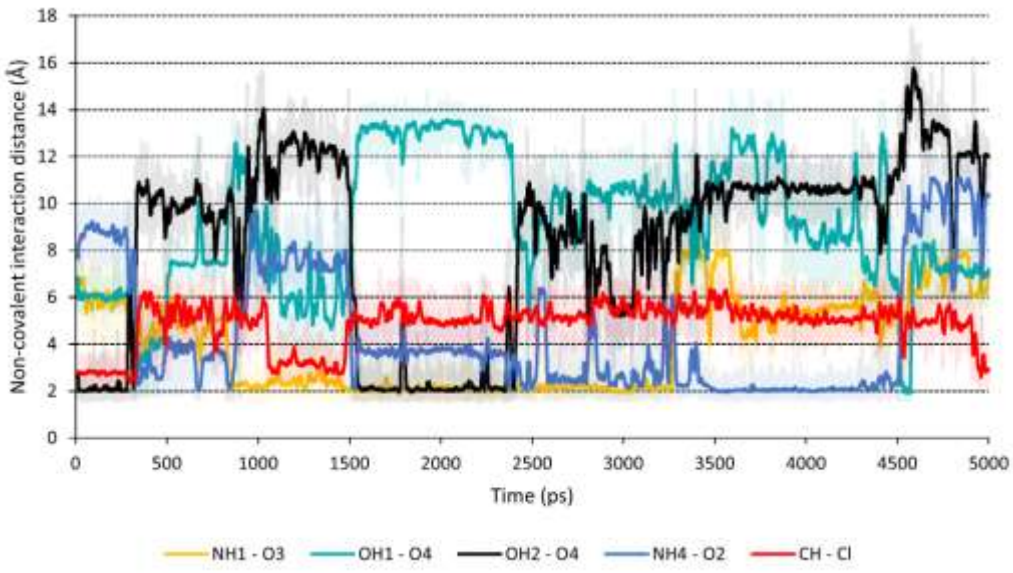

Figure S105. 4·Cl *time trace 2 of non-covalent intercomponent interactions compared to the interior pyridinium proton distance to the anion (CH – Cl).*

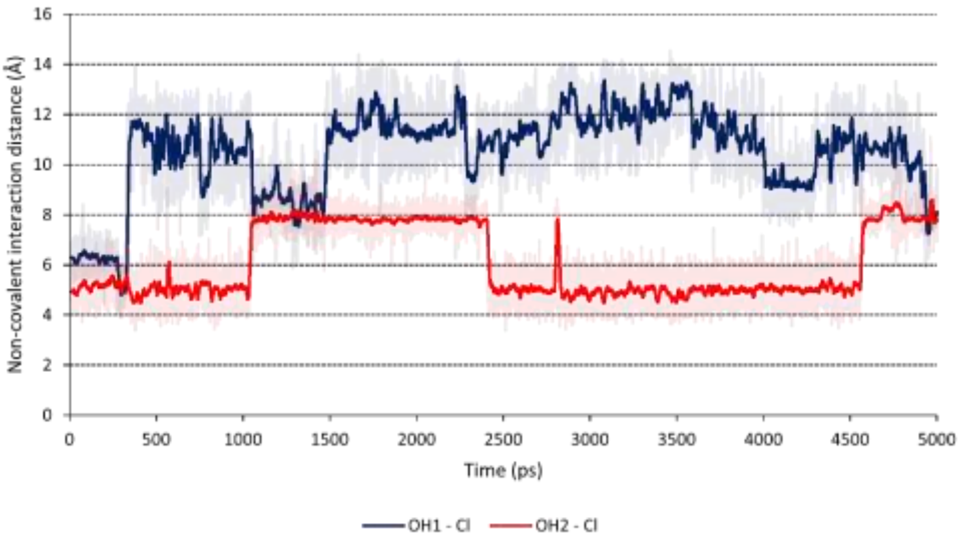

Figure S106. 4·Cl *time trace 2 of non-covalent interactions between hydroxy bond donors and anion.*

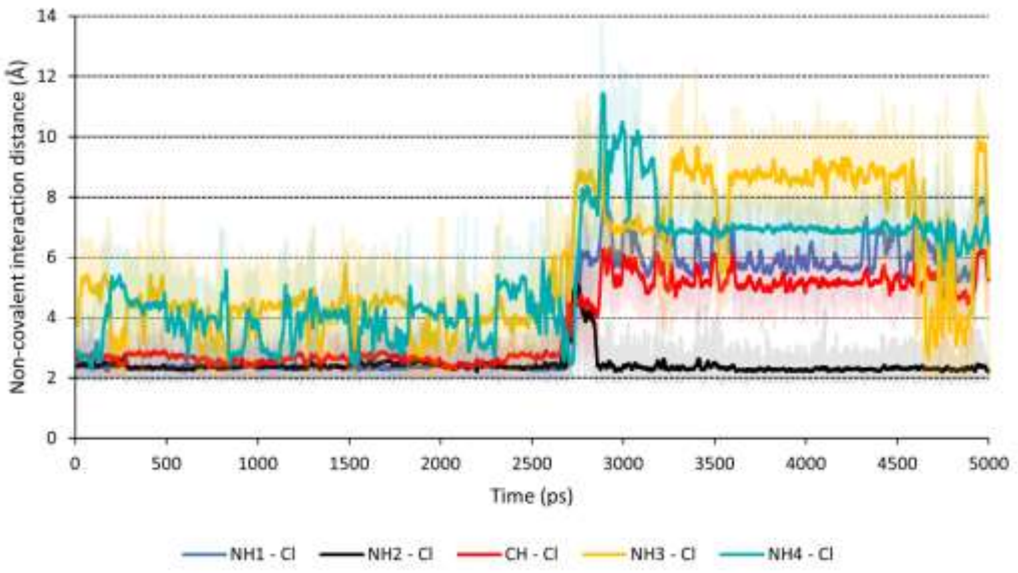

***Figure S107. 4·Cl** time trace 3 of non-covalent interactions between hydrogen bond donors and anion.*

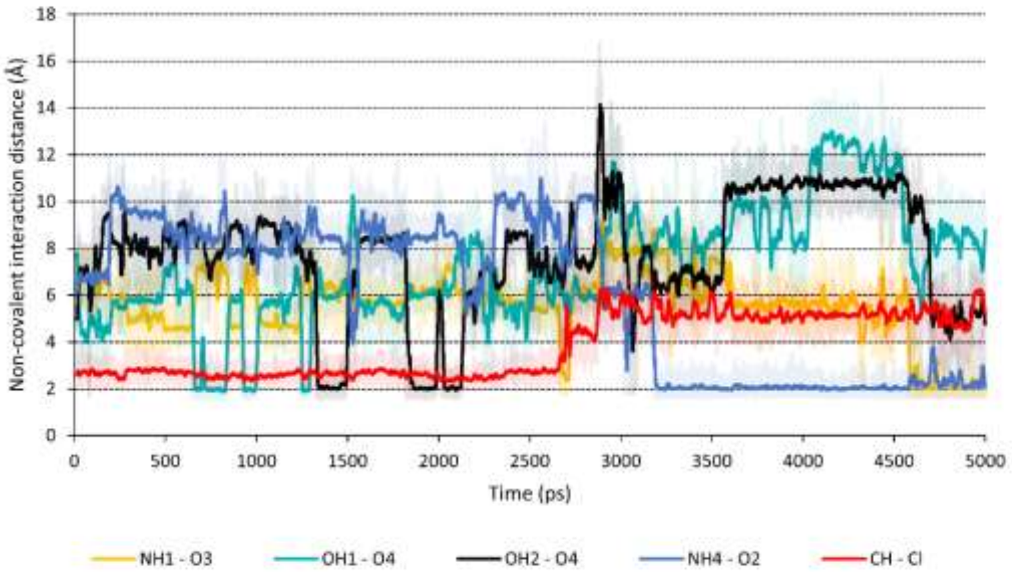

***Figure S108. 4·Cl** time trace 3 of non-covalent intercomponent interactions compared to the interior pyridinium proton distance to the anion (CH – Cl).*

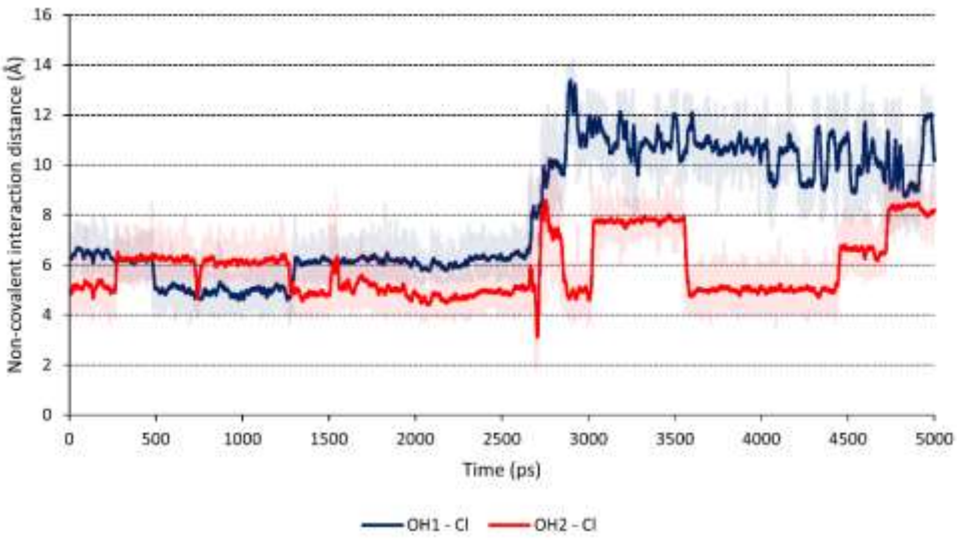

***Figure S109. 4·Cl** time trace 3 of non-covalent interactions between hydroxy bond donors and anion.*

*Time trace graphs of **4·OAc***

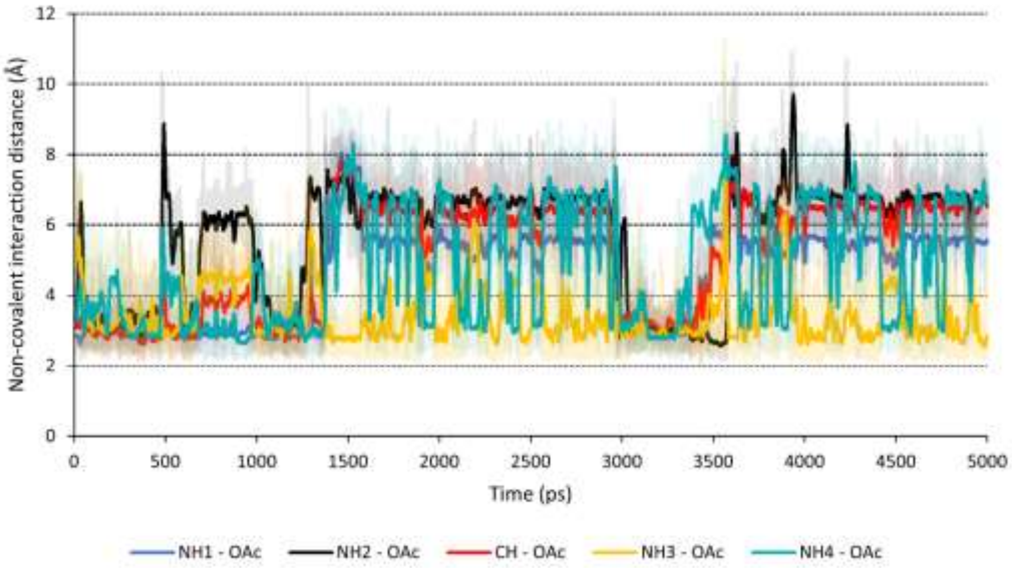

***Figure S110. 4·OAc** time trace 1 of non-covalent interactions between hydrogen bond donors and anion.*

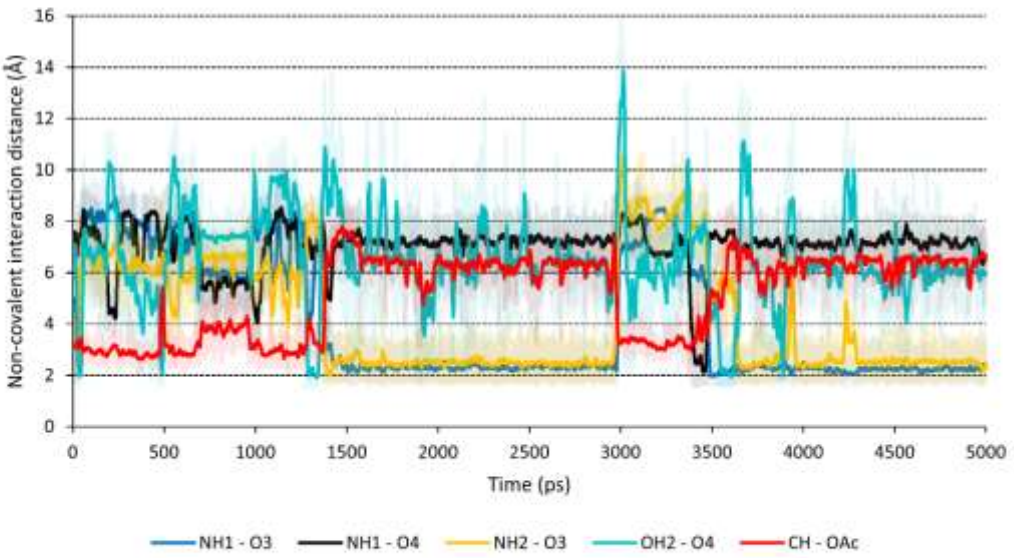

Figure S111. 4·OAc *time trace 1 of non-covalent intercomponent interactions compared to the interior pyridinium proton distance to the anion (CH – OAc).*

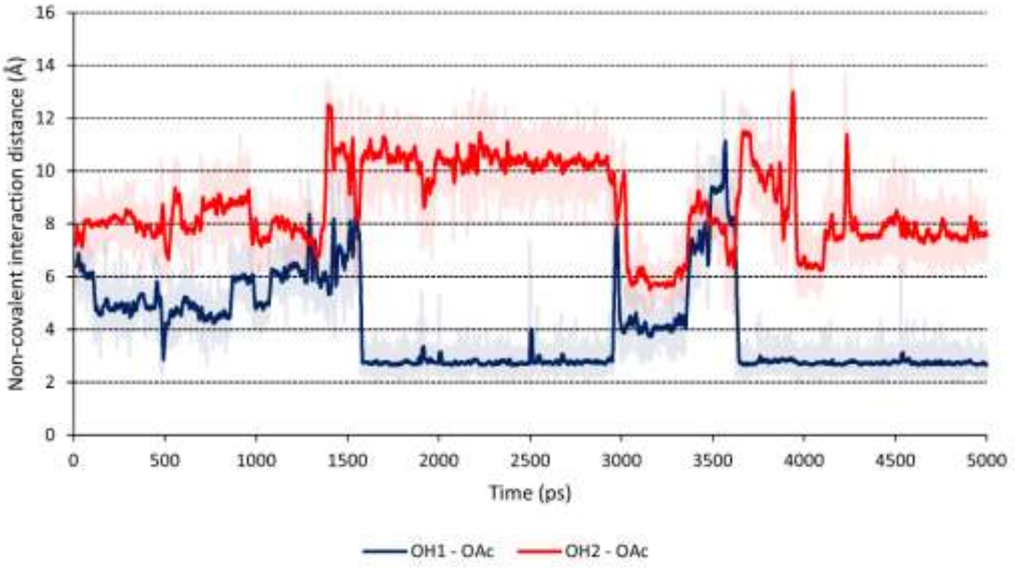

Figure S112. 4·OAc *time trace 1 of non-covalent interactions between hydroxy bond donors and anion.*

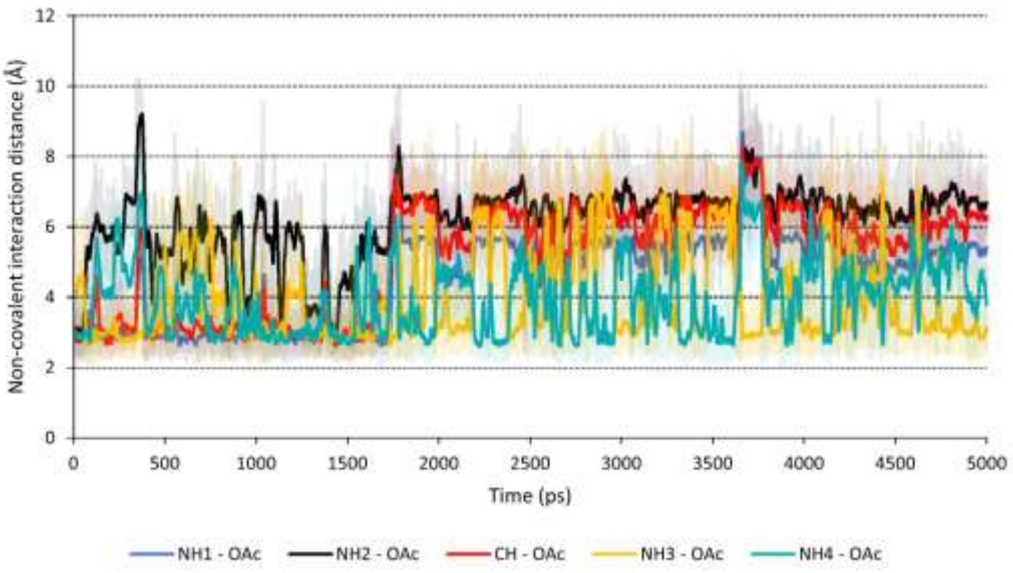

***Figure S113. 4·OAc** time trace 2 of non-covalent interactions between hydrogen bond donors and anion.*

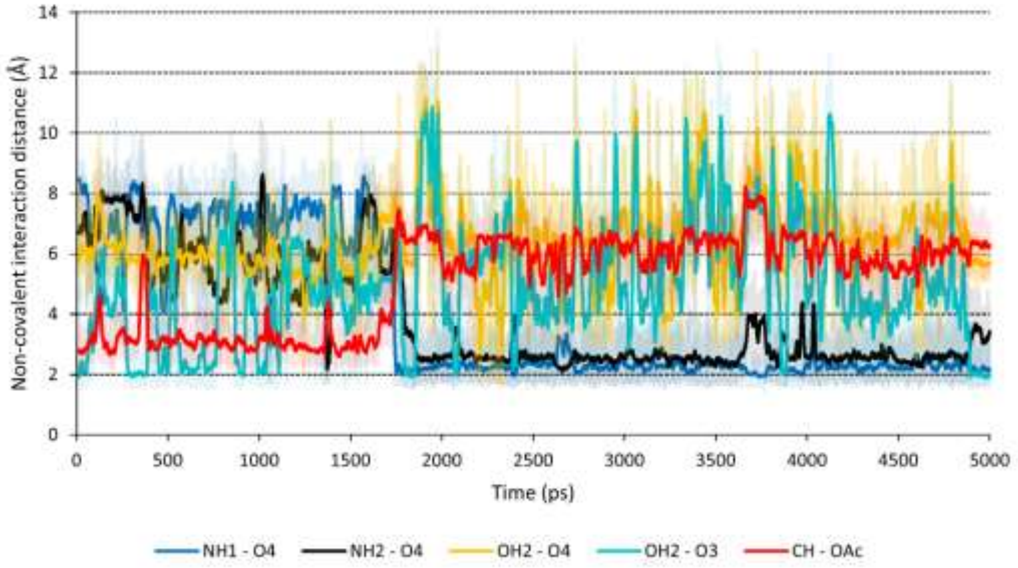

***Figure S114. 4·OAc** time trace 2 of non-covalent intercomponent interactions compared to the interior pyridinium proton distance to the anion (CH – OAc).*

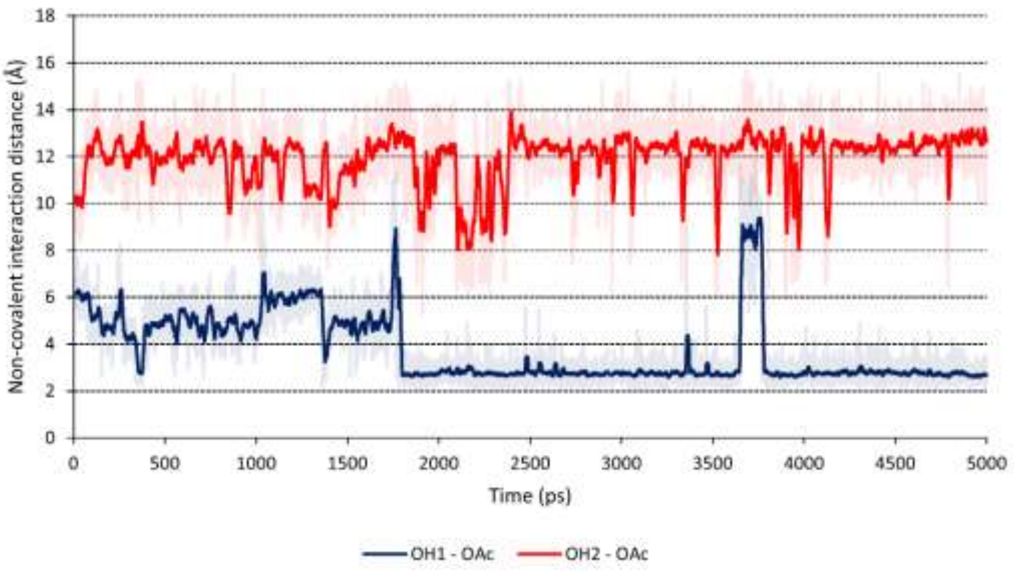

Figure S115. 4·OAc *time trace 2 of non-covalent interactions between hydroxy bond donors and anion.*

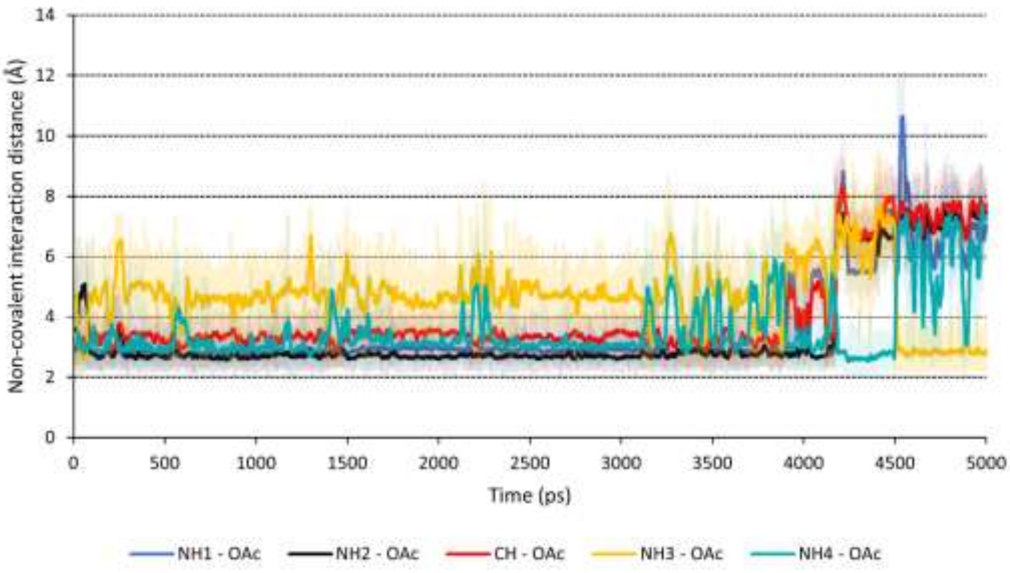

Figure S116. 4·OAc *time trace 3 of non-covalent interactions between hydrogen bond donors and anion.*

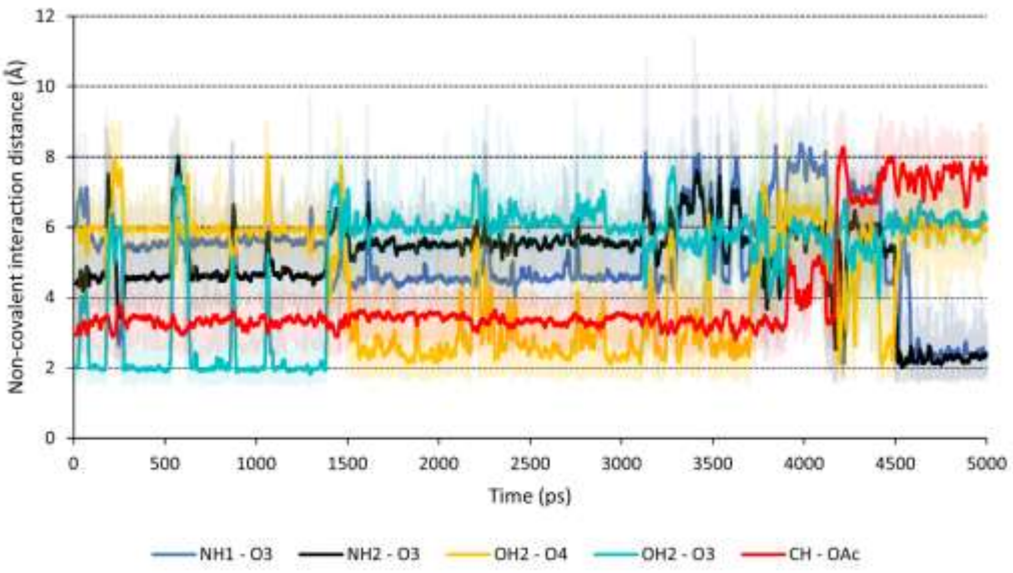

***Figure S117. 4·OAc** time trace 3 of non-covalent intercomponent interactions compared to the interior pyridinium proton distance to the anion (CH – OAc).*

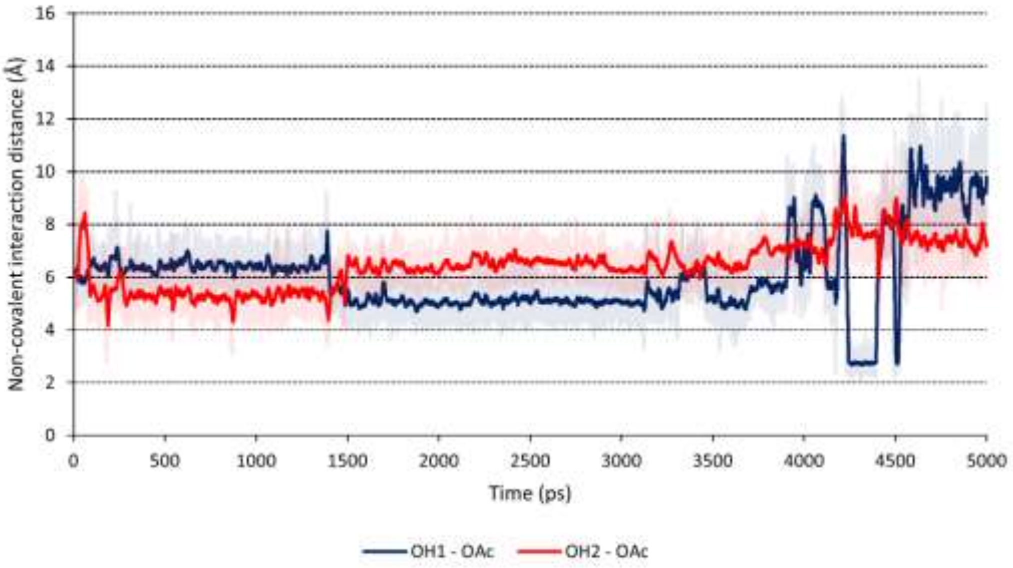

***Figure S118. 4·OAc** time trace 3 of non-covalent interactions between hydroxy bond donors and anion.*

*Time trace graphs of **4·PF$_6$***

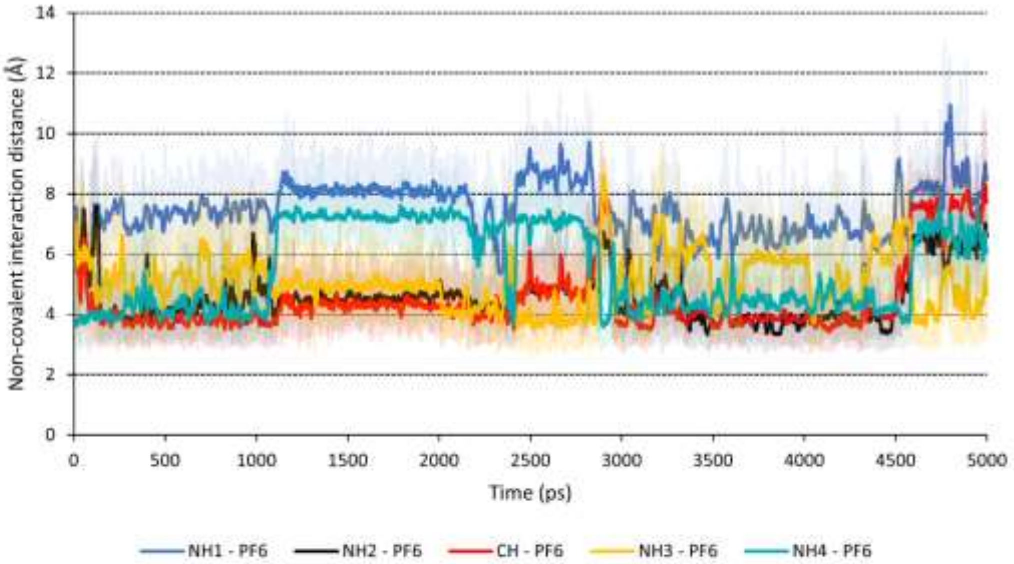

Figure S119. 4·PF$_6$ *time trace 1 of non-covalent interactions between hydrogen bond donors and anion.*

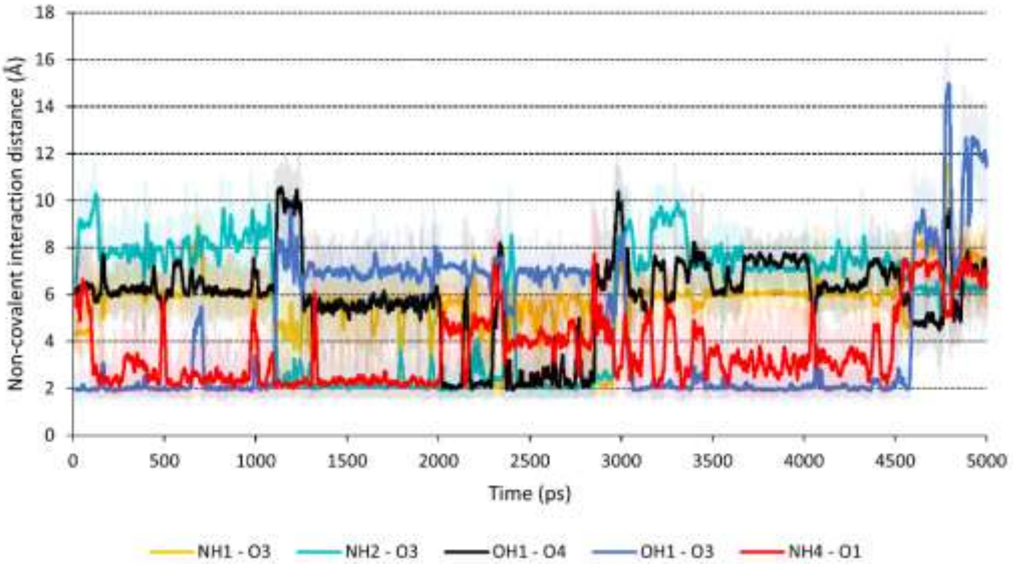

Figure S120. 4·PF$_6$ *time trace 1 of non-covalent intercomponent interactions.*

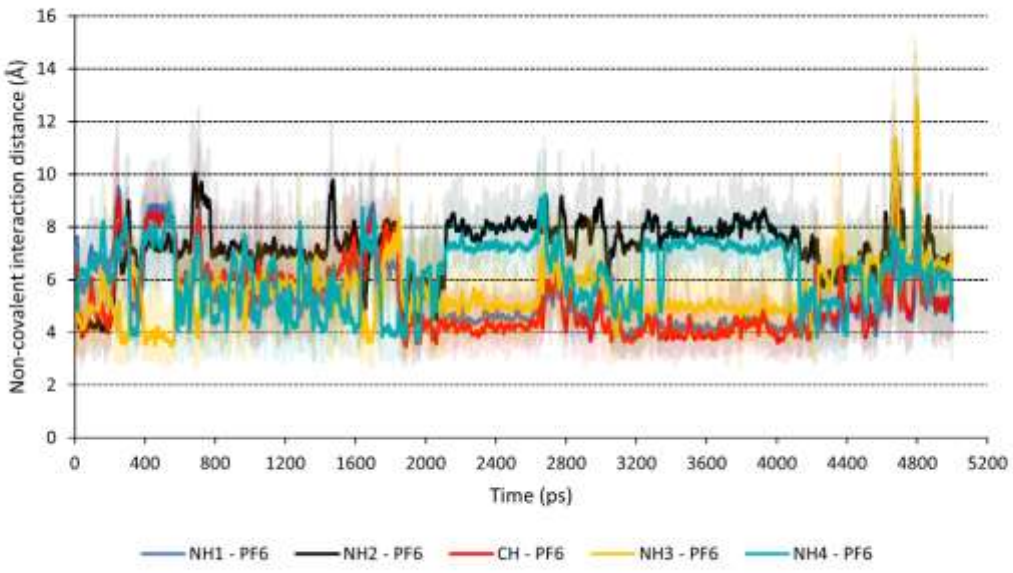

Figure S121. 4·PF₆ *time trace 2 of non-covalent interactions between hydrogen bond donors and anion.*

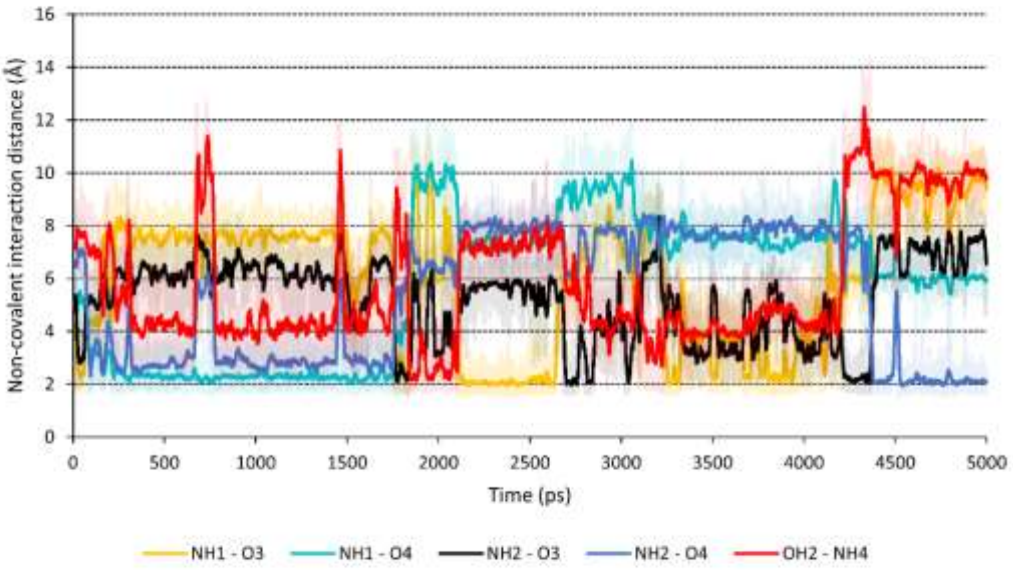

Figure S122. 4·PF₆ *time trace 2 of non-covalent intercomponent interactions.*

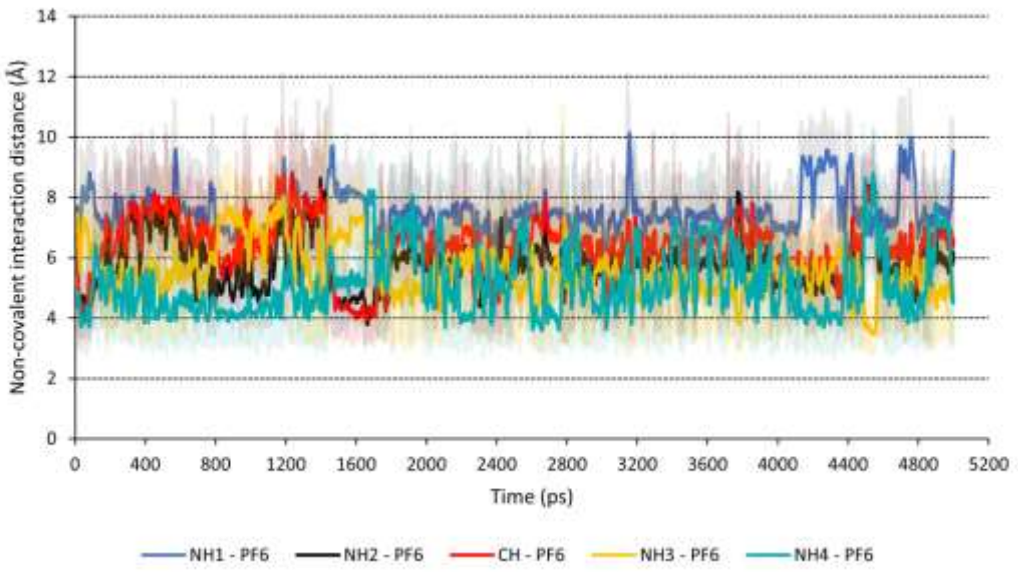

***Figure S123. 4·PF$_6$** time trace 3 of non-covalent interactions between hydrogen bond donors and anion.*

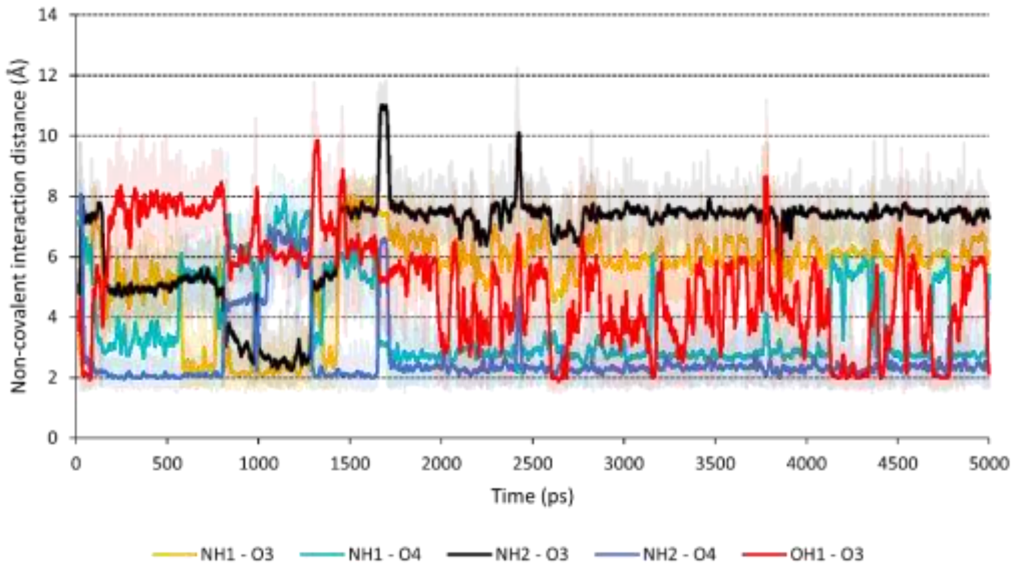

***Figure S124. 4·PF$_6$** time trace 3 of non-covalent intercomponent interactions.*

*Time trace graphs of **5·Cl***

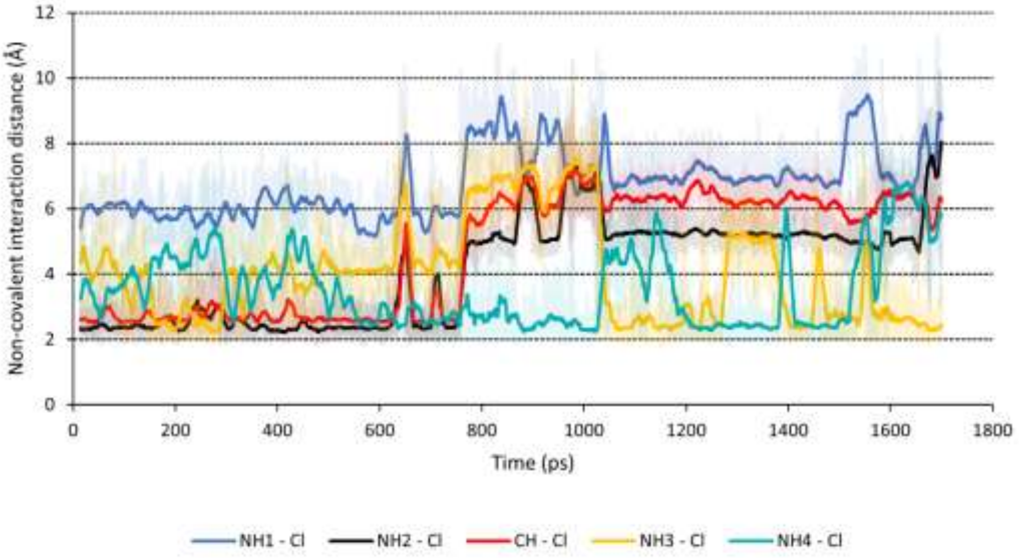

***Figure S125. 5·Cl** time trace 1 of non-covalent interactions between hydrogen bond donors and anion.*

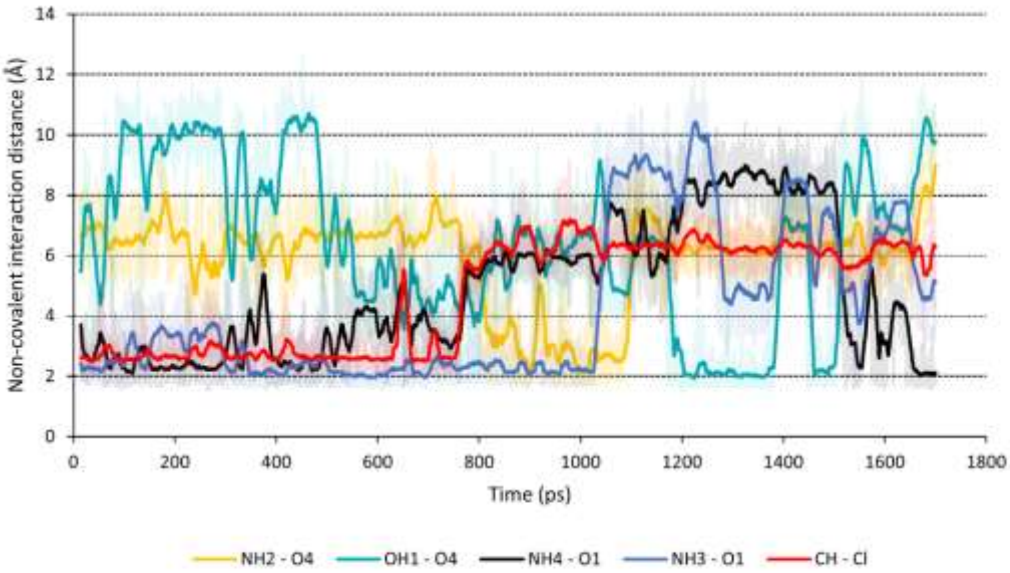

***Figure S126. 5·Cl** time trace 1 of non-covalent intercomponent interactions compared to the interior pyridinium proton distance to the anion (CH – Cl).*

.

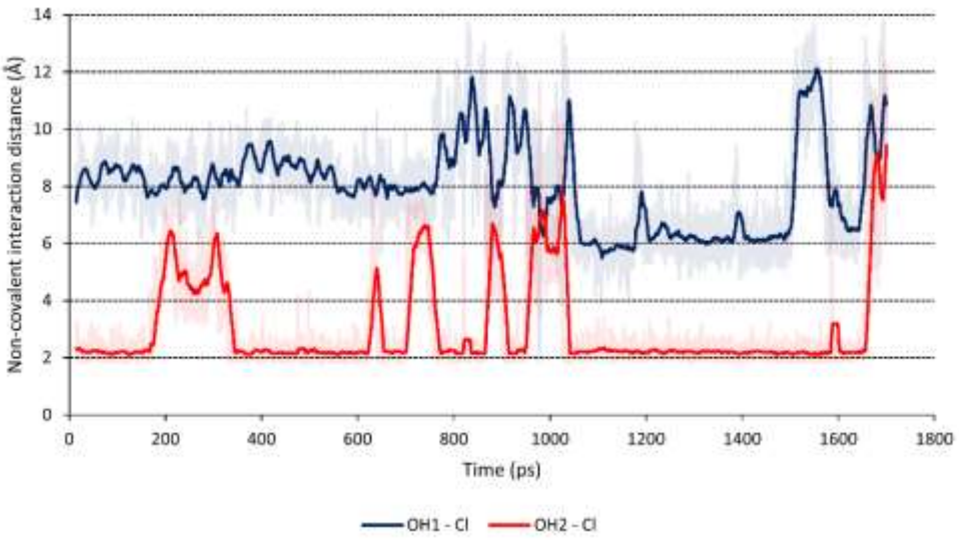

***Figure S127. 5·Cl** time trace 1 of non-covalent interactions between hydroxy bond donors and anion.*

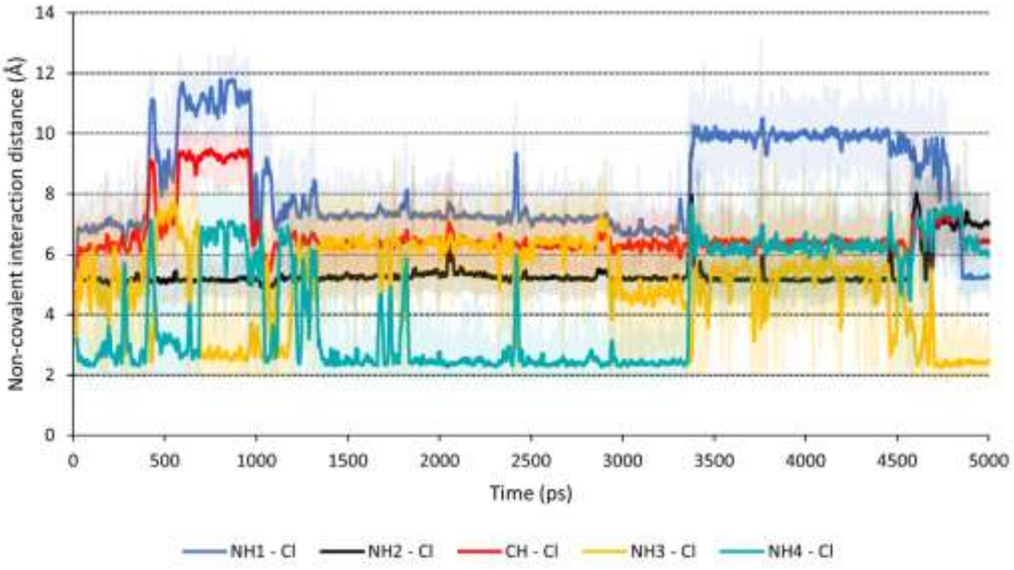

***Figure S128. 5·Cl** time trace 2 of non-covalent interactions between hydrogen bond donors and anion.*

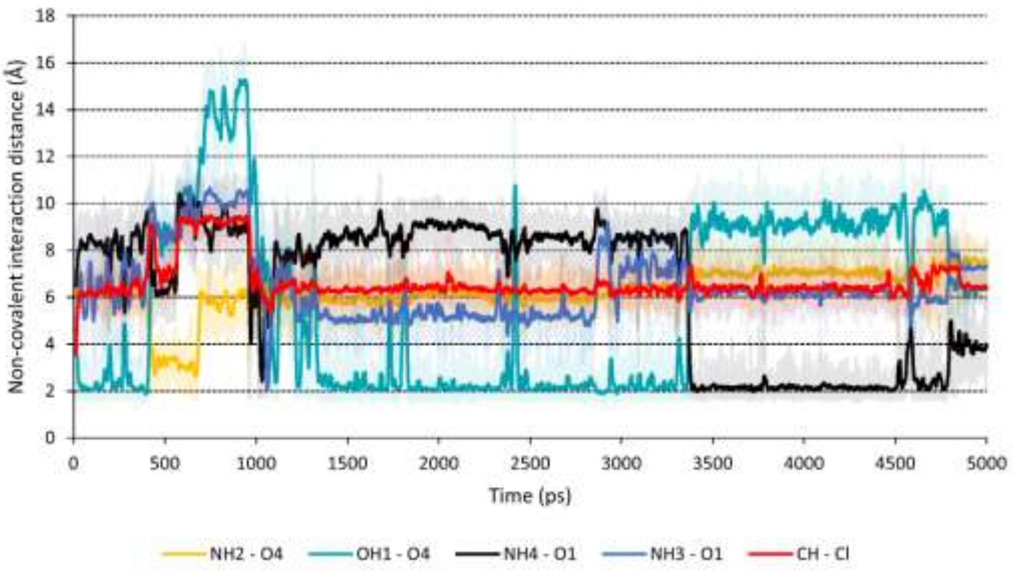

Figure S129. 5·Cl *time trace 2 of non-covalent intercomponent interactions compared to the interior pyridinium proton distance to the anion (CH – Cl).*

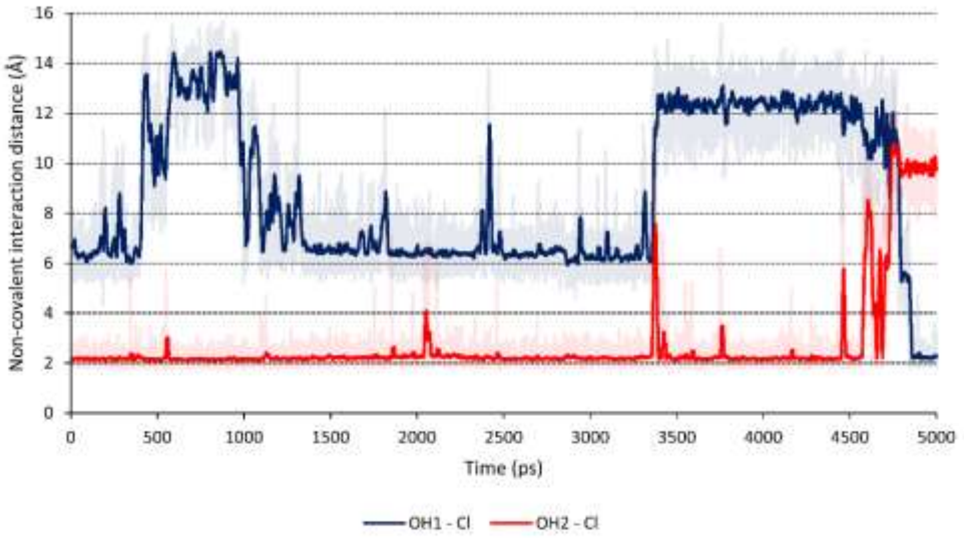

Figure S130. 5·Cl *time trace 2 of non-covalent interactions between hydroxy bond donors and anion.*

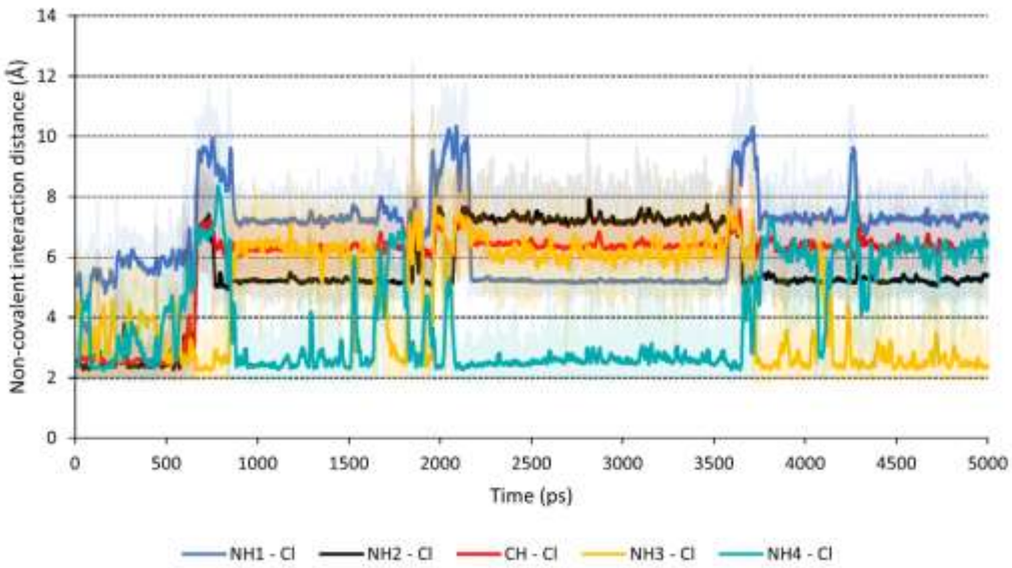

Figure S131. 5·Cl *time trace 3 of non-covalent interactions between hydrogen bond donors and anion.*

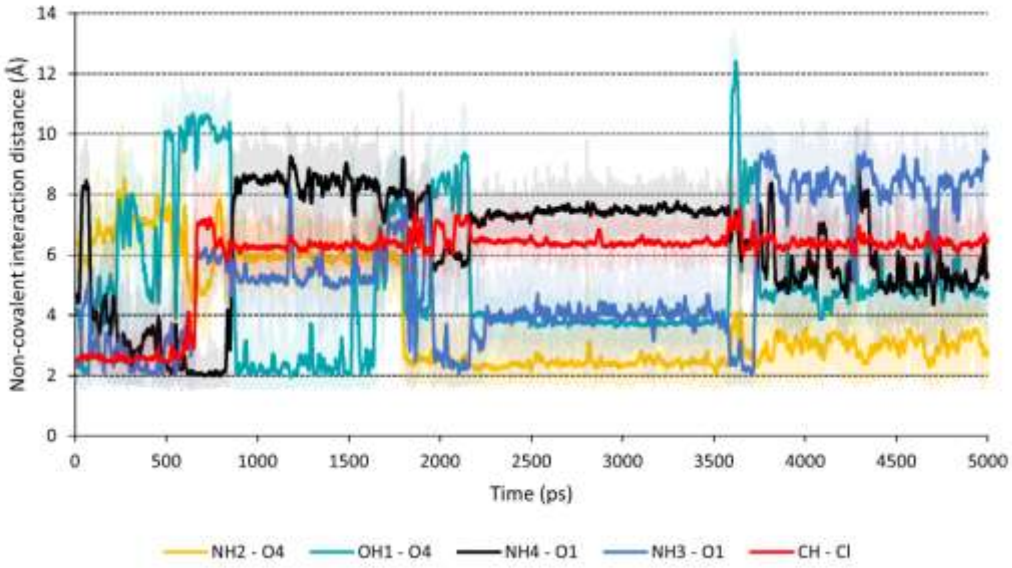

Figure S132. 5·Cl *time trace 3 of non-covalent intercomponent interactions compared to the interior pyridinium proton distance to the anion (CH – Cl).*

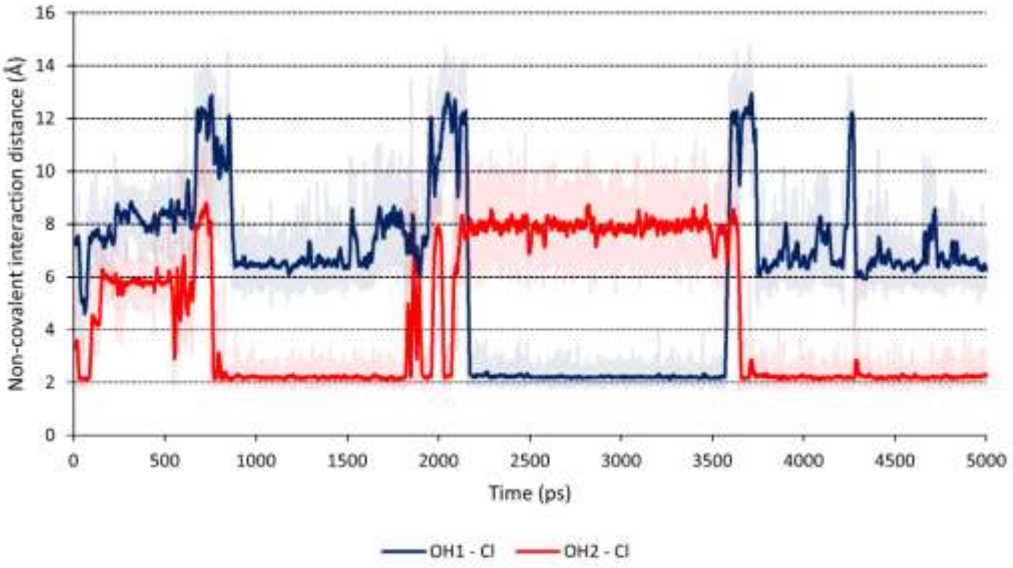

Figure S133. 5·Cl *time trace 3 of non-covalent interactions between hydroxy bond donors and anion.*

Time trace graphs of ***5·OAc***

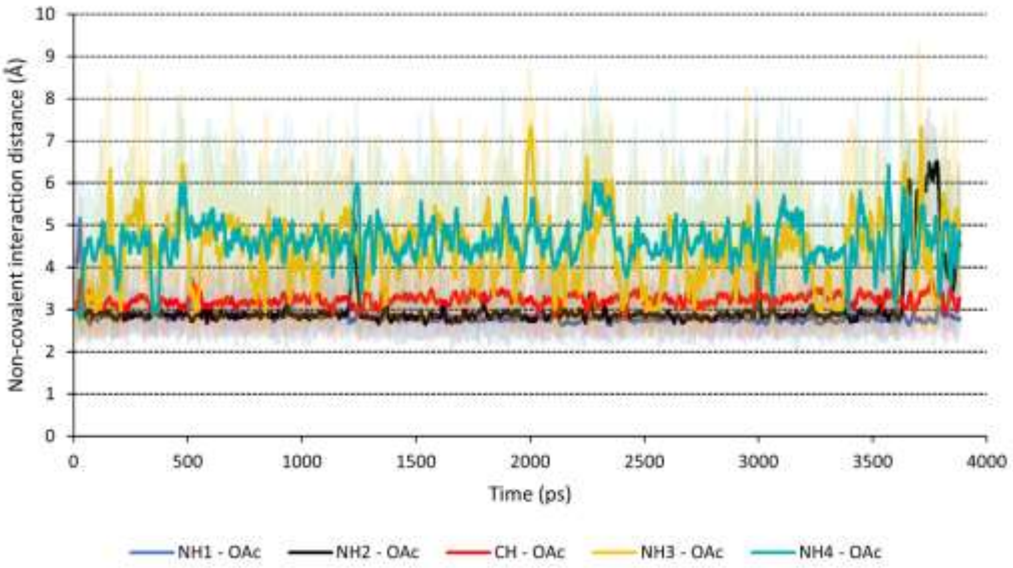

Figure S134. 5·OAc *time trace 1 of non-covalent interactions between hydrogen bond donors and anion.*

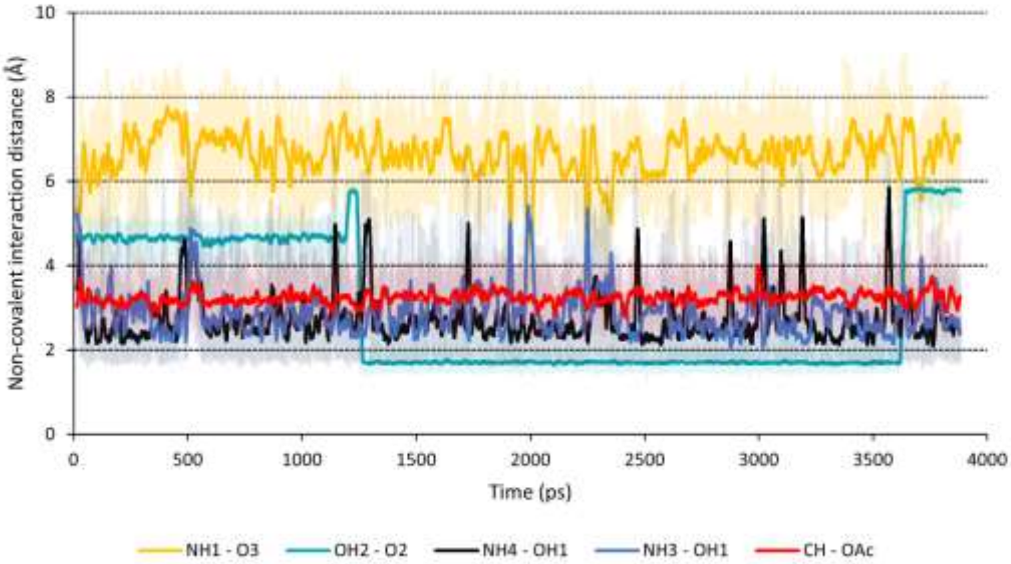

***Figure S135. 5·OAc** time trace 1 of non-covalent intercomponent interactions compared to the interior pyridinium proton distance to the anion (CH – OAc).*

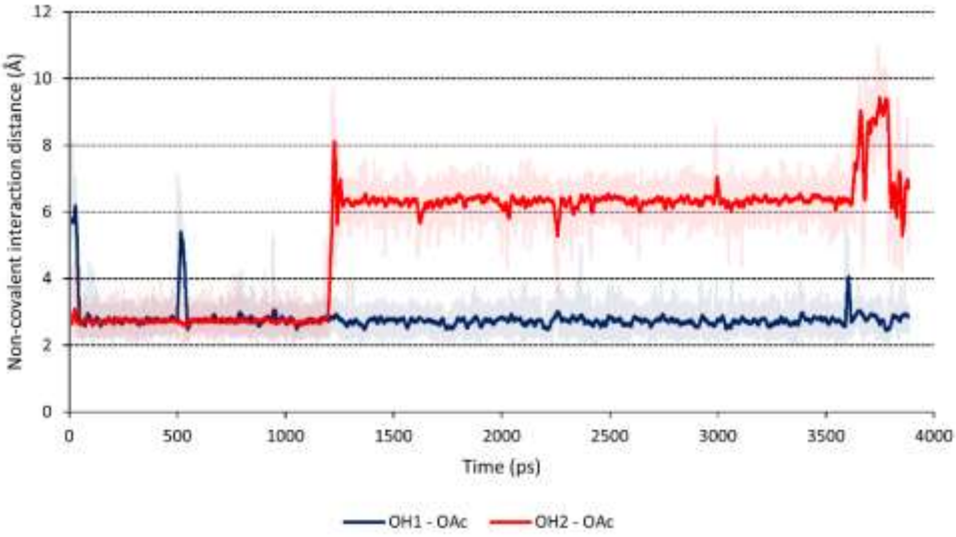

***Figure S136. 5·OAc** time trace 1 of non-covalent interactions between hydroxy bond donors and anion.*

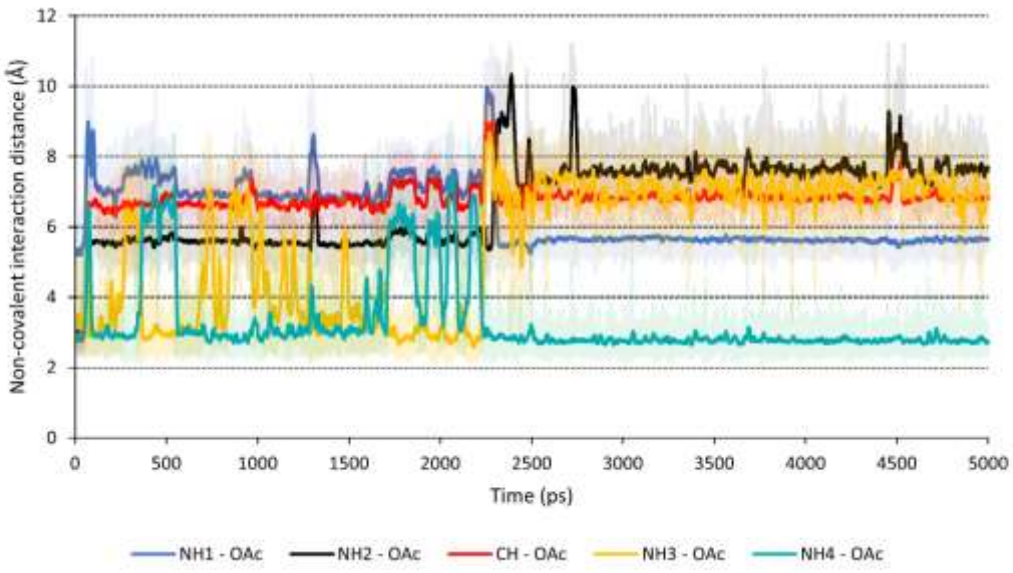

Figure S137. 5·OAc *time trace 2 of non-covalent interactions between hydrogen bond donors and anion.*

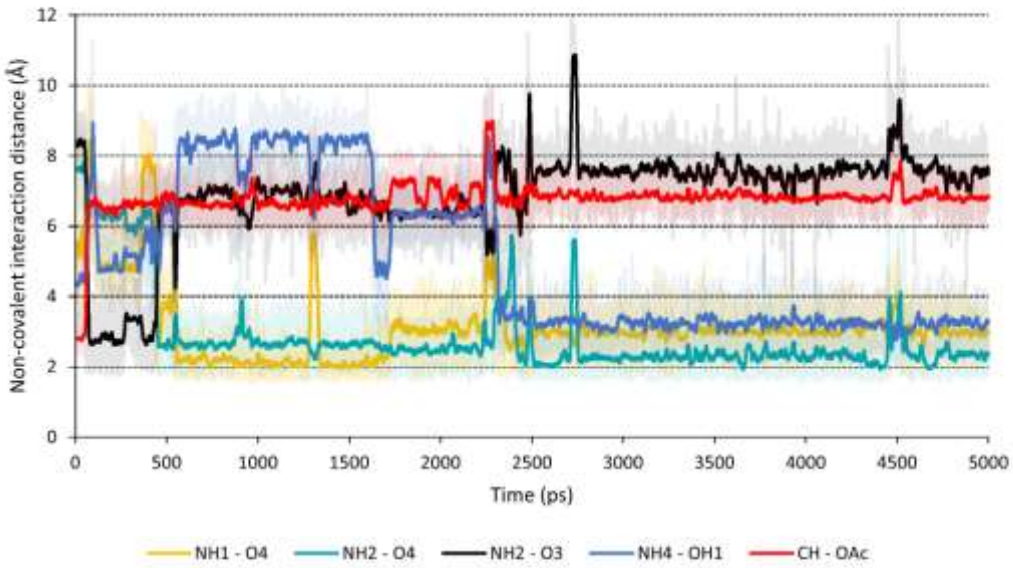

Figure S138. 5·OAc *time trace 2 of non-covalent intercomponent interactions compared to the interior pyridinium proton distance to the anion (CH – OAc).*

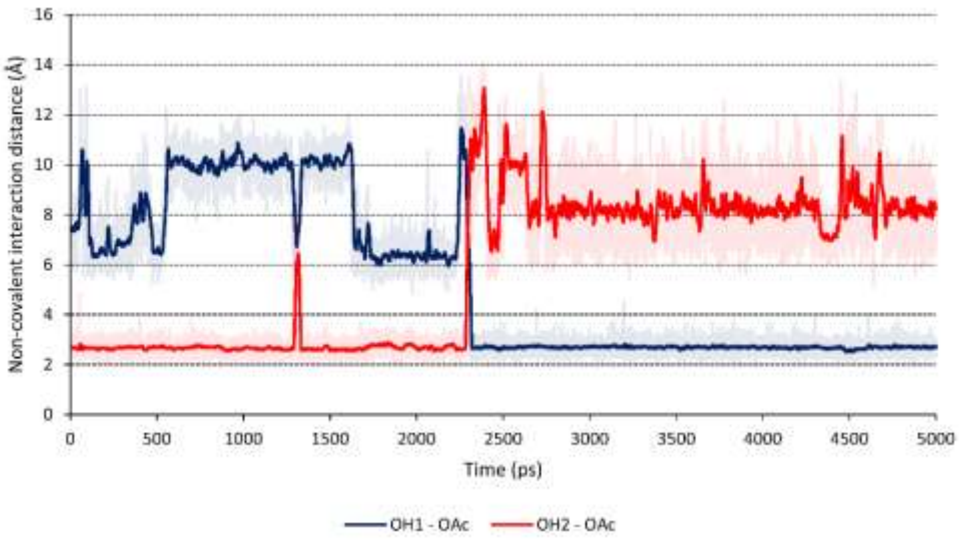

Figure S139. 5·OAc *time trace 2 of non-covalent interactions between hydroxy bond donors and anion.*

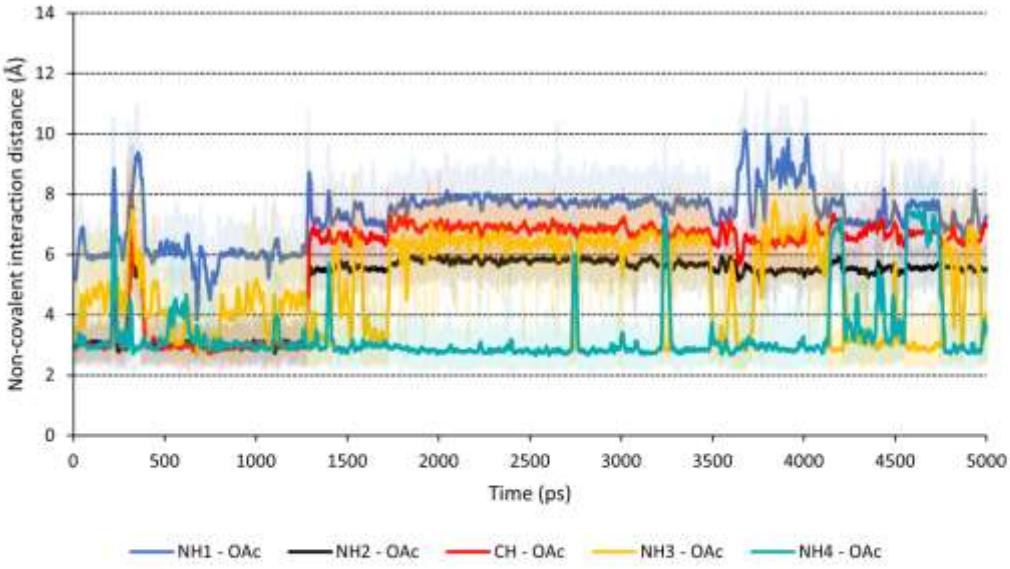

Figure S140. 5·OAc *time trace 3 of non-covalent interactions between hydrogen bond donors and anion.*

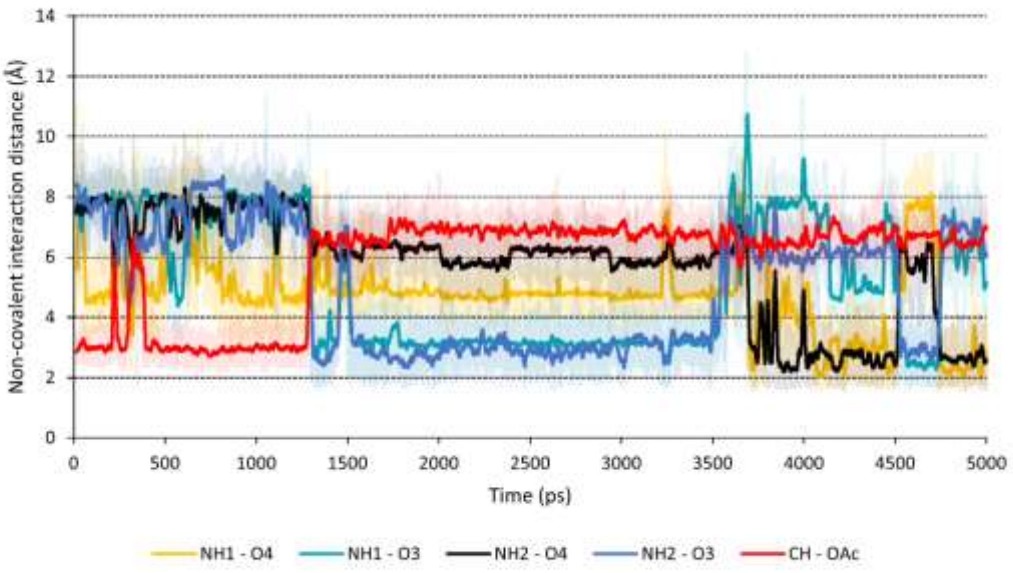

Figure S141. 5·OAc *time trace 3 of non-covalent intercomponent interactions compared to the interior pyridinium proton distance to the anion (CH – OAc).*

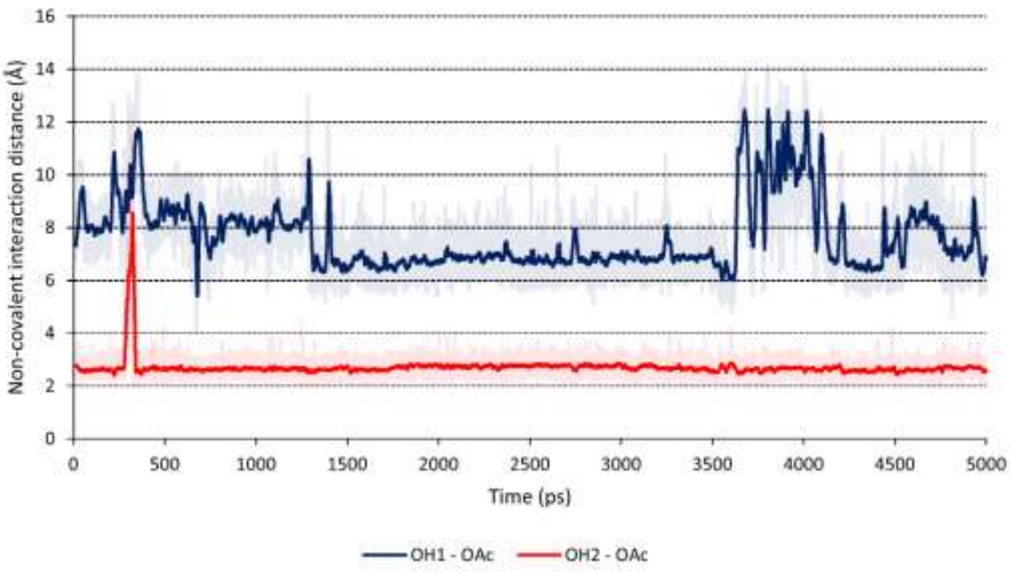

Figure S142. 5·OAc *time trace 3 of non-covalent interactions between hydroxy bond donors and anion.*

*Time trace graphs of **5·PF$_6$***

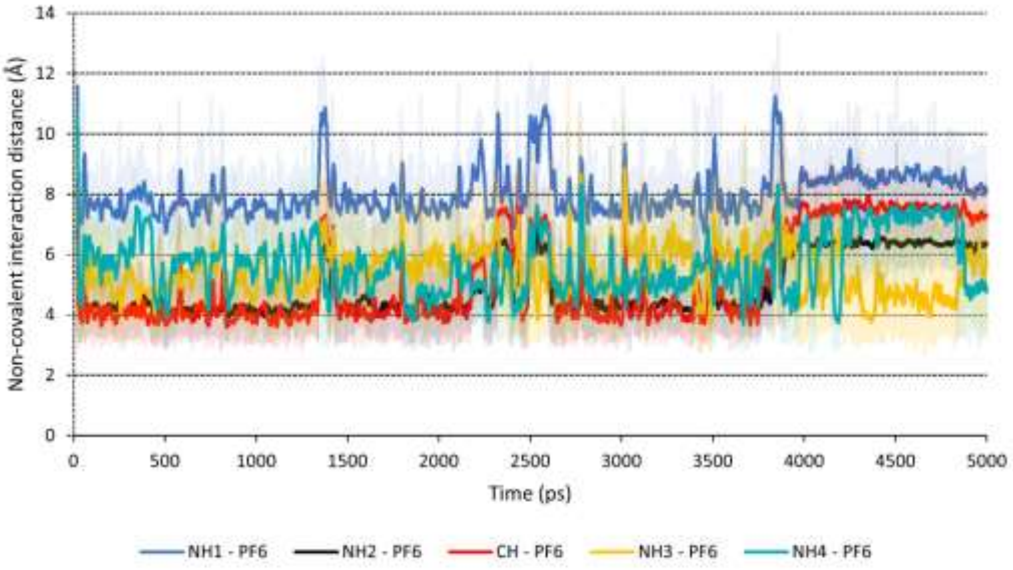

Figure S143. 5·PF$_6$ *time trace 1 of non-covalent interactions between hydrogen bond donors and anion.*

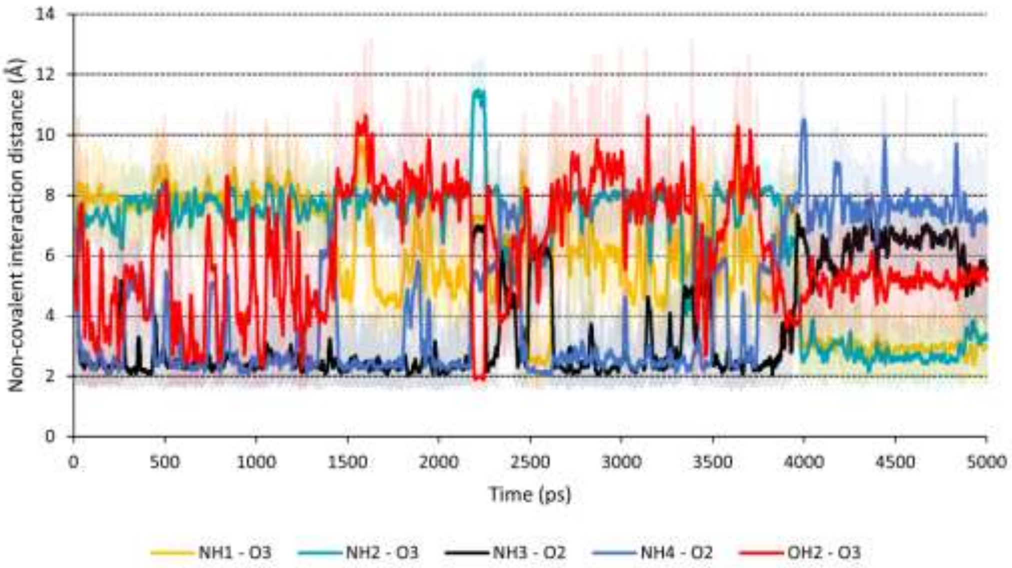

Figure S144. 5·PF$_6$ *time trace 1 of non-covalent intercomponent interactions.*

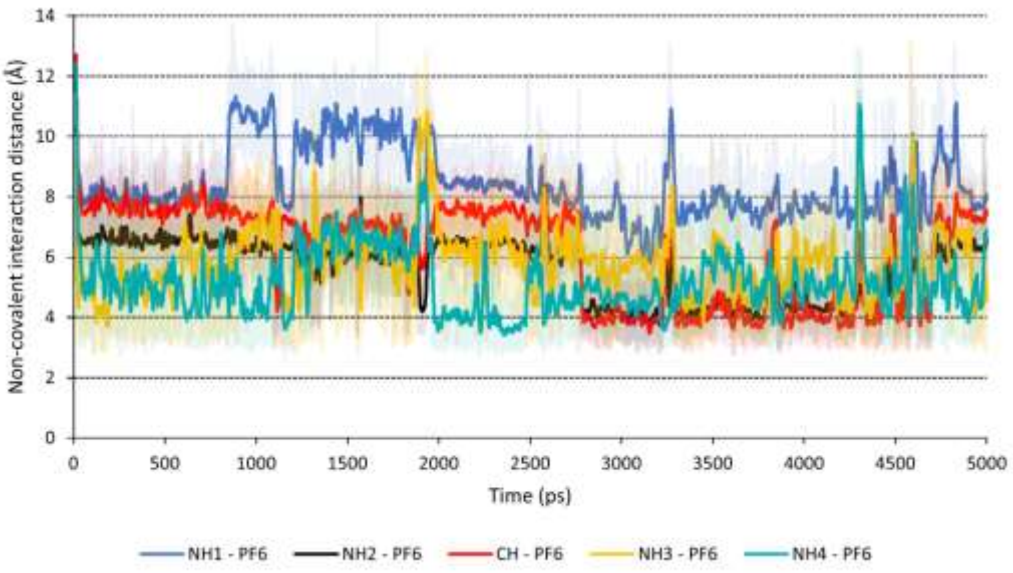

Figure S145. 5·PF$_6$ *time trace 2 of non-covalent interactions between hydrogen bond donors and anion.*

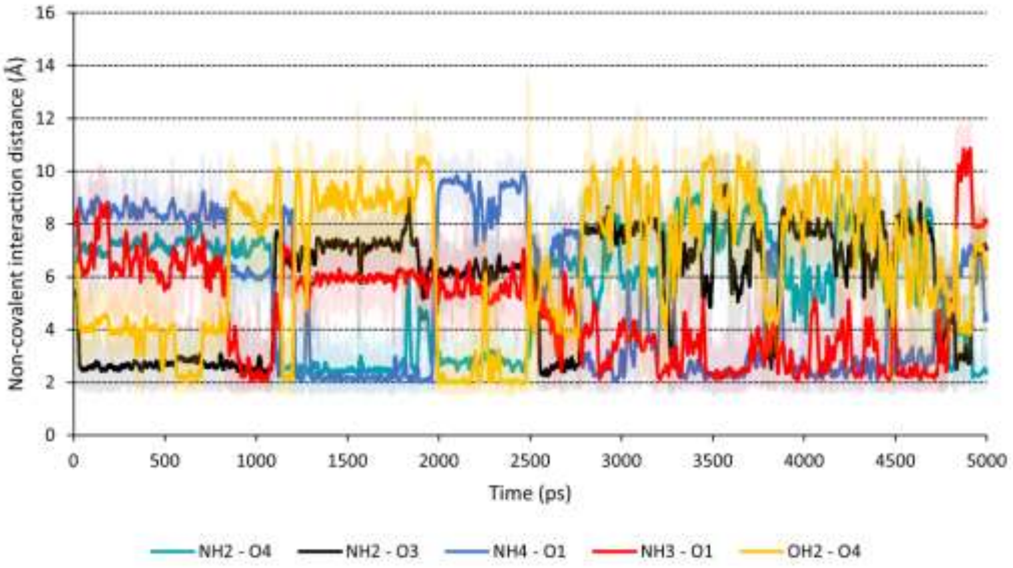

Figure S146. 5·PF$_6$ *time trace 2 of non-covalent intercomponent interactions.*

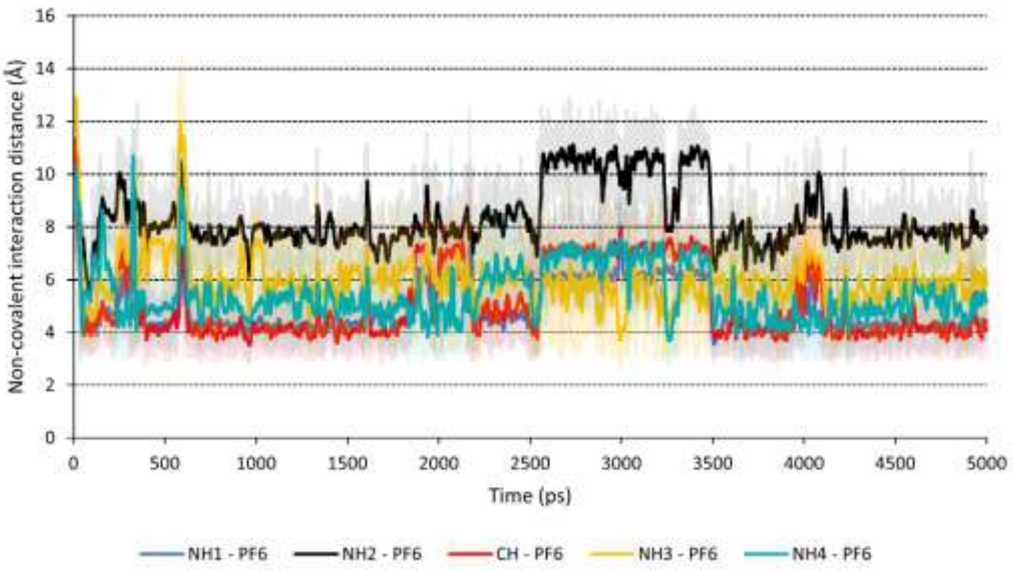

***Figure S147. 5·PF$_6$** time trace 3 of non-covalent interactions between hydrogen bond donors and anion.*

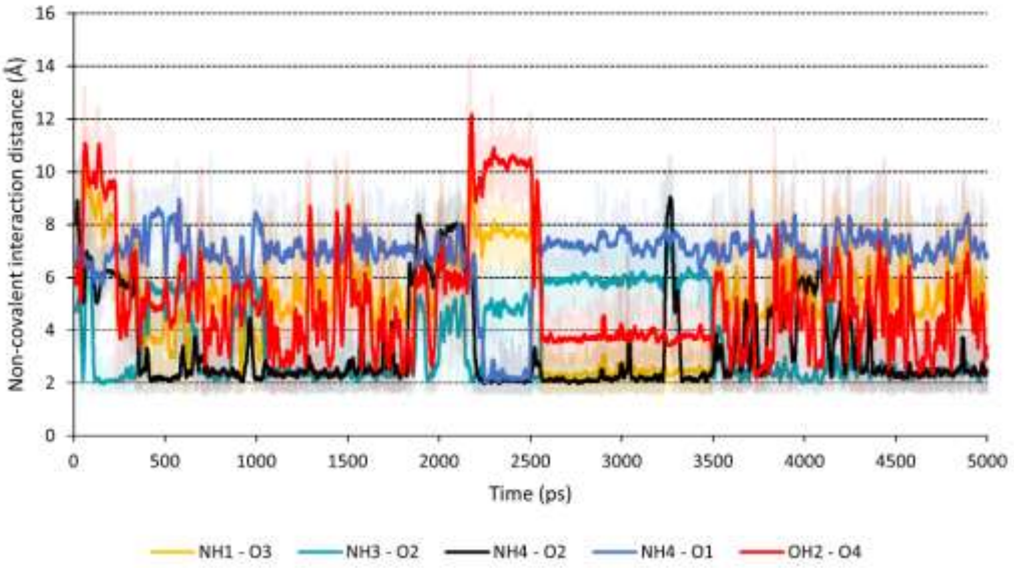

***Figure S148. 5·PF$_6$** time trace 3 of non-covalent intercomponent interactions.*

References

1 P. Norcott and C. S. P. McErlean, *Org. Biomol. Chem.*, 2015, **13**, 6866–6878.
2 H. W. Gibson, S.-H. Lee, P. T. Engen, P. Lecavalier, J. Sze, Y. X. Shen and M. Bheda, *J. Org. Chem.*, 1993, **58**, 3748–3756.
3 P. D. Frischmann, B. J. Sahli, S. Guieu, B. Patrick and M. J. MacLachlan, *Chem. Eur. J.*, 2012, **18**, 13712–13721.
4 M. R. Sambrook, P. D. Beer, M. D. Lankshear, F. Ludlow and J. A. Wisner, *Org. Biomol. Chem.*, 2006, **4**, 1529–1538.
5 H. E. Gottlieb, V. Kotlyar and A. Nudelman, *J. Org. Chem.*, 1997, **62**, 7512–7515.
6 W. He, F. Du, Y. Wu, Y. Wang, X. Liu, H. Liu and X. Zhao, *J. Fluorine Chem.*, 2006, **127**, 809–815.
7 A. O. Mattes, D. Russell, E. Tishchenko, Y. Liu, R. H. Cichewicz and S. J. Robinson, *Concepts Magn. Reson. A*, 2016, **45A**, e21422.
8 G. Chuchani and J. Zabicky, *J. Chem. Soc. C*, 1966, 297–299.
9 P. Thordarson, J. Wilmot and V. Efremova, http://supramolecular.org/.
10 N. P. Cowieson, D. Aragao, M. Clift, D. J. Ericsson, C. H. Gee, J. Stephen, N. Mudie, S. Panjikar, J. R. Price, A. Riboldi-Tunnicliffe, R. Williamson and T. Caradoc-Davies, *J. Synchrotron Radiat.*, 2015, **22**, 187–190.
11 W. Kabsch, *J. Appl. Crystallogr.*, 1993, **26**, 795–800.
12 *CrysAlis Pro*, Oxford Diffraction, 2011.
13 L. Palatinus and G. Chapuis, *J. Appl. Crystallogr.*, 2007, **40**, 786–790.
14 P. W. Betteridge, J. R. Carruthers, R. I. Cooper, K. Prout and D. J. Watkin, *J. Appl. Crystallogr.*, 2003, **36**, 1487.
15 C. Bannwarth, S. Ehlert and S. Grimme, *J. Chem. Theory Comput.*, 2019, **15**, 1652–1671.
16 Y. Shao, Z. Gan, E. Epifanovsky, A. T. B. Gilbert, M. Wormit, J. Kussmann, A. W. Lange, A. Behn, J. Deng and X. Feng, *Mol. Phys.*, 2015, **113**, 184–215.
17 G. Sigalov, A. Fenley and A. Onufriev, *J. Chem. Phys.*, 2006, **124**, 124902.
18 V. I. Lebedev, *Sib. Math. J.*, 1977, **18**, 99–107.
19 J.-P. Ryckaert, G. Ciccotti and H. J. C. Berendsen, *J. Comput. Phys.*, 1977, **23**, 327–341.
20 P. Pracht, F. Bohle and S. Grimme, *Phys. Chem. Chem. Phys.*, 2020, **22**, 7169–7192.
21 W. Humphrey, A. Dalke and K. Schulten, *J. Mol. Graph.*, 1996, **14**, 33–38.
22 *CrystalMaker*®, CrystalMaker Software Ltd, Oxford, England, (www.crystalmaker.com).
23 S. Alvarez, *Dalton Trans.*, 2013, **42**, 8617–8636.
24 M. Morshedi, S. Boer, M. Thomas and N. G. White, *Chem. Asian. J.*, 2019, **14**, 1271–1277.
